# Covalent Organic Framework–Carbon Nanotube Core–Shell Nanohybrids for Enhanced Catalytic Site Utilization of Molecular Catalysts in CO_2_ Electroreduction

**DOI:** 10.1002/anie.202521776

**Published:** 2026-03-19

**Authors:** Liang Yao, Andrés Rodríguez‐Camargo, Roman Guntermann, Fabian Heck, Samuel Van Gele, Hugo Vignolo‐González, Viola Duppel, Thomas Bein, Bettina V. Lotsch

**Affiliations:** ^1^ State Key Laboratory of Luminescent Materials and Devices Institute of Polymer Optoelectronic Materials and Devices Guangdong Basic Research Center of Excellence for Energy and Information Polymer Materials, Guangdong Provincial Key Laboratory of Luminescence from Molecular Aggregates South China University of Technology Guangzhou China; ^2^ Nanochemistry Department Max Planck Institute for Solid State Research Stuttgart Germany; ^3^ Department of Chemistry University of Stuttgart Stuttgart Germany; ^4^ Department of Chemistry and Center for NanoScience (CeNS) Ludwig‐Maximilians‐Universität München Munich Germany; ^5^ E‐conversion and Center for Nanoscience Garching bei München Germany

**Keywords:** carbon nanotube, covalent organic framework, electrochemical CO_2_ reduction, nanohybrid

## Abstract

Developing strategies to enhance the utilization efficiency of catalytic sites in molecular catalysts has garnered increasing research interest in the field of molecular heterogeneous catalysis. The primary challenges in achieving this goal lie in the aggregation‐induced site inaccessibility in molecular catalysts. Here, we present the synthesis of covalent organic framework‐carbon nanotube (COF‐CNT) core‐shell nanohybrids as a platform to improve the site utilization of molecular catalysts in electrochemical CO_2_ reduction. COF shells with a thickness of 50–80 nm are uniformly grown on CNTs, ensuring a well‐defined morphology with pores oriented perpendicularly to the CNT basal plane. The incorporation of molecular catalysts with COF‐CNT nanohybrids enables their application as scaffolds in the electrochemical CO_2_ reduction. The best‐performing sample exhibits a two‐orders‐of‐magnitude increase in CO turnover frequency (TOF) compared to both pristine CoTPyP molecular catalyst and COF‐366‐Co, thus underscoring the effectiveness of the COF‐CNT hybrid structure in optimizing catalytic site accessibility. The enhanced site utilization is further validated in other molecular catalyst systems, where exceptionally high TOF values—among the highest reported to date for electrochemical CO_2_‐to‐CO conversion—were achieved. Collectively, this study establishes COF‐CNT nanohybrids as a promising strategy for advancing COF‐based electrocatalysts and facilitating molecular catalyst applications in electrochemical energy conversion.

## Graphical abstract

Covalent organic framework–carbon nanotube (COF–CNT) core–shell nanohybrids are developed as an efficient platform to enhance the site utilization of molecular catalysts for electrochemical CO_2_ reduction. The well‐defined nanostructure promotes catalytic site accessibility, achieving CO turnover frequencies among the highest reported to date.

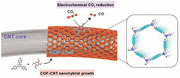

## 1 Introduction

Since Taylor introduced the notion of the active site on a catalytic surface in 1925, the rational engineering of catalytic sites has become an appealing yet challenging task in the field of heterogeneous catalysis [1, 2, 3, 4]. Over the past few decades, molecular catalysts have been extensively explored for heterogeneous catalysis, given their tunable chemical and electronic structures, and have demonstrated competitive performance for electrochemical CO_2_ conversion compared to other types of catalysts, such as gold, silver, and other metal‐based catalysts [5, 6, 7]. Nevertheless, a recurring issue with conventional molecular catalysts lies in their limited efficiency in utilizing catalytic sites when forming a solid catalyst [8]. This inefficiency arises from the formation of molecular catalyst aggregates resulting from intermolecular interactions, thereby rendering catalytic sites inaccessible to reactants and electrolytes. Furthermore, the intrinsically low electronic conductivity of molecular catalysts results in poor electron transport, especially in the presence of large molecular aggregates, further diminishing the utilization of their catalytic sites. Indeed, the low catalytic site utilization efficiency is a common problem for heterogeneous catalysts, and is often seen in a range of catalysts, including metals, metal oxides, etc. Consequently, in recent years, there has been a growing interest in exploring innovative strategies aimed at optimizing the utilization of catalytic sites [6, 9, 10, 11].

Covalent organic frameworks (COFs) constitute an emerging class of porous polymers, notable for their large surface areas and the capacity for precision customization through linking diverse building blocks [12, 13]. Indeed, the large surface areas of COFs hold promise for the immobilization of molecular catalysts, and the porosity of COFs facilitates mass transport processes which are key in electrocatalysis. However, typical COFs are often synthesized as intergrown secondary particles ranging in size from hundreds of nanometers to micrometers, resulting from the aggregation of nanocrystallites. This undefined morphological structure, on the one hand, causes the electronic isolation of catalytic sites inside the COF particles, detrimental to their utility in electrocatalysis. On the other hand, the molecular catalysts can clog the surface of COF nanocrystallite aggregates, resulting in a limited loading capacity for these catalysts. An alternative strategy for integrating catalytically active molecules in COFs is to design porphyrin or phthalocyanine‐based COFs, where the molecular catalysts serve as the COF building block. Since Yaghi and co‐workers first reported metallated porphyrin‐linked COFs for electrochemical CO_2_ reduction catalysts in 2015, a number of metallated porphyrin and phthalocyanine‐linked COFs have been developed [14, 15, 16, 17, 18, 19, 20]. Nevertheless, these COFs are typically µm‐sized particles that still suffer from electronic isolation, as we recently reported [21]. To effectively address these challenges and harness COFs for engineering the catalytic sites of molecular catalysts, it is crucial to develop COF systems with well‐defined morphologies.

**FIGURE 1:**
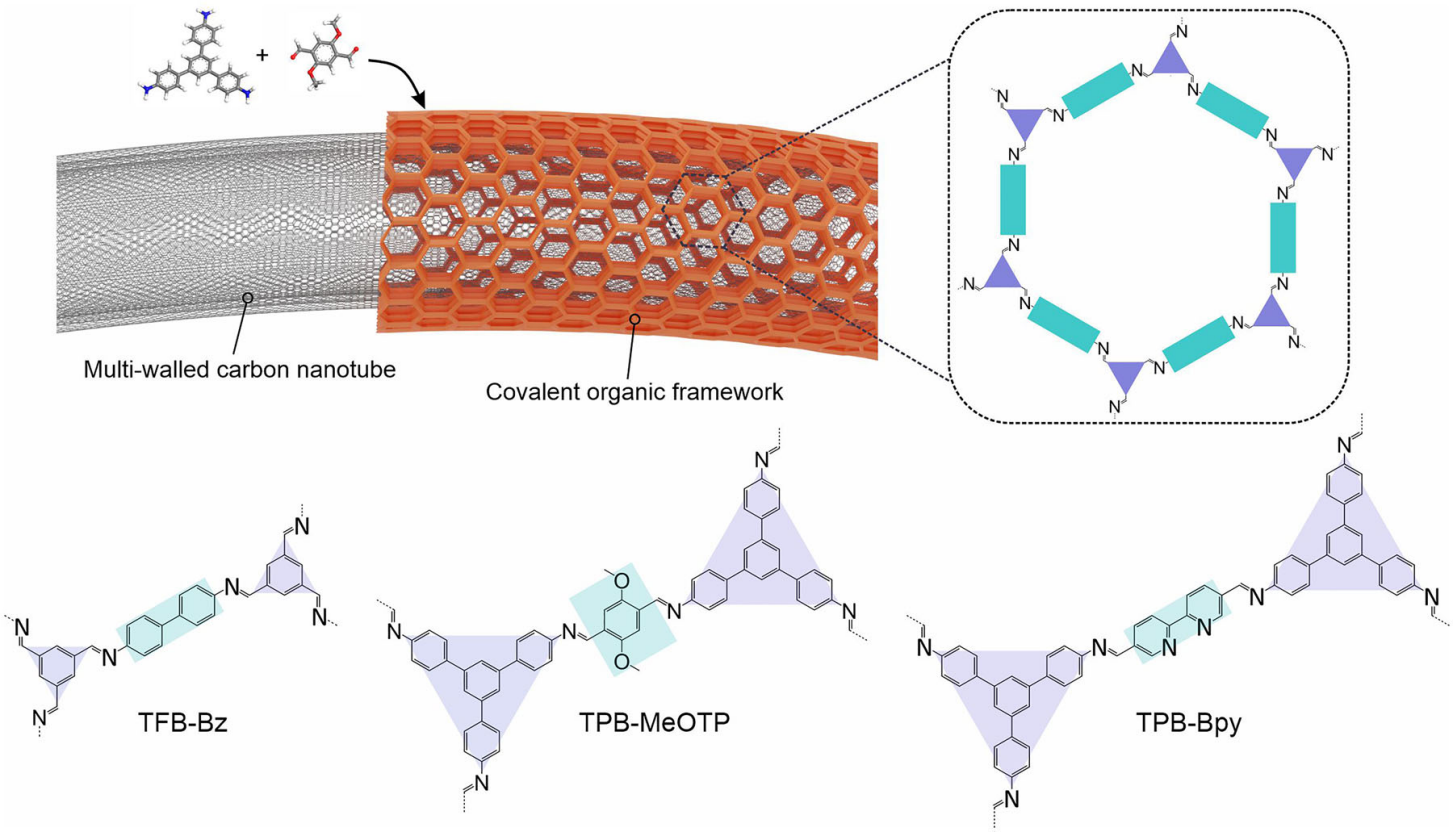
Schematic illustration of COF‐carbon nanotube (CNT) core‐shell nanohybrids and chemical structure of TFB‐Bz, TPB‐MeOTP, and TPB‐Bpy.

Herein, we devised a series of COF‐carbon nanotube (CNT) core‐shell nanohybrids with a COF shell thickness of 50–80 nm and a configuration in which the COF layer is oriented parallel to the CNT substrate (Figure 1). The integration of COFs with carbon nanotubes (CNTs) has garnered considerable research attention, with a growing number of studies reporting COF/CNT composites and demonstrating their superior applications in lithium‐ion batteries, photoresponsive batteries, etc. [22, 23, 24, 25, 26, 27]. However, these reports employ the classical solvothermal route for composite synthesis, yielding undefined COF particles as the product. Since both COFs and CNTs as well as the COF/CNT composites are inherently insoluble, classical solvothermal conditions only provide mixtures and effective solutions to separate the components are lacking, thus precluding morphologically defined COF systems. In this work, the synthesis of our COF‐CNT nanohybrids is achieved through a direct island‐nucleation and growth on the CNT, which leverages the colloidal COF synthesis approach recently developed in the field [28, 29, 30, 31, 32]. Subsequently, molecular catalysts tailored for electrochemical CO_2_ reduction were incorporated into the COF shell by physisorption, resulting in a substantial enhancement in the utilization of catalytic sites, surpassing the performance of directly deploying the molecular catalyst as a heterogeneous catalyst, utilizing bare CNT as a support material, as well as COF‐366‐Co [14], a classical electrochemical CO_2_ reduction active COF with metallated porphyrin units as building blocks.

## 2 Results and Discussion

Covalent organic framework–carbon nanotube (COF‐CNT) nanohybrids were synthesized utilizing a colloidal COF synthesis approach, which was recently developed by Dichtel and co‐workers [28]. By combining mesitylene/dioxane and acetonitrile as the reaction solvents, highly crystalline imine‐linked COFs are accessible via homogeneous nucleation. Under optimized reaction conditions, well‐dispersed spherical COF nanoparticles were formed in the solution (Figures S1–S5), and a thin COF shell was conformally grown on the surface of the CNT (see below). After the reaction, the COF colloidal solution was extracted using a pipette, and the resultant COF/CNT nanohybrid was isolated through filtration (Figures S6, S7). To establish the general applicability of this methodology, we synthesized three COF‐CNT nanohybrid samples with different lattice parameters and, hence, pore sizes, including TFB‐Bz‐CNT, TPB‐MeOTP‐CNT and TPB‐Bpy‐CNT.

**FIGURE 2:**
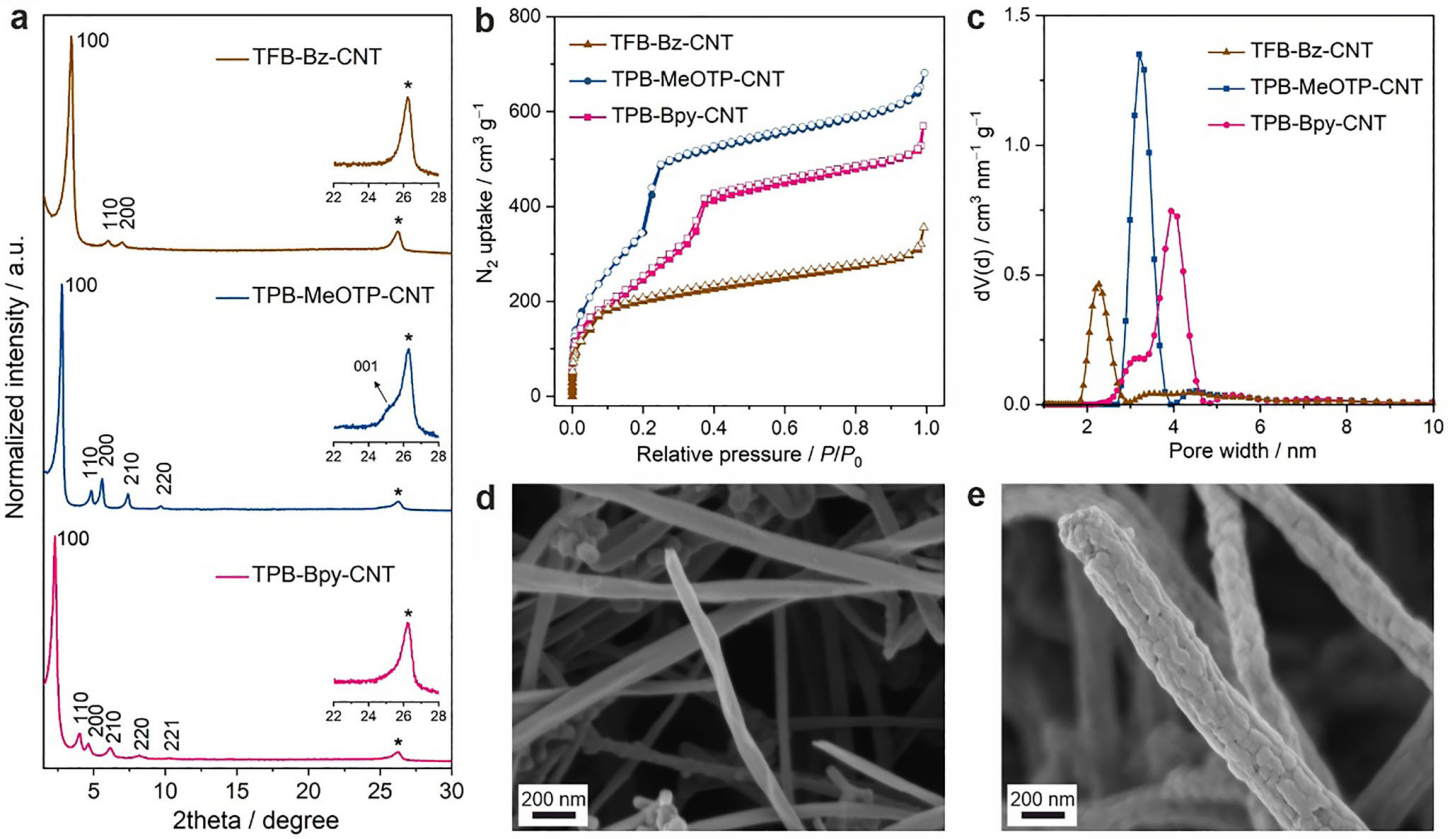
Crystallinity, porosity, and morphology characterizations of COF‐CNT nanohybrids, including TFB‐Bz‐CNT, TPB‐MeOTP‐CNT, and TPB‐Bpy‐CNT. (a) PXRD patterns (Cu‐K*α*_1_). (b) N_2_ adsorption (filled) and desorption (empty) isotherm profiles at 77 K. (c) Pore size distribution. (d, e) Top–down SEM images of bare CNT (d) and TPB‐MeOTP‐CNT (e).

**FIGURE 3:**
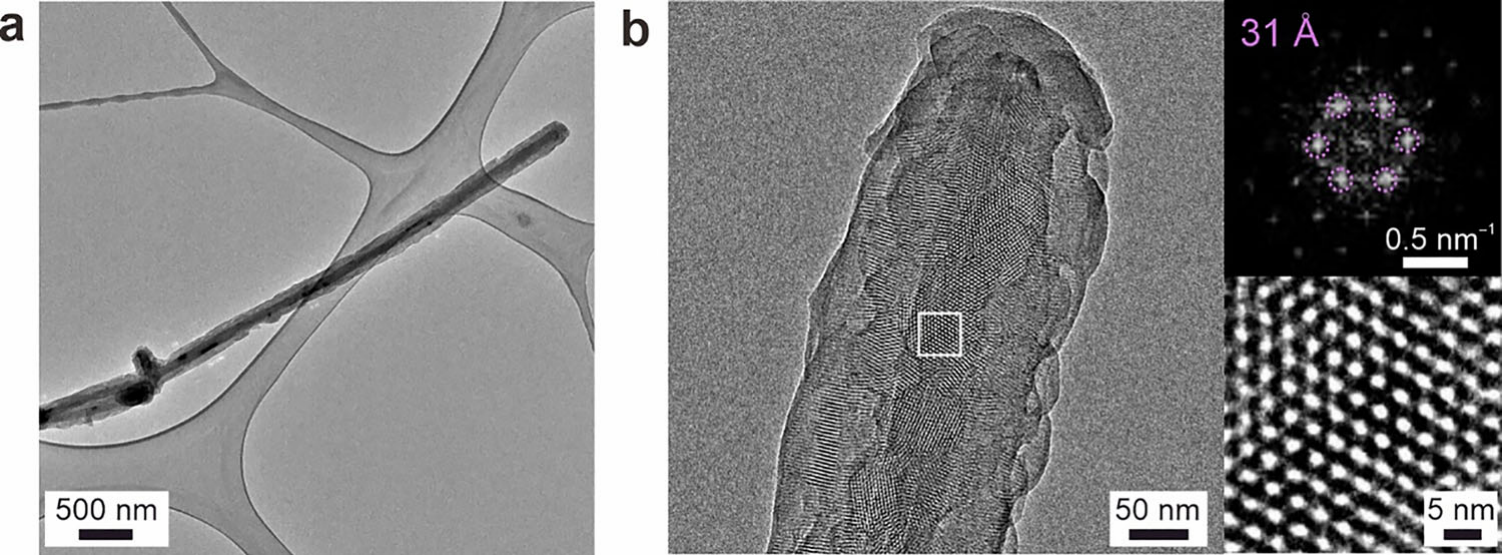
Bright field (BF)‐TEM images of TPB‐MeOTP‐CNT. FFT pattern and high‐resolution (HR) BF‐TEM image of the boxed region are displayed at the top right and bottom right of Figure 3b, respectively.

Structure analysis of these COF‐CNT nanohybrids via powder X‐ray diffraction (PXRD) reveals high crystallinity, indicated by the intense and sharp 100 reflections from the COFs at 2θ = 3.5° (TFB‐Bz‐CNT), 2.8° (TPB‐MeOTP‐CNT), and 2.3° (TPB‐Bpy‐CNT), and the presence of several other reflections at higher angles (Figure 2a). The experimental PXRD patterns are in good agreement with eclipsed stacked COF models (Figure S8). A control PXRD measurement of multi‐walled carbon nanotubes demonstrates that the reflection at 2θ = 26.2°, marked with an asterisk, in the COF‐CNT nanohybrids corresponds to the 002 planes of turbostratically stacked graphite sheets of multi‐walled carbon nanotubes (Figure S9). The 001 reflection of COFs, assigned to COF interlayer stacking, is much weaker compared to the 002 reflection of the multi‐walled carbon nanotube, likely due to planar stacking faults common in 2D COFs [33]. The porosity of COF‐CNT nanohybrids was investigated by nitrogen adsorption measurements at 77 K (Figure 2b). TPB‐MeOTP‐CNT and TPB‐Bpy‐CNT show type‐IV isotherms indicative of mesoporous materials, and the calculated pore‐size distribution indicates a pore size of 3.2 and 3.9 nm, respectively (Figure 2c). In comparison, TFB‐Bz‐CNT exhibits a type‐I(b) isotherm, due to the shorter linkers resulting in a smaller pore size (2.3 nm as suggested by the calculated pore‐size distribution in Figure 2c). The Brunauer−Emmett−Teller surface areas (*S*_BET_) are 704, 1363, and 946 m^2^ g^−1^ for TFB‐Bz‐CNT, TPB‐MeOTP‐CNT and TPB‐Bpy‐CNT, respectively (Figures S10–S12). As the mass of the COF‐CNT nanohybrid (29.9 mg, 34.2 mg, and 26.4 mg for TPB‐MeOTP‐CNT, TPB‐Bpy‐CNT, and TPB‐Bz‐CNT, respectively) is nearly double that of the initial CNT (15 mg) used for synthesis, with approximately equal masses for the CNT core and COF shell, the actual S_BET_ of the COF shell is estimated to be roughly two times higher than the measured value. It is worth noting that the S_BET_ of the bare CNT is only 41 m^2^ g^−1^ and that it does not exhibit the characteristic pore size distribution observed in the COF‐CNT nanohybrids, which confirms that the high surface area and porosity of COF‐CNT nanohybrids originate from the COF shell rather than the CNT core (Figure S13). Fourier transform infrared (FT‐IR) spectra of the COF–CNT nanohybrids exhibit band positions similar to those of the corresponding COF nanoparticles, with a characteristic C═N stretching vibration observed in all three samples (Figure S14). These spectroscopic features confirm successful imine bond formation and the construction of structurally analogous COF frameworks within the COF–CNT nanohybrids. Scanning electron microscopy (SEM) and transmission electron microscopy (TEM) were employed to examine the morphology of COF‐CNT nanohybrids. SEM images show that the COF–CNT nanohybrids contain minimal contamination of COF nanoparticles, indicating successful synthesis without heavy mixing of free COF nanoparticles (Figures 2e and S15–S18). The average diameter of COF‐CNT nanohybrids, ∼170 nm of TFB‐Bz‐CNT, ∼210 nm of TPB‐MeOTP‐CNT and ∼160 nm of TPB‐Bpy‐CNT, is significantly increased compared to that of bare CNTs (∼80 nm) (Figure 2d). Moreover, it can be observed that, compared to the featureless CNT surface, the COF‐CNT nanohybrids exhibit granular textures at the surface corresponding to the COF grains. The nanoscale core‐shell structure of COF‐CNT nanohybrids is further revealed by bright field (BF)‐TEM images as well as fast Fourier transform (FFT) patterns extracted from BF‐TEM images. Figure 3a displays that TPB‐MeOTP‐CNT is composed of a CNT core with a diameter of ∼60 nm and a surrounding conformal shell of TPB‐MeOTP COF. Given the uniform and continuous coverage of the CNT core by the COF shell, it can be inferred that COF nucleation occurs directly on the CNT substrate rather than via the formation of discrete nanoparticles in solution followed by their subsequent deposition onto the CNT surface. This latter scenario would result in a rougher surface, as the nanoparticles formed in solution typically exhibit sizes on the scale of hundreds of nanometers. A magnified image of the CNT core shows the periodic spacing of 3.4 Å (Figure S19), assigned to the 002 lattice planes of the multi‐walled CNT. Moreover, Figure 3b indicates that the thickness of the TPB‐MeOTP COF shell is approximately 80 nm and consists of highly crystalline domains. The FFT pattern in Figure 3b shows a lattice spacing of 3.1 nm, which is consistent with the 100 reflection of TPB‐MeOTP COF and further confirms the formation of a TPB‐MeOTP COF shell on the CNT. It is noteworthy that crystallite domains of TPB‐MeOTP COF, characterized by hexagonal FFT patterns, can be observed right above the CNT core. This suggests that TPB‐MeOTP COF preferentially grows with the orientation of the hexagonal pore aligning horizontally with the underlying multi‐walled CNT core, while the pore channels run perpendicularly. Other regions and different batches of TPB‐MeOTP‐CNT exhibiting the same behavior are summarized in Figures S20–S22. Figures S23 and S24 display the nanoscale morphology of TFB‐Bz‐CNT and TPB‐Bz‐CNT, where similar core‐shell structures can be observed.

**FIGURE 4:**
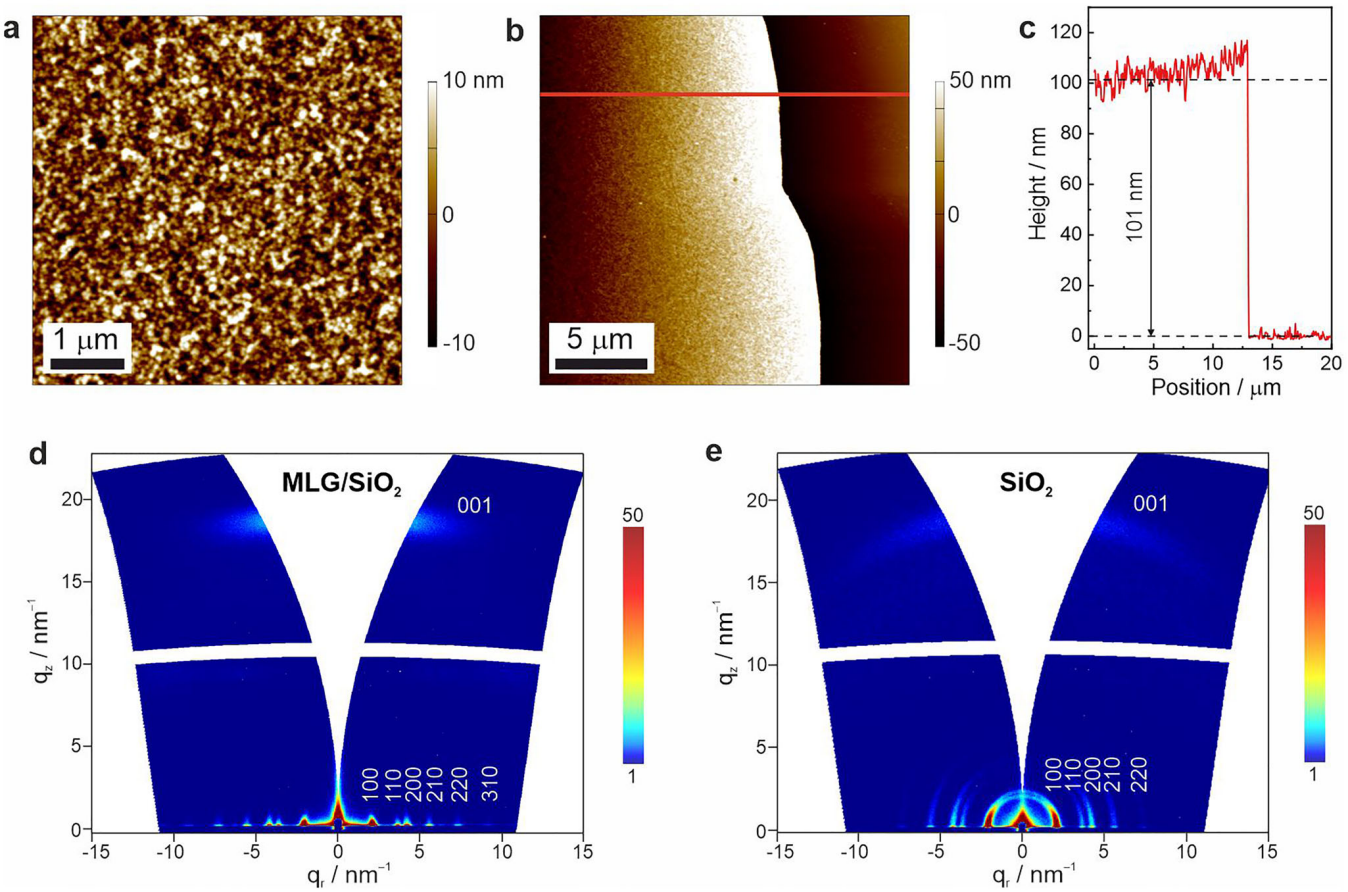
Investigation of oriented COF growth on graphene. (a) AFM height image of the TPB‐MeOTP film on MLG/SiO_2_ substrate. (b, c) AFM measurement for determining TPB‐MeOTP film thickness, including height image (b) and the height plot along the red line (c). To expose the MLG SiO_2_/Si substrate, the right side of the TPB‐MeOTP film in the AFM height image was removed by mechanical scratching. (d, e) Grazing incidence wide‐angle X‐ray scattering two‐dimensional (GIWAXS 2D) patterns of TPB‐MeOTP film grown on an MLG SiO_2_/Si wafer (d) and a bare SiO_2_/Si wafer (e) obtained via identical synthesis conditions.

Further insight into the oriented COF growth on CNTs was obtained by growing COF thin films on a monolayer graphene (MLG) SiO_2_/Si wafer under identical reaction conditions used for COF‐CNT nanohybrid synthesis. Considering that multi‐walled CNTs can be conceptualized as cylindrical structures resulting from the rolling‐up of multi‐layer graphene sheets, it is anticipated that the surface of multi‐walled CNTs and MLG SiO_2_/Si wafers would behave in a similar manner for supporting the templated growth of these three COFs, if such behavior exists. The morphology and the crystallinity of the resultant COF thin films were investigated by atomic force microscopy (AFM) and grazing incidence wide‐angle X‐ray scattering (GIWAXS), respectively. As shown in Figures 4a, S25, and S26, smooth thin films are obtained on MLG SiO_2_/Si wafer for all the three COFs with a root‐mean‐square (RMS) roughness of 3.8 nm for TFB‐Bz, 4.8 nm for TPB‐MeOTP and 1.8 nm for TPB‐Bpy, demonstrating that these films have smoothness similar to highly efficient solution‐processed organic semiconducting films for energy conversion devices [34]. The thin film thickness is determined to be 144 nm for TFB‐Bz, 101 nm for TPB‐MeOTP and 79 nm for TPB‐Bpy by AFM height plots (Figure 4b, c), slightly higher than the COF shell thickness on the CNT core. This difference may arise from a combination of factors, including possible diffusion limitations during COF growth on the CNT surface as well as differences in substrate geometry, surface accessibility, and local monomer availability between the planar MLG/SiO_2_ substrate and the curved CNT surface. In addition, GIWAXS data demonstrate that the COF films are preferentially oriented on the MLG SiO_2_/Si wafer with the COF pores perpendicular to the substrate (Figure 4d, e). For instance, TPB‐MeOTP films on MLG SiO_2_/Si show low arching in‐plane reflections at *q_r_* = 2.1, 3.6, 4.2, 5.5, 7.3, and 9.1 nm^‒1^, corresponding to the 100, 110, 200, 210, 220, and 310 reflections, respectively, and an intense out‐of‐plane reflection centered at *q*_z_ ≈ 18.7 nm^‒1^, attributed to the 001 plane. In comparison, using a bare SiO_2_/Si wafer as substrate, increased arching is observed for in‐plane reflections, and the out‐of‐plane reflection becomes weaker. Meanwhile, oriented COF film growth on MLG SiO_2_/Si wafers is also found for TFB‐Bz and TPB‐Bpy. As shown in Figures S27 and S28, TFB‐Bz and TPB‐Bpy films on MLG SiO_2_/Si wafers also exhibit low arching in‐plane reflections, while in contrast, the bare SiO_2_/Si wafer does not afford visible in‐plane reflections for these two COFs. The above results clearly indicate that the graphene surface favors the preferentially oriented growth of the imine COFs under these specific reaction conditions, in accordance with the TEM results, further supporting the oriented COF shell growth on CNTs. We further found that the diffusion of the monomers and Schiff‐base reaction catalyst acetic acid plays a crucial role in the COF‐CNT nanohybrid synthesis. Another type of CNT with a diameter of 13 nm (CNT‐13) was used to synthesize COF‐CNT nanohybrids under identical reaction conditions. Although crystalline COFs are also obtained, SEM images suggest that the COF tends to grow across the CNT‐13 located in close proximity, rather than forming well‐defined COF‐CNT core‐shell nanostructures. This is because CNT‐13 only has very limited accessible space between the nanotubes, which hampers the diffusion of monomers and acetic acid, and correspondingly, prevents conformal COF growth on individual CNTs (see details in Figures S29).

**FIGURE 5:**
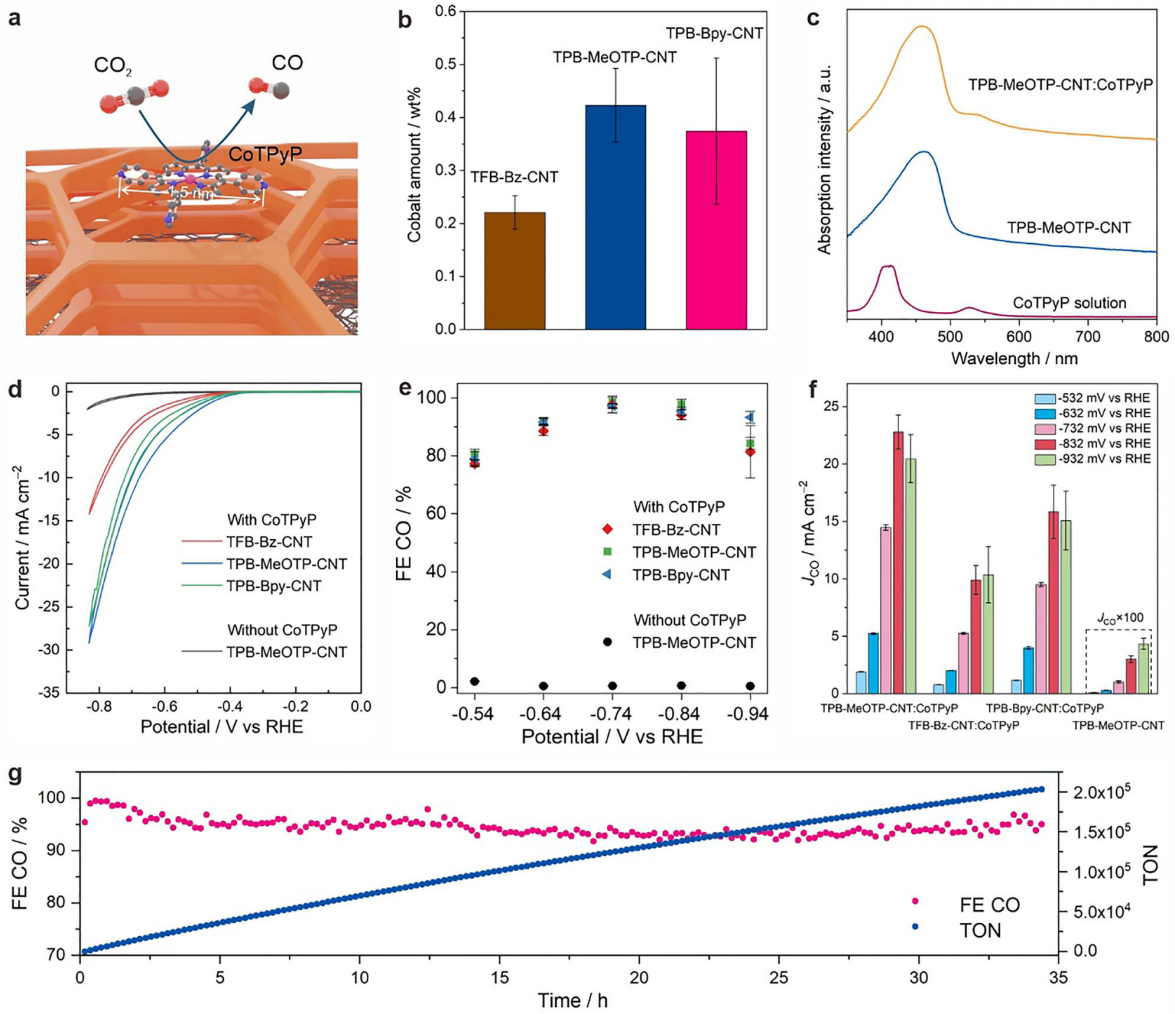
Electrochemical CO_2_ reduction performance in 0.5 M KHCO_3_ under CO_2_ flow. (a) Schematic illustration of CoTPyP physisorbed COF‐CNT nanohybrids for electrochemical CO_2_ conversion. (b) Cobalt amount of COF‐CNT nanohybrids after the physisorption of CoTPyP determined by ICP‐OES measurements. Six measurements from three independent batches of samples were conducted for TPB‐MeOTP‐CNT:CoTPyP with the error bars representing the standard deviations. For the other two samples, four measurements from two independent batches were conducted. (c) Comparison of UV‐vis spectra of TPB‐MeOTP‐CNT:CoTPyP and TPB‐MeOTP‐CNT suspended in ethanol, and CoTPyP in chloroform solution. (d, e) Cyclic voltammograms (d) at the scan rate of 5 mV s^−1^ and FE(CO) (e) of COF‐CNTs: CoTPyP, and TPB‐MeOTP‐CNT without incorporating CoTPyP. (f) Partial current densities for CO production (*J*_CO_) at different potentials. Standard deviations in 5e and f were obtained from four measurements. (g) Long‐term stability of the TPB‐MeOTP‐CNT:CoTPyP operated at ‒0.73 V vs. RHE for 34 h.

Having characterized the nanoscale structure of the COF‐CNT nanohybrids, we next sought to apply them in electrochemical CO_2_ reduction by incorporating molecular catalysts into COF‐CNT nanohybrids (Figure 5a). Cobalt porphyrins and phthalocyanines have been recognized as promising electrocatalysts for CO_2_ conversion. In addition, it has been demonstrated that small molecules or dyes possessing a significantly smaller molecular size could infiltrate the COF pores, which has enabled applications of COFs associated with drug delivery and dye separation [35]. We thus hypothesized that when molecular catalysts with suitable molecular sizes are incorporated into the COF pores, aggregation of the molecular catalysts can be prevented through confinement‐induced strong interactions between the COF pore wall and the molecular catalyst. At the same time, the catalytic centers remain accessible to the reactants thanks to the highly porous structure of the COF‐CNT nanohybrids, thus providing a path to increase the utilization efficiency of the catalytic centers. To test this hypothesis, we conducted a comprehensive investigation using the cobalt porphyrin molecular catalyst CoTPyP and further extended to other porphyrin‐ and phthalocyanine‐based molecular catalysts, including CoPc and CoTPP, all of which exhibit a strong tendency to aggregate at solid state [8] (Figure S30). It is noted that all of them have a molecular size of approximately 1.5 nm as indicated in Figure S30, smaller than the pore size of the aforementioned COF‐CNT nanohybrids. These molecular catalysts were introduced into the COF‐CNT nanohybrids via physisorption, performed by immersing COF‐CNT nanohybrids in the molecular catalyst solution (further details shown in Supporting Information). Figure 5b shows that when CoTPyP is physisorbed into the COF‐CNT nanohybrids (denoted as COF‐CNT:CoTPyP), the resulting cobalt amount is 0.22 wt% for TFB‐Bz‐CNT:CoTPyP, 0.42 wt% for TPB‐MeOTP‐CNT:CoTPyP, and 0.37 wt% for TPB‐Bpy‐CNT:CoTPyP, respectively, as determined by inductively coupled plasma optical emission spectroscopy (ICP‐OES). Due to the low loading of CoTPyP, we were unable to detect the cobalt in COF‐CNT:CoTPyP by X‐ray photoelectron spectroscopy and TEM energy‐dispersive spectroscopy, and no significant decrease in S_BET_ is observed (Figures S31–S33). However, the UV‐vis spectra of COF‐CNT:CoTPyP show the absorption band of CoTPyP. As depicted in Figures 5c and S34, CoTPyP chloroform solution exhibits an absorption peak at 528 nm, attributed to the electronic transitions of the porphyrin Q bands. The appearance of the Q‐band absorption of CoTPyP in the UV‐vis absorption spectrum of COF‐CNTs:CoTPyP suggests the successful incorporation of CoTPyP into COF‐CNT nanohybrids.

Electrochemical CO_2_ reduction performance was evaluated in a gas‐tight H‐type cell under constant CO_2_ flow with 0.5 M KHCO_3_ as electrolyte (pH 7.3). The electrodes were prepared by mixing COF‐CNT nanohybrids with carbon black (Vulcan XC72R) and drop‐casting the mixture on carbon paper electrodes. Carbon black serves as the conducting agent on the electrode, and the effect of the ratio between carbon black and COF‐CNT nanohybrids is discussed below. Cyclic voltammetry (CV) indicates that the incorporation of CoTPyP affords COF‐CNT nanohybrids with an excellent CO_2_ reduction behavior (Figure 5d). TPB‐MeOTP‐CNT:CoTPyP exhibits the lowest onset potential (*V*_onset_) of ‒446 mV vs. the reversible hydrogen electrode (RHE), defined as the potential where the current density reaches 1 mA cm^‒2^. A minor *V*_onset_ shift toward more negative potentials is observed for TPB‐Bpy‐CNT:CoTPyP and TFB‐Bz‐CNT:CoTPyP, giving *V*_onset_ of ‒465 mV and ‒557 mV vs RHE, respectively. Nevertheless, all COF‐CNT:CoTPyP electrodes show significantly enhanced performance compared to the ones based on pure COF‐CNT nanohybrids, as indicated by the *V*_onset_ of ‒772 mV vs RHE from the TPB‐MeOTP‐CNT electrode without introducing CoTPyP. Controlled potential electrolysis was performed under identical conditions to analyze and quantify the electrolysis products. As shown in Figure 5e, the CO evolution Faradaic efficiency (FE(CO)) of TPB‐MeOTP‐CNT is less than 3%, suggesting that the pure COF‐CNT nanohybrids do not possess inherent catalytic activity for electrochemical CO_2_ reduction. In contrast, the FE(CO) of the COF‐CNT:CoTPyP reaches above 97% and 94% at the optimal potentials of ‒0.73 and ‒0.83 V vs RHE, respectively. Given the combination of the larger catalytic current density and the higher FE(CO), the incorporation of CoTPyP increases the CO partial current density by three orders of magnitude (Figure 5f). It is worth mentioning that after 34 h of continuous measurement at ‒0.73 V vs RHE, a FE(CO) of above 90% is maintained, demonstrating an extraordinary stability (Figure 5g). The turnover number (TON), calculated by normalizing the CO product amount with the total Co amount on the electrode, is determined to be 203542 for the 34 h continuous measurement. No liquid product is found in the electrolyte, as evidenced by NMR measurements (Figure S36). ^13^CO_2_ isotope labelling experiments prove that the CO product is converted from CO_2_ (Figure S37). Post‐electrolysis characterizations, including GIWAXS, SEM, and TEM were carried out to confirm the robustness of the COF‐CNT nanohybrids as well as to verify whether there are any Co metal particles generated from the pristine samples [36]. GIWAXS 2D patterns of the electrodes operated under electrochemical CO_2_ reduction conditions indicate that the COF reflections remain after electrolysis and no reflections assigned to metal nanoparticle formation emerge (Figure S38). This observation is consistent with the morphology preservation of the COF‐CNT nanohybrids as well as the absence of metal nanoparticles suggested by SEM and TEM images (Figures S39, S40) even after 15 h continuos operation (Figures S41). These results demonstrate that the COF‐CNT nanohybrids are stable under the electrocatalytic conditions, and metal nanoparticles are not the true catalytically active species. This is different from cases where the in situ transformation of coordinated metal atoms into metal (oxide) nanoparticles provides the true catalytically active species for electrocatalysis [36].

**FIGURE 6:**
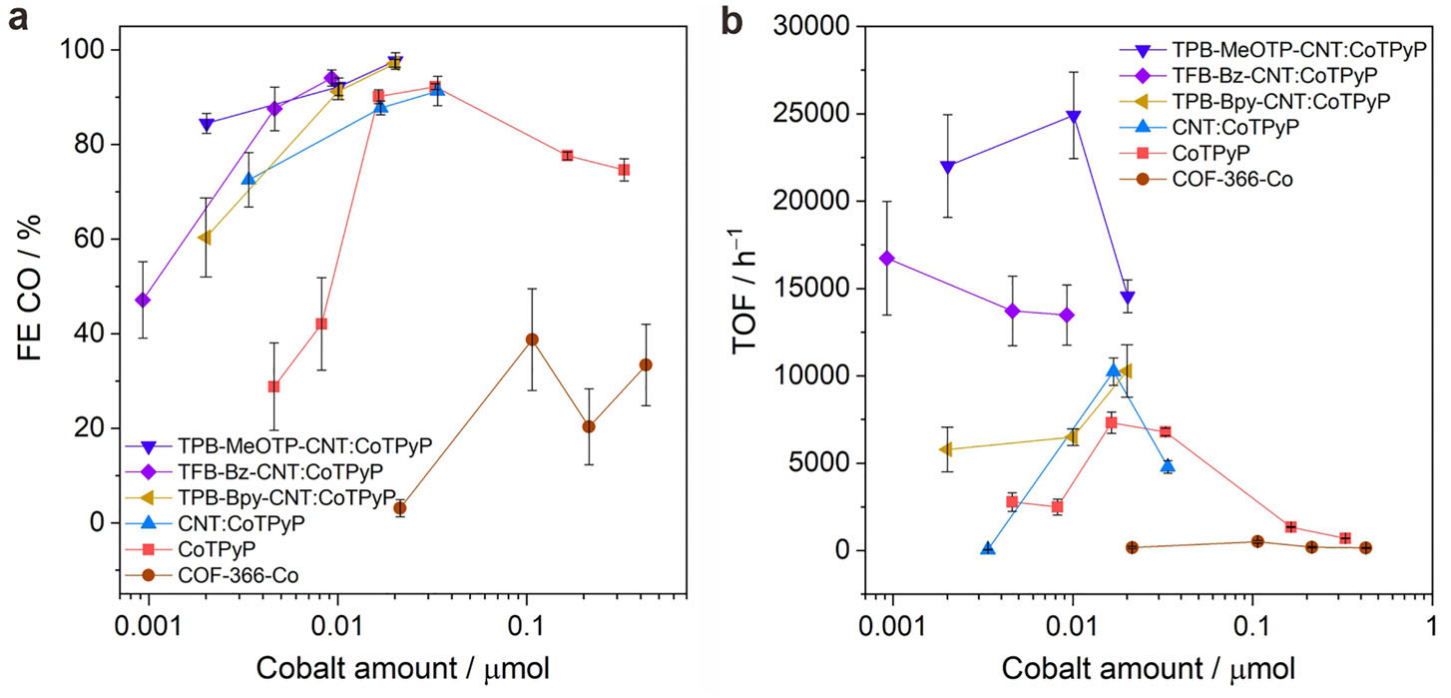
Comparison of electrocatalytic CO_2_ reduction performance. CoTPyP physisorbed COF‐CNT nanohybrids (COF‐CNT:CoTPyP) and other control systems, including CoTPyP, CNT:CoTPyP, and COF‐366‐Co. The cobalt amount on the electrodes was adjusted by varying the mass ratio between carbon black and molecular catalysts. CO production Faradaic efficiencies (a) and CO production turnover frequencies (TOF) at ‒0.83 V vs. RHE (b), respectively. Standard deviations were obtained from four measurements.

To verify the utilization efficiency of the catalytic centers, we investigated the influence of the cobalt amount on the electrode for the electrochemical CO_2_ reduction performance, by varying the ratio between carbon black and various electrocatalysts. As carbon black and carbon paper can produce H_2_ under CO_2_ reduction conditions, we envision that highly dispersed cobalt catalytic centers, combined with favorable electron conducting pathways and mass transport, will result in a significant increase in the utilization of catalytic centers. Therefore, we varied the ratio between carbon black and electrocatalysts while keeping the carbon black amount on the electrode fixed to adjust the cobalt amount on the electrode and to investigate the cobalt utilization efficiency. For this purpose, COF‐CNT:CoTPyP, molecular catalyst CoTPyP, CoTPyP physisorbed on bare CNT (denoted as CNT:CoTPyP), and COF‐366‐Co were studied and compared, and the results are summarized in Figure 6. As shown in Figure 6a, when reducing the cobalt amount on the electrode to <0.016 µmol, the FE(CO) of the CoTPyP electrode decreases rapidly. This is because aggregated CoTPyP particles render the cobalt catalytic centers inaccessible in the interior of the particles (Figure S42), resulting in a more competitive H_2_ evolution from the carbon materials. In comparison, COF‐CNT:CoTPyP electrodes maintain a higher FE(CO) with decreasing the amount of cobalt on the electrodes, and indeed show the highest FE(CO) among all samples, including COF‐366‐Co. Moreover, the TOF results of varying the cobalt amount on these electrodes further demonstrate the enhanced cobalt utilization of COF‐CNT nanohybrids. Figure 6b shows that TPB‐MeOTP‐CNT:CoTPyP exhibits the highest TOF, reaching 24914 h^−1^ with a FE(CO) of 92%, which represents a two‐orders‐of‐magnitude increase in TOF at the cobalt amount range of 0.01 – 0.001 µmol compared to the pristine molecular catalyst CoTPyP and COF‐366‐Co. It is noted that small peaks corresponding to CoTPyP aggregates are observed in the PXRD pattern of TPB‐MeOTP‐CNT:CoTPyP (Figure S43). To rule out the possibility that these aggregates are the primary active species in TPB‐MeOTP‐CNT:CoTPyP, we varied the CoTPyP loading time ranging from 4 h to 30 min (also displayed in Figure S43). This variation effectively eliminates CoTPyP aggregate peaks in the PXRD pattern and is accompanied by a decrease in cobalt amount from 0.5 wt% to 0.04 wt%. Furthermore, the fact that the sample containing CoTPyP aggregates does not exhibit the highest CO production TOF indicates that the CoTPyP aggregates are not the dominant active species. This point was further validated in other molecular catalyst systems, CoPc and CoTPP, where no catalyst aggregate peaks in the PXRD patterns are observed, while the highest TOF reaches 85918 h^−1^ (FE(CO) of 94%) and 17294 h^−1^ (FE(CO) of 65%), respectively, at −0.83 V vs. RHE (Figures S44 and S45). These values place the COF‐CNT‐supported molecular catalysts among the most efficient systems reported to date for electrochemical conversion from CO_2_ to CO (Table S1). It is also worth mentioning that incorporating the molecular catalyst into COF–CNT nanohybrids results in a significantly higher FE(CO) and approximately fivefold higher TOF compared to CoTPyP supported on solvothermally prepared COF. This enhancement highlights the superior role of the COF shell in facilitating more efficient mass transport and improving site accessibility for CoTPyP, owing to its high crystallinity and perpendicularly oriented pore channels (Figures S46).

## 3 Conclusion

In summary, we have demonstrated the synthesis of COF‐CNT core‐shell nanohybrids, designed to achieve COF electrocatalysts with well‐defined morphology and enhanced activity based on a per‐atom (Co) basis. Three COF‐CNT nanohybrids with different lattice sizes, namely TFB‐Bz‐CNT, TPB‐MeOTP‐CNT, and TPB‐Bpy‐CNT were obtained via a colloidal COF synthesis approach, wherein homogeneous COF nanoparticles form in solution, and a COF shell of 50–80 nm thickness is grown on the CNT via direct nucleation. Structural characterizations, including PXRD and N_2_ physisorption, confirm that the COF shells are crystalline and mesoporous. Furthermore, TEM imaging indicates that the COF pores are oriented perpendicularly to the CNT substrate, a finding corroborated by GIWAXS characterization, which reveals the formation of oriented COF films on MLG SiO_2_/Si wafers. The catalytic application of COF‐CNT nanohybrids in electrochemical CO_2_ reduction is demonstrated by incorporating molecular catalysts (CoTPyP, CoPc and CoTPP) into the COF pores via physisorption. The resulting COF‐CNT:CoTPyP electrocatalyst exhibits high selectivity for CO production, achieving a maximum FE(CO) of 97% at −0.73 V vs. RHE. Moreover, we find that COF‐CNT:CoTPyP possesses remarkable stability, maintaining its core‐shell structure and crystallinity after continuous electrochemical operation for 34 h. Notably, the best‐performing TPB‐MeOTP‐CNT:CoTPyP exhibits a two‐order‐of‐magnitude increase in TOF in the cobalt amount range of 0.01 – 0.001 µmol compared to the CoTPyP molecular catalyst and COF‐366‐Co. High per‐atom (Co) activity was also observed with other molecular catalysts, such as CoPc and CoTPP, further demonstrating the ability of COF‐CNT nanohybrids to enhance the utilization efficiency of molecular catalytic sites, likely through confinement‐induced deaggregation and funneling of the molecular catalysts to the CNT current collector. Further enhancement of the intrinsic conductivity of the COF shell, for example through the integration of conductive or redox‐active COF motifs, represents a promising strategy to improve charge transport while preserving the spatial isolation and accessibility of molecular catalytic sites. Moreover, owing to the colloidal and solution‐processable nature of the synthesis, the COF–CNT nanohybrid platform is inherently compatible with scale‐up under continuous‐flow reaction conditions. Therefore, this study not only provides a robust strategy for synthesizing well‐defined COF‐mediated electrocatalysts but also highlights the potential of COF‐CNT nanohybrids as a versatile platform for enhancing molecular catalyst utilization, paving the way for future advancements in COF‐based electrochemical energy conversion.

## Conflicts of Interest

The authors declare no conflicts of interest.

## Supporting information




**Supporting File 1**: The authors have cited additional references within the Supporting Information [1–5].

## Data Availability

The data that support the findings of this study are available from the corresponding author upon reasonable request.
