# Supplementary material for: Covalent Organic Framework–Carbon Nanotube Core–Shell Nanohybrids for Enhanced Catalytic Site Utilization of Molecular Catalysts in CO2 Electroreduction
Source: Angew Chem Int Ed Engl. 2026 Mar 19;65(18):e21776. doi: 10.1002/anie.202521776 (PMC13110783; doi:10.1002/anie.202521776)
Supplement: Supplementary file 1 — Supporting File 1: The authors have cited additional references within the Supporting Information [1–5]. [file ANIE-65-e21776-s001.docx]

Covalent Organic Framework–Carbon Nanotube Core–Shell Nanohybrids for Enhanced Catalytic Site Utilization of Molecular Catalysts in CO_2_ Electroreduction

*Liang Yao^2†*^, Andrés Rodríguez-Camargo^1,4†^, Roman Guntermann^3^, Fabian Heck^1,5^, Samuel Van Gele^1,5^, Hugo Vignolo-González^1,5^, Viola Duppel^1^, Thomas Bein^3,6^, Bettina V. Lotsch^1,4,5,6*^*

^1^Nanochemistry Department, Max Planck Institute for Solid State Research, Heisenbergstraße 1, 70569 Stuttgart, Germany

E-mail: [b.lotsch@fkf.mpg.de](mailto:b.lotsch@fkf.mpg.de)

^2^State Key Laboratory of Luminescent Materials and Devices, Institute of Polymer Optoelectronic Materials and Devices, Guangdong Basic Research Center of Excellence for Energy and Information Polymer Materials, Guangdong Provincial Key Laboratory of Luminescence from Molecular Aggregates, South China University of Technology, Guangzhou 510640, China

Email: [liangyao@scut.edu.cn](mailto:liangyao@scut.edu.cn)

^3^Department of Chemistry and Center for NanoScience (CeNS), Ludwig-Maximilians-Universität München, Butenandtstraße 5-13 (E), 81377 Munich, Germany

^4^Department of Chemistry, University of Stuttgart, Pfaffenwaldring 55, 70569 Stuttgart, Germany

^5^Department of Chemistry, University of Munich, Butenandtstraße 5-13, 81377 Munich, Germany

^6^E-conversion and Center for Nanoscience, Lichtenbergstraße 4a, 85748 Garching bei München, Germany

^†^These authors contributed equally to this work.

**1. Experimental methods**

**Chemicals**

1,3,5-Triformylbenzene (TCI, >98.0%), Benzidine (abcr GmbH, 98%), 2,5-dimethoxyterephthalaldehyde (MeOTP, Sigma-Aldrich, 97%), 2,2'-Bipyridyl-5,5'-dialdehyde (abcr GmbH, 98%), 1,3,5-tris(4-aminophenyl)benzene (TAPB, TCI, >93%), acetonitrile (Carl Roth, ≥99.5%), mesitylene (TCI, >97.0%), dioxane (Acros Organics, 99.5% with a sealing cap), acetic acid (AcOH, Carl-Roth, 100%), multi-walled carbon nanotube (Sigma-Aldrich, 50-90 nm diameter, >95% carbon basis), carbon nanotube (Sigma-Aldrich, >98% carbon basis, O.D. × L 6-13 nm × 2.5-20 μm), Nafion 117 solution (Sigma-Aldrich, 5% in a mixture of lower aliphatic alcohols and water), 5,10,15,20-tetra(4-pyridyl)porphyrin (H_2_TPyP, Sigma-Aldrich, 97%), meso-tetraphenylporphyrin (H_2_TPP, Sigma-Aldrich, 97%), Cobalt(II) acetate tetrahydrate (Co(OAc)_2_·4H_2_O, Alfa Aesar, 98%), CoPc (Sigma-Aldrich, β-form, 97%), monolayer graphene (MLG) on Si/SiO_2_ wafer (Sigma-Aldrich).

**Characterizations**

**Atomic force microscopy.** Atomic force microscopy (AFM) was performed using Asylum Research MFP-3D, equipped with a micro cantilever (OMCL-AC160TS-R3). TPB-MeOTP, TFB-Bz and TPB-Bpy films were grown on MLG/SiO_2_ substrate as described in the synthesis part.

**Scanning electron microscopy.** SEM images were measured with a Zeiss Merlin under the electron high tension voltage of 1.5 kV. 3 nm of Ir was sputtered on the samples by Leica ACE600.

**FT-IR spectroscopy.** FT-IR measurements were carried out on a PerkinElmer Spectrum Two in attenuated total reflection (ATR) geometry equipped with a diamond crystal.

**Super critical CO_2_ activation.** Super critical CO_2_ activation was performed on a Leica EM CPD300 critical point dryer. Prior to super critical CO_2_ activation, the COF samples were soaked in methanol.

**Sorption Measurements.** N_2_ sorption were measured with a Quantachrome Instruments Autosorb iQ 3 at 77 K. The pore size distribution (PSD) was determined with the calculation model of N_2_ at 77 K on carbon using the QSDFT (cylindrical pores, adsorption branch) in ASiQwin software v 3.01. The COF samples were activated in high vacuum for 12 h before measurement. A suitable pressure region (P/P_0_ = 0.05−0.2) was chosen for the BET surface area determination.

**Grazing-incidence wide angle X-ray scattering.** Two-dimensional grazing-incidence wide angle X-ray scattering (GIWAXS) data were recorded with an Anton Paar SAXSpoint 2.0 system equipped with a Primux 100 micro Cu K_α_ source and a Dectris EIGER R 1M 2D detector. The COF films were positioned at a sample-detector distance of 140 mm and were measured with an incidence angle of 0.2°.

**UV-vis spectroscopy.** The UV-Vis absorption spectra of CoTPyP and CoTPP solution were recorded in an Agilent Technologies Cary 60 spectrometer using a quartz cuvette (1 cm path length). UV-Vis absorption spectra of COF-CNT nanohybrid solutions were collected on a Cary 5000 spectrometer equipped with an integrating sphere (referenced to barium sulphate).

**Powder X-ray diffraction.** Powder X-ray diffraction (PXRD) patterns were recorded in Debye-Scherrer geometry at room temperature, on a Stoe Stadi P diffractometer (Cu-K_α1_ or Co-K_α1_) equipped with a Ge(111) primary monochromator. All the samples were sealed in 1.0 mm glass capillaries, and measured with spinning for improving particle statistics.

**Nuclear magnetic resonance.** ^1^H-NMR spectra of the electrolyte were recorded on a JEOL ECZ 400S 400 MHz spectrometer.

**ICP-OES.** ICP-OES measurements for cobalt amount determination were performed on a Vista Pro simultaneous ICP-OES spectrometer (Agilent Technologies, Santa Clara, California, USA) with a CCD-detector. Samples were treated by microwave digestion with Discover SP-D from CEM GmbH (sample dissolved in HNO_3_ (65%) at 185 °C for 25 min./diluted with double distilled water). The results were analyzed with Software ICP-Expert.

**Transmission Electron Microscopy.** TEM images were obtained with a Philips CM 30 ST microscope (300 kV, LaB6 cathode), equipped with a TVIPS TemCam-F216 CMOS Camera. The program EM-Menu 4.0 Extended was used to perform Fast Fourier Transforms (FFTs). The COF samples were distributed onto a holey carbon/copper grid prior to the measurements. Scanning TEM (STEM) were performed at a JEOL ARM 200CF scanning transmission electron microscope equipped with a cold field emission electron source, a DCOR probe corrector (CEOS GmbH) and a 100 mm^2^ JEOL Centurio EDX detector. Pathfinder Software from Thermo Fisher Scientific was used for EDX data analysis.

**Synthesis**

**Colloidal COF synthesis.**

**TPB-MeOTP colloid:** The synthesis is based on a modified polar co-solvent approach developed by Ditchtel et al.^1^ In general, 2,5-dimethoxyterephthalaldehyde (20.8 mg, 59.2 μmol) was firstly dissolved in a mixture of acetonitrile, mesitylene and dioxane (14 mL in total) in a Biotage 20 mL high precision glass vial, and TAPB (17.2 mg, 88.6 μmol) was then added into the solution. The optimization of reaction solvent was shown in Figure S1, where the ratio between mesitylene and dioxane was fixed to 4:1, and the total volume of the mesitylene and dioxane in the reaction solvent system was varied from 0%, 25%, 50%, 75% to 100%. The vial was sealed with a cap and stirred at room temperature. 1 mL 12 M acetic acid was injected into the solution within 5 min. Then the reaction solution was heated to 90 °C and slowly stirred at 300 rpm for 20 h. After the reaction, the reaction vial was cooled down to room temperature. To collect the particle powder for PXRD, TEM, BET, and FT-IR measurements, 1 M NaCl aqueous solution (200 μL) was added in the vial to precipitate out the particles. The precipitates were collected with a filter paper, subjected to Soxhlet extraction with chloroform and methanol for 12 h each. The final product was obtained after activating with supercritical CO_2_. In the whole workup process, the precipitates remained wet prior to the super critical CO_2_ activation.

**TPB-Bpy colloid:** Following the synthesis procedure of TPB-MeOTP colloid, 2,2'-bipyridyl-5,5'-dialdehyde (18.8 mg, 88.6 μmol), TAPB (20.8 mg, 59.2 μmol), acetic acid (1 mL, 12 M), and acetonitrile/mesitylene/dioxane solvent mixture (14 mL in total) were used for the synthesis. The ratio between mesitylene and dioxane was fixed to 4:1, and the volume amount of the mesitylene and dioxane in the reaction solvent system was varied from 0%, 25%, 50%, 75% to 100%.

**TFB-Bz colloid:** Following the synthesis procedure of TPB-MeOTP colloid, benzidine (16.4 mg, 88.6 μmol), 1,3,5-triformylbenzene (9.6 mg, 59.2 μmol), acetic acid (1 mL, 12 M), and acetonitrile/mesitylene/dioxane solvent mixture (14 mL in total) were used for the synthesis. The ratio between mesitylene and dioxane was fixed to 4:1, and the volume amount of the mesitylene and dioxane in the reaction solvent system was 0% or 50%.

**COF-CNT nanohybrid composite synthesis**

**TPB-MeOTP-CNT:** In general, the synthesis of COF-CNT nanohybrids was followed with synthesis procedure of aforementioned COF colloids. Firstly, 2,5-dimethoxyterephthalaldehyde (20.8 mg, 59.2 μmol) firstly dissolved in a mixture of acetonitrile, mesitylene and dioxane (7 mL, 5.6 mL and 1.4 mL, respectively) in a Biotage 20 mL high precision glass vial, and TAPB (17.2 mg, 88.6 μmol) and CNT (15 mg) was then added into the solution. The vial was sealed with a cap and stirred at room temperature. 1 mL 12 M acetic acid was injected into the solution within 5 min. Then the reaction solution was heated to 90 °C and slowly stirred at 300 rpm for 20 h. After the reaction, the reaction vial was cooled down to room temperature. A plastic pipette was used to remove the COF colloid solution. 10 mL fresh acetonitrile was added for washing the residual COF colloids, which was repeated by three times. The obtained COF-CNT nanohybrids were subjected to Soxhlet extraction with chloroform and methanol for 12 h each, and activated with supercritical CO_2_, providing 29.9 mg of TPB-MeOTP-CNT.

**TPB-Bpy-CNT:** Following the synthesis procedure of TPB-MeOTP-CNT, 2,2'-bipyridyl-5,5'-dialdehyde (18.8 mg, 88.6 μmol), TAPB (20.8 mg, 59.2 μmol), acetic acid (1 mL, 12 M), acetonitrile/mesitylene/dioxane solvent mixture (7 mL, 5.6 mL and 1.4 mL, respectively), and CNT (15 mg) were used for the synthesis, resulting in 34.2 mg of TPB-Bpy-CNT.

**TFB-Bz-CNT:** Following the synthesis procedure of TPB-MeOTP-CNT, benzidine (16.4 mg, 88.6 μmol), 1,3,5-triformylbenzene (9.6 mg, 59.2 μmol), acetic acid (1 mL, 12 M), acetonitrile/mesitylene/dioxane solvent mixture (7 mL, 5.6 mL and 1.4 mL, respectively), and CNT (15 mg) were used for the synthesis, resulting in 26.4 mg of TPB-Bpy-CNT.

**COF film growth on MLG SiO_2_/Si wafer or bare SiO_2_/Si wafer.** The film synthesis procedure is identical to that of the COF-CNT nanocomposites, except that the SiO_2_/Si wafer was added to the reaction solution, instead of CNTs. After the reaction, the COF film was immersed in pure acetonitrile, cleaned via sonication and dried with air blow gun.

**CoTPyP:** CoTPyP was synthesized via a modified reported procedure.^2^ H_2_TPyP (250 mg, 404 mmol), Cobalt(II) acetate tetrahydrate (201 mg, 0.808 mmol) were dissolved in 15 mL acetic acid and 15 mL DMF. The reaction system was purged with Ar for 10 min and then heated at 120 °C for 15 h. After that, the reaction was cooled to room temperature and poured into 40 mL ice/water mixture. The product was filtered and washed with water. The reaction yield was 90.1% (246 mg). Mass spectrum (Calc. m/z: 676.2; Exp. m/z: 676.2).

**CoTPP:** H_2_TPP (200 mg, 0.325 mmol), Cobalt(II) acetate tetrahydrate (162 mg, 0.65 mmol) were dissolved in 12 mL DMF. The reaction system was purged with Ar for 10 min and heated at 120 °C for 15 h. After the reaction, the solution was poured into 40 mL ice/water mixture. The product was filtered and washed with water. The reaction yield was 81.5% (178 mg). Mass spectrum (Calc. m/z: 671.2; Exp. m/z: 671.3).

**Loading molecular catalyst in COFs:**

**COF-CNTs:CoTPyP, CNT:CoTPyP and TPB-MeOTP:CoTPyP**

CoTPyP (1.5 mg) was first dissolved in chloroform (15 mL) in a Pyrex tube at 65 °C. Following complete dissolution, COF-CNTs (5 mg) were added to the solution. The tube was then sealed and the mixture was heated at 60 °C for 4 hours. After that, the COF-CNTs:CoTPyP was collected by filtration, washed with warm chloroform and subsequently dried with supercritical CO_2_. CNT:CoTPyP and TPB-MeOTP:CoTPyP were prepared by the same procedure.

**TPB-MeOTP-CNT:CoTPP**

Similar to the loading of CoTPyP, CoTPP (6.5 mg) was first dissolved in chloroform (3.25 mL) at room temperature given the good solubility of CoTPP. After that, the mixture was stirred for 2 hours. TPB-MeOTP-CNT:CoTPP was collected by filtration, washed with 2 mL chloroform, and subsequently dried with supercritical CO_2_.

**TPB-MeOTP-CNT:CoPc**

CoPc (1.5 mg) was first dissolved in DMF (15 mL) at 90 °C, and the solution was filtered with a PTFE filter (130 μm). After that, TPB-MeOTP-CNT was added to the solution. The mixture was heated at 70 °C for 4 hours. TPB-MeOTP-CNT:CoPc was collected by filtration, washed with warm chloroform and subsequently dried with supercritical CO_2_.

**Electrochemical CO_2_ reduction**

**Electrode preparation.** The catalyst inks were prepared by mixing COF catalysts (or other control catalysts), carbon black (Vulcan XC 72R), and Nafion solution (5%, 20 µL per mg of COF) in anhydrous ethanol (500 µL per mg of COF), followed by high-speed magnetic stirring overnight. The carbon black amount was fixed at 4 mg/mL in the solution, while the catalyst amount was varied as shown in the Figure 5 of the main text. Polytetrafluoroethylene (PTFE)-coated carbon paper (5%, Quintech TP-060-T5) was cut into a T shape, with a catalyst-coating area of ca. 0.65 cm × 1 cm and a non-coating area of ca. 0.4 cm × 1 cm. The prepared carbon paper pieces were cleaned by sonicating in ethanol for 10 minutes twice, and then dried using compressed air. The catalyst ink was sonicated again before being drop-casted (30 µL per side, with a COF loading of 0.12 mg) onto the catalyst-coating areas of both sides of the carbon paper. The drop-casted pieces were then dried under vacuum and connected to copper wires by sandwiching the non-coating areas and copper wires between titanium foils, using a custom-made polyetheretherketone (PEEK) electrode holder.

**Electrochemistry setup.** Electrochemical measurements and electrolysis were performed using a Pine Research WaveDriver 200 EIS Bipotentiostat and a custom-designed, gas-tight, two-compartment H-cell. The H-cell was optimized for volume, inertness, gas-tight connections, compatibility with high-purity gas handling. An anion exchange membrane (Fumatech fumasep FAB-PK-130) was pre-treated sequentially with 0.5 M NaCl solution, 0.5 M KOH solution, and 0.5 M KHCO_3_ solution before being assembled inside the H-cell. A three-electrode configuration was employed, with a Ag/AgCl reference electrode (porous glass/sat. KCl/AgCl/Ag) and a Pt foil counter electrode. The working compartment of the H-cell was fitted with a reference electrode, a working electrode, a stir bar, a CO_2_ inlet tube, and a gas outlet tube connected with a GC for gas product analysis, while the counter compartment contained the counter electrode and a leak valve. Both compartments were filled with 0.5 M KHCO_3_ electrolyte (8.5 mL in the working compartment and 6 mL in the counter compartment). The working compartment was continuously bubbled with CO_2_ at a flow rate of 19.2 mL/min (normalized to 0 °C), controlled by a Bronkhorst High-Tech F-201DV mass flow controller, under magnetic stirring and regulated by a back-pressure controller set at 1150 mbar. Before conducting any electrochemical experiments, the electrolyte was bubbled with CO_2_ for 10 minutes to saturate the electrolyte with CO_2_ and purge oxygen out from the system. The working electrodes were activated by performing cathodic scan cycles (0 V to -0.83 V vs RHE, 20 mV/s, 10 cycles). The electrolyte pH was 7.3 after being saturated with CO_2_. All experiments were carried out at ambient temperature. Cyclic voltammograms were recorded with a scan rate of 5 mV/s. For ^13^CO_2_ experiments, the electrolyte was bubbled with ^13^CO_2_ in KHCO_3_.

Electrode potentials were converted to the reversible hydrogen electrode (RHE) scale using *E*_RHE_ = *E*_Ag/AgCl_ + 0.059 × pH + 0.197 without *iR* compensation. To check the influence of *iR* drop, the uncompensated resistance (R_u_) was measured using electrochemical impedance spectroscopy (EIS) with an electronically equivalent circuit containing R_u_ in a series to a parallel set of a constant phase element and a resistor. Fitting of curves at -0.63 V and -0.83 V vs RHE both showed R_u_ = 4.5 Ω. Since the *iR* drop (< 0.04 V) is minor compared to the potential range applied in electrolyses, *iR* compensation was not performed for our measurements.

**Product analysis.**

**Gas product quantification:** The outflow gas from the H-cell was introduced into a Shimadzu Nexus GC-2030 gas chromatograph (GC) equipped with an autosampler by intervals of 11.2 min, chromatographed with helium (99.999%, Air Liquide) as a carrier gas, and was analyzed with a dielectric-barrier discharge ionization detector (BID). The GC was calibrated with standard calibration gas mixtures containing CO, H_2_, and CH_4_ purchased from Air Liquide (4-point linear calibration, *R*^2^ > 0.999). For ^13^CO_2_ experiments, outflow gas was additionally analyzed by a Shimadzu GCMS-QP2020 gas chromatograph–mass spectrometer by intervals of 15 min.

**Liquid product detection:** A portion of the electrolyte (330 μL) after electrolysis was mixed with D_2_O (250 μL) containing DMSO as an internal standard (20 μL, 25 mM in H_2_O) and subjected to solution ^1^H NMR measurement. No signal of formate or other CO_2_ reduction products was observed.

**Faradaic efficiency calculation:** The Faradaic efficiency (FE) of the products was calculated with the following equation:

$$FE=\frac{PV}{RT}\frac{xnF}{I}$$

Where 𝑃 is the pressure of the headspace controlled by the back pressure controller, 𝑉 is the volumetric gas flow rate controlled by the mass flow controller (19.2 mL min^−1^), 𝑅 is the ideal gas constant, 𝑇 is the temperature in K, *x* is the mole fraction of the product in the outflow gas determined by the GC, *n* is the number of electrons transferred (2 for CO and H_2_), 𝐹 is the Faraday constant, and 𝐼 is the total current measured by the potentiostat.

**Turnover frequency and turnover number calculation:** Turnover frequency (TOF) and turnover number (TON) were calculated by the following equations:

$$TOF=\frac{xPV}{RT}\frac{m_{Co}}{m_{cata}\times\omega_{Co}}$$

$$TON=\sum TOF\Delta t$$

Where 𝑃, 𝑉, 𝑅, 𝑇, and *x* are the same with the variables for FE calculations, *m*_Co_ is the atomic mass of cobalt (58.9 g mol^−1^), *m*_cata_ is the mass of the catalyst on an electrode, and ω_Co_ is the mass fraction of cobalt in the catalyst obtained by ICP-OES measurements.

**2. Supplementary Figures**


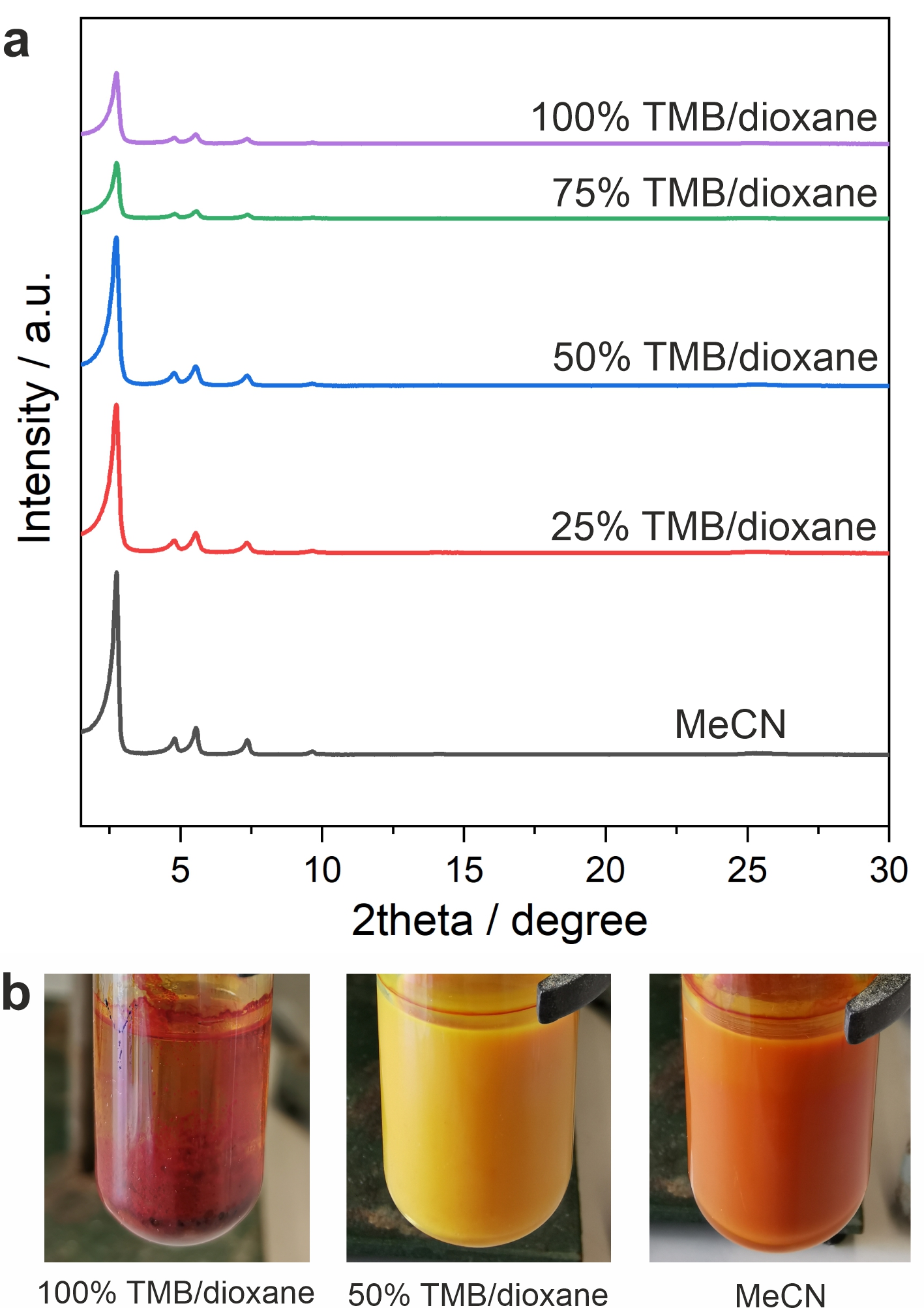


**Supplementary Figure S1.** Solvent optimization for the synthesis of colloidal TPB-MeOTP COF by varying the amount of TMB/dioxane (4:1 volume ratio) in MeCN. TMB: mesitylene; MeCN: acetonitrile. (a) PXRD patterns (Cu-K_α1_); (b) Photographs of the TPB-MeOTP COF products corresponding to different solvent mixtures. The results indicate that a solvent mixture of 50% TMB/dioxane in MeCN produces crystalline colloidal TPB-MeOTP COF.


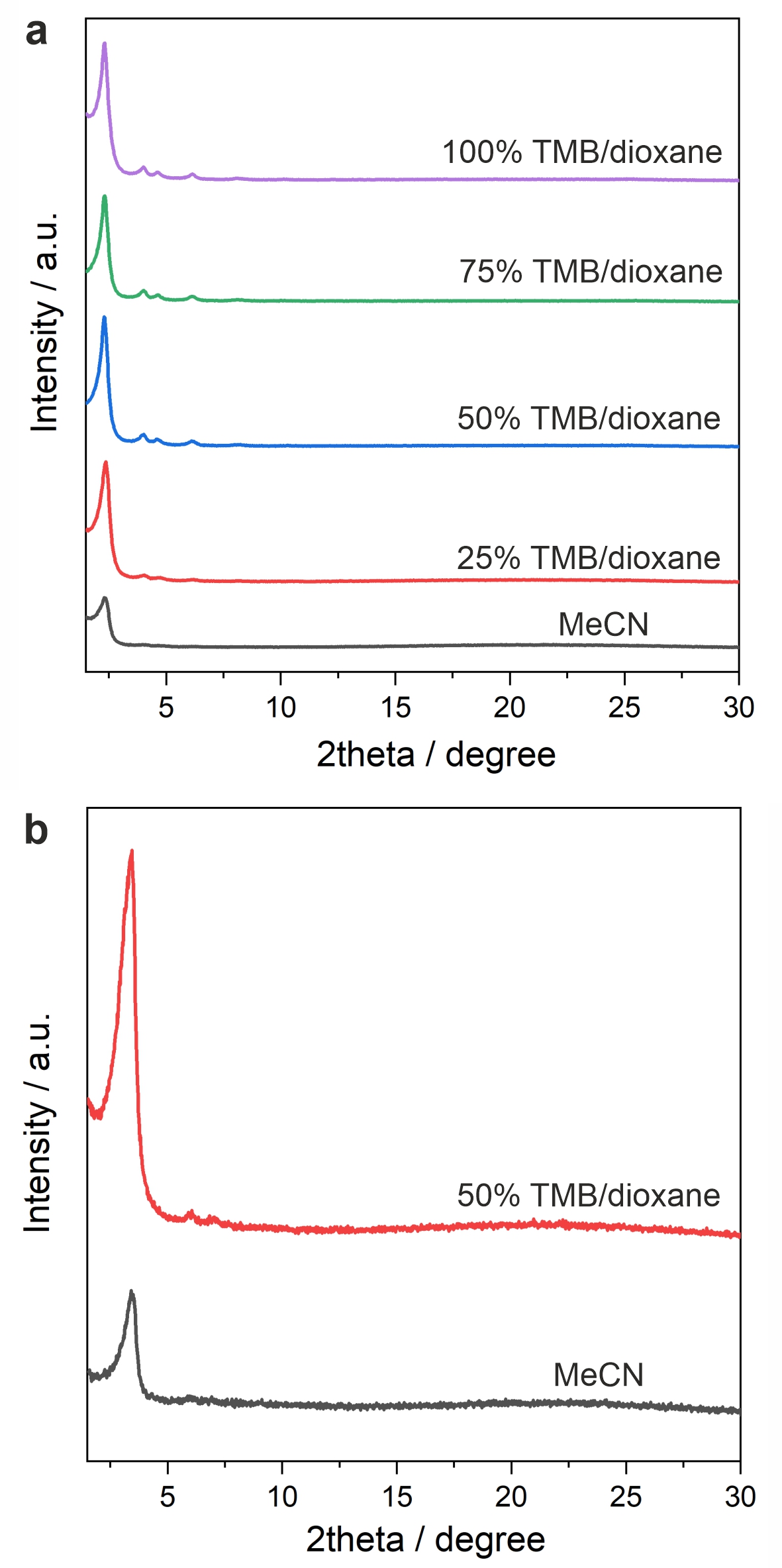


**Supplementary Figure S2.** Solvent optimization for the synthesis of colloidal TPB-Bpy and TFB-Bz COF by varying the amount of TMB/dioxane (4:1 volume ratio) in MeCN. TMB: mesitylene; MeCN: acetonitrile. (a) and (b) show the PXRD patterns (Cu-K_α1_) of colloidal TPB-Bpy and TFB-Bz COF, respectively.


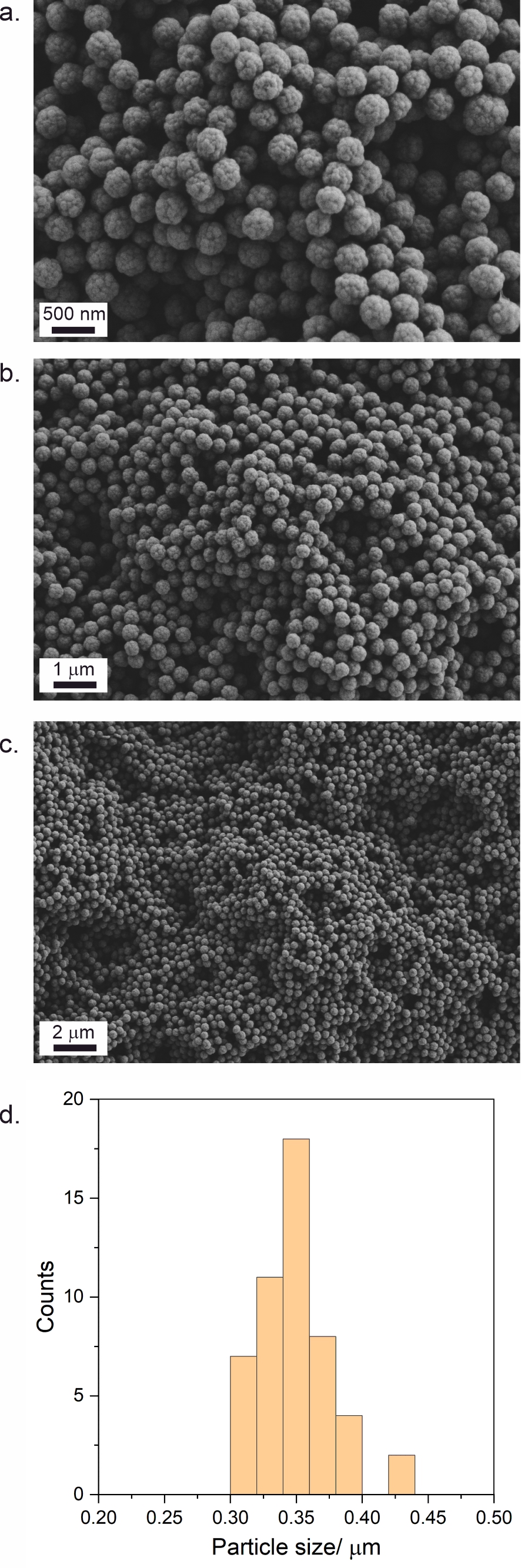


**Supplementary Figure S3.** (a-c) SEM images of colloidal TPB-MeOTP COF obtained with 50% TMB/dioxane (4:1 volume ratio) in MeCN as the reaction solvent. (d) Particle size distribution analysis of 50 particles, giving an average particle size of 350 nm.


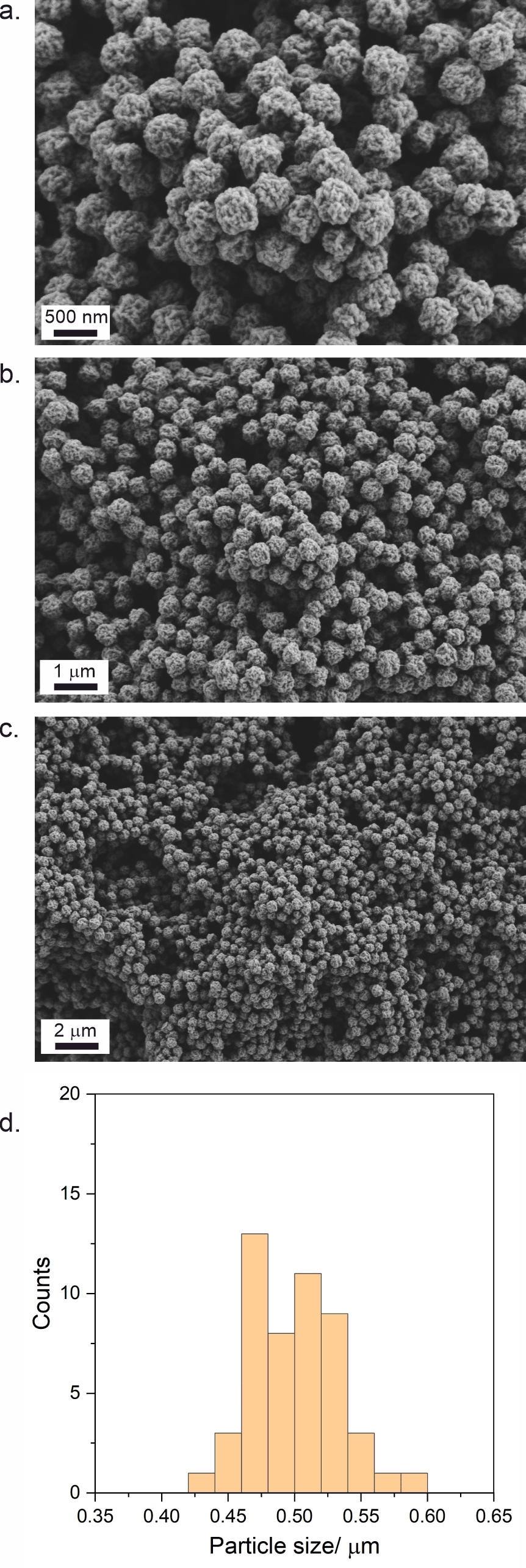


**Supplementary Figure S4.** (a-c) SEM images of colloidal TPB-Bpy COF obtained with 50% TMB/dioxane (4:1 volume ratio) in MeCN as the reaction solvent. (d) Particle size distribution analysis of 50 particles, giving an average particle size of 500 nm.


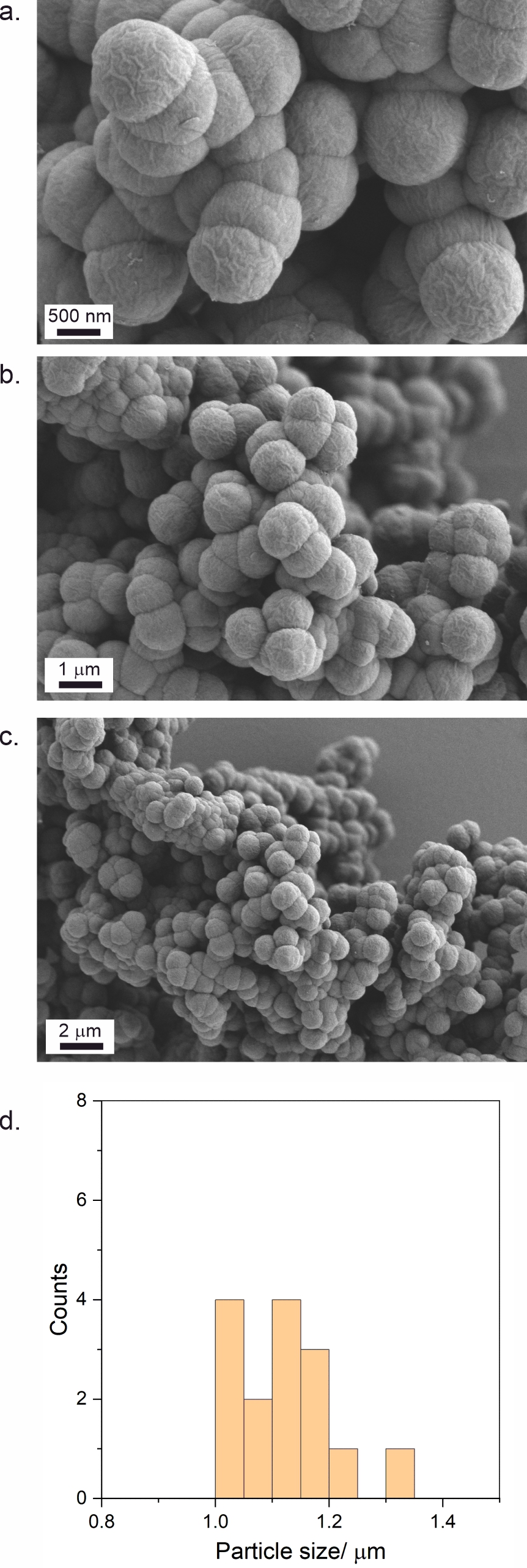


**Supplementary Figure S5.** (a-c) SEM images of colloidal TFB-Bz COF obtained with 50% TMB/dioxane in MeCN as the reaction solvent. (d) Particle size distribution analysis of 15 particles, giving an average particle size of 1.1 μm.


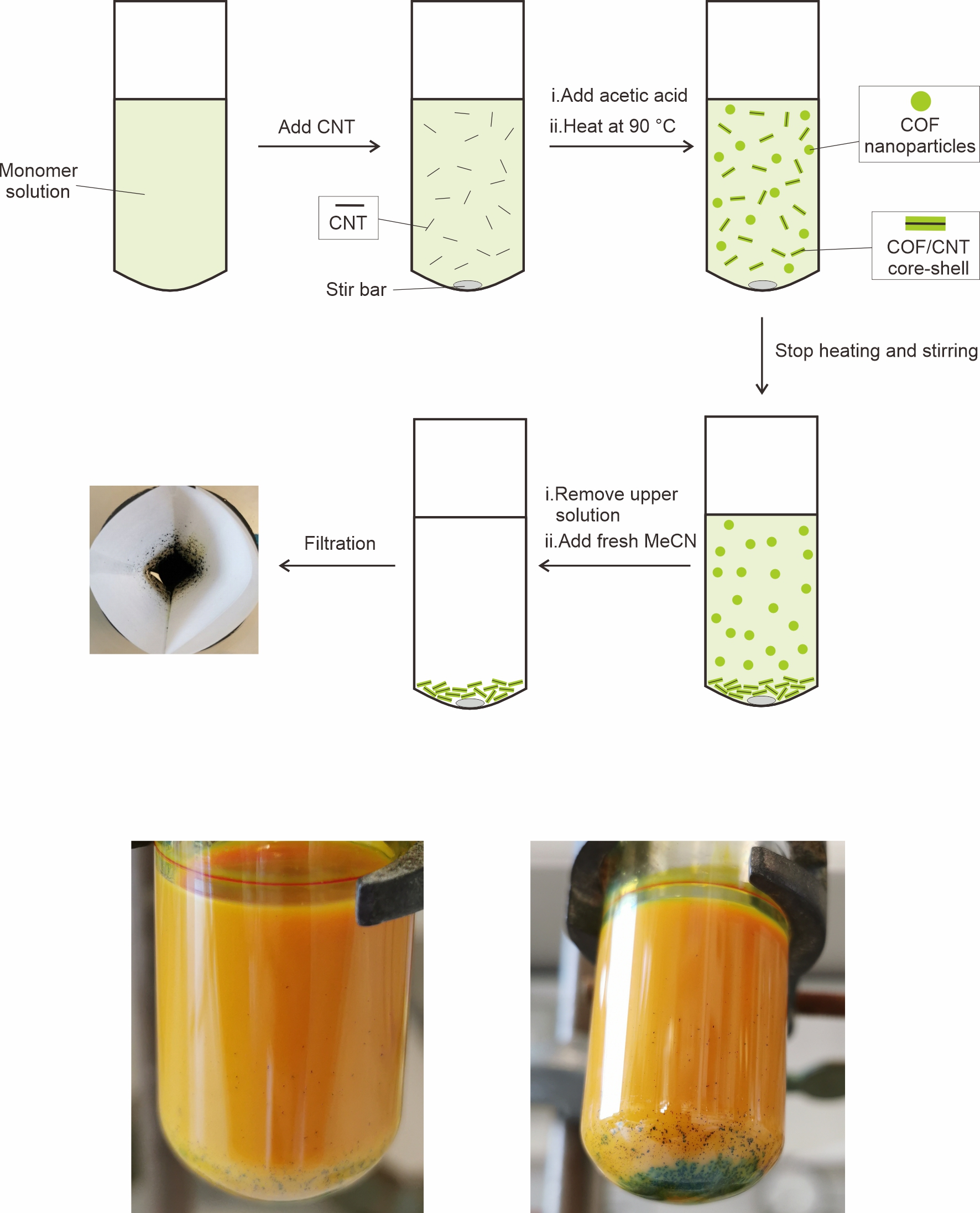


**Supplementary Figure S6.** Schematic illustration of COF-CNT nanohybrid synthesis.


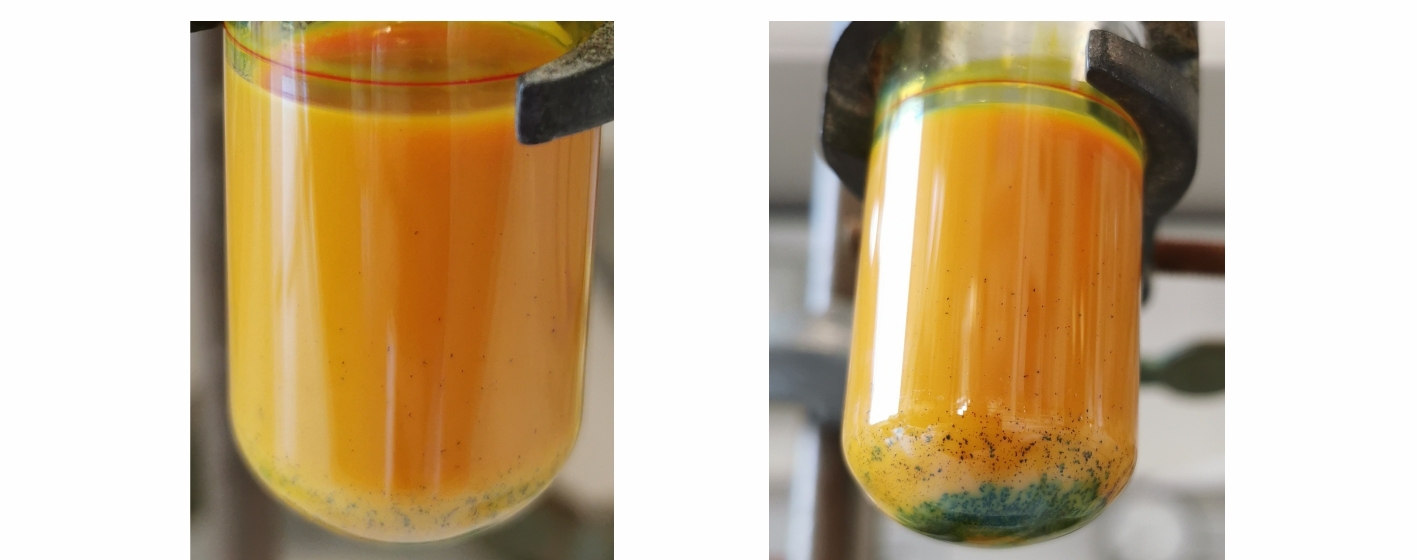


**Supplementary Figure S7.** Photographs of the as-prepared TPB-MeOTP-CNT product. Colloidal TPB-MeOTP particles are suspended in the solution, and the precipitation at the bottom is TPB-MeOTP-CNT.


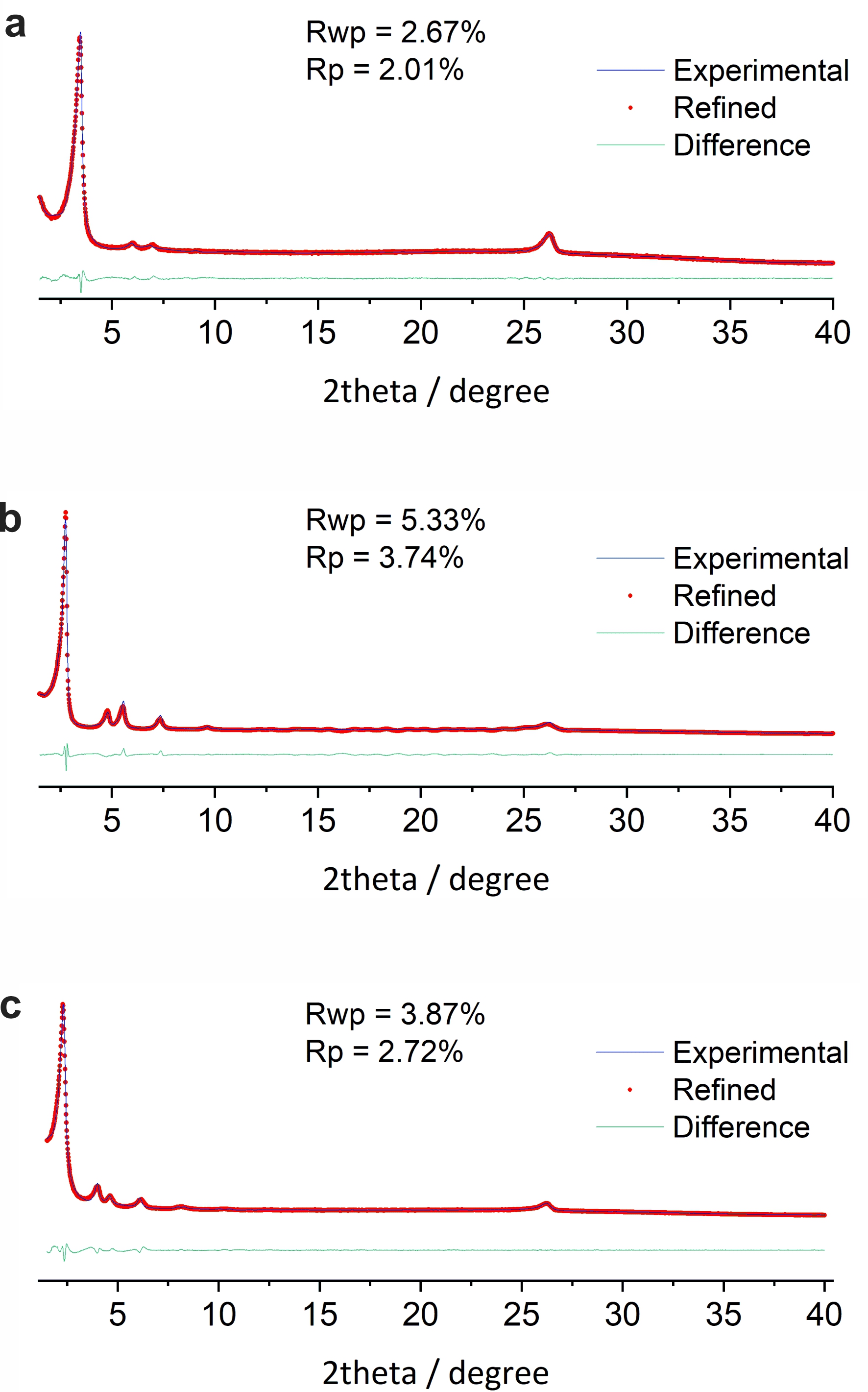


**Supplementary Figure S8.** PXRD profiles (Cu-K_α1_) of TFB-Bz-CNT (a), TPB-MeOTP-CNT (b) and TPB-Bpy-CNT (c). Experimental observed (blue) and Pawley refined (red) PXRD with AA stacking mode.


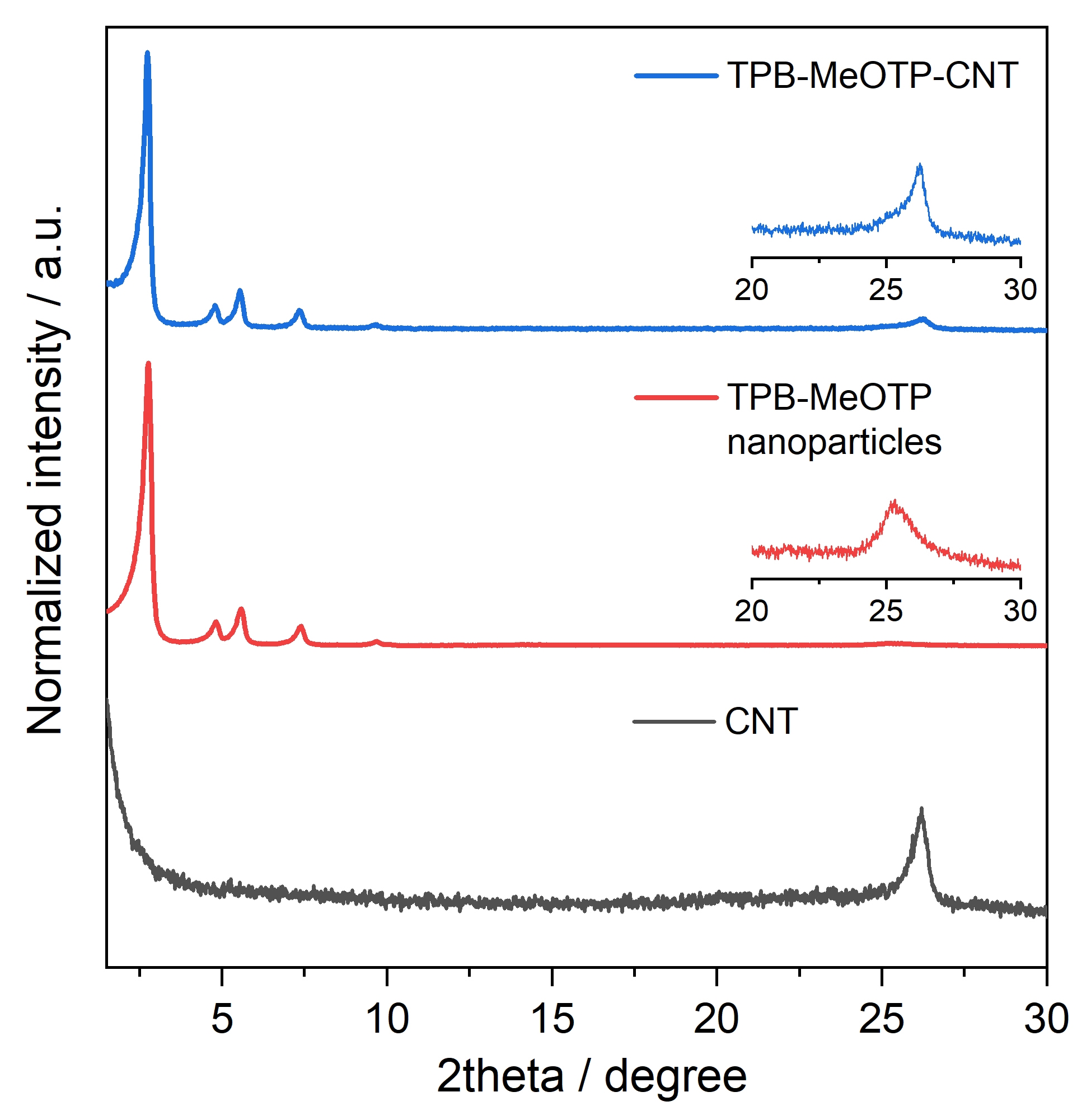


**Supplementary Figure S9.** PXRD patterns (Cu-K*_α_*_1_) of TPB-MeOTP-CNT nanohybrid, TPB-MeOTP nanoparticles and CNT.


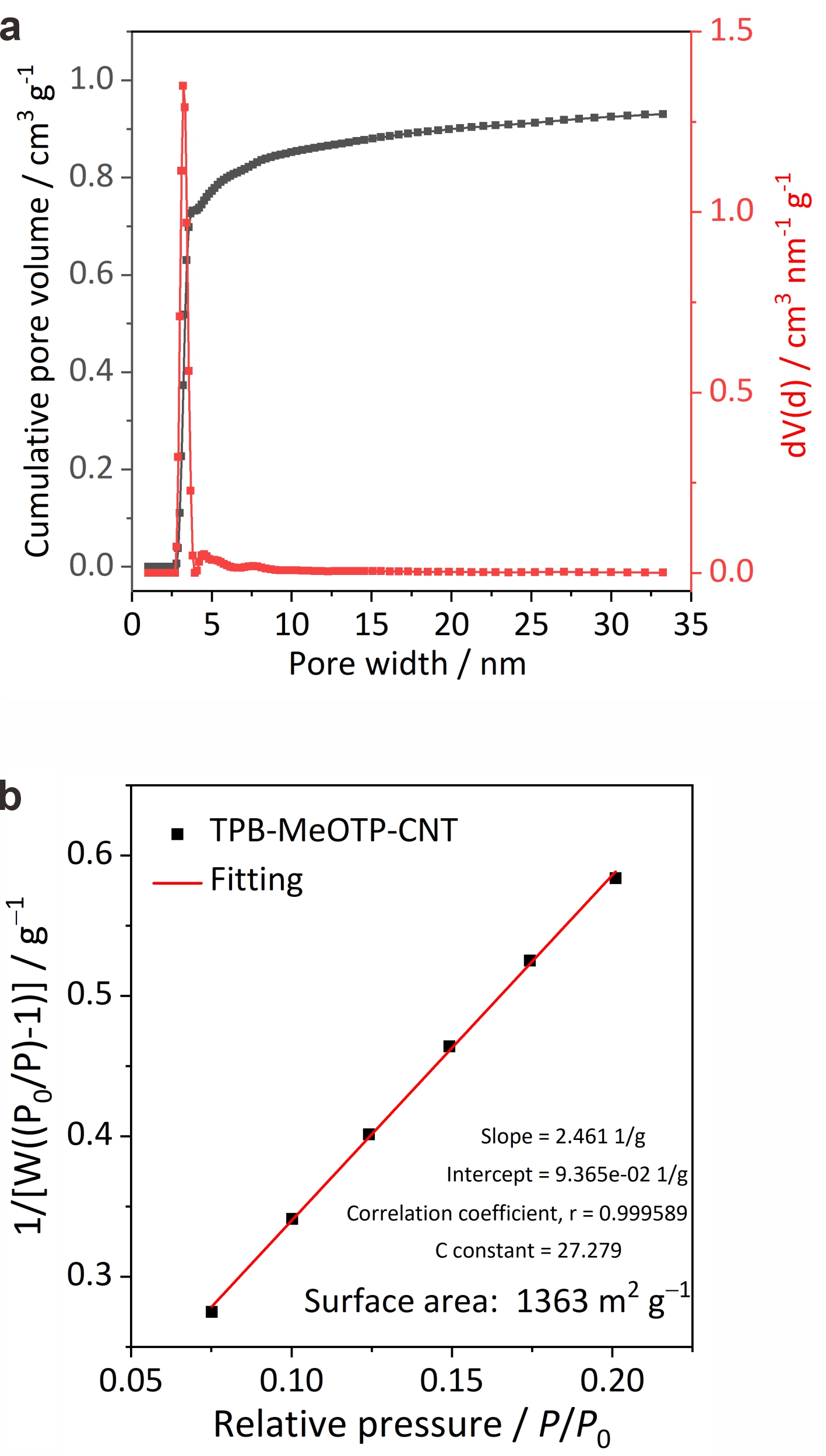


**Supplementary Figure S10.** Pore-size distribution (a) and BET plot (b) of TPB-MeOTP-CNT.


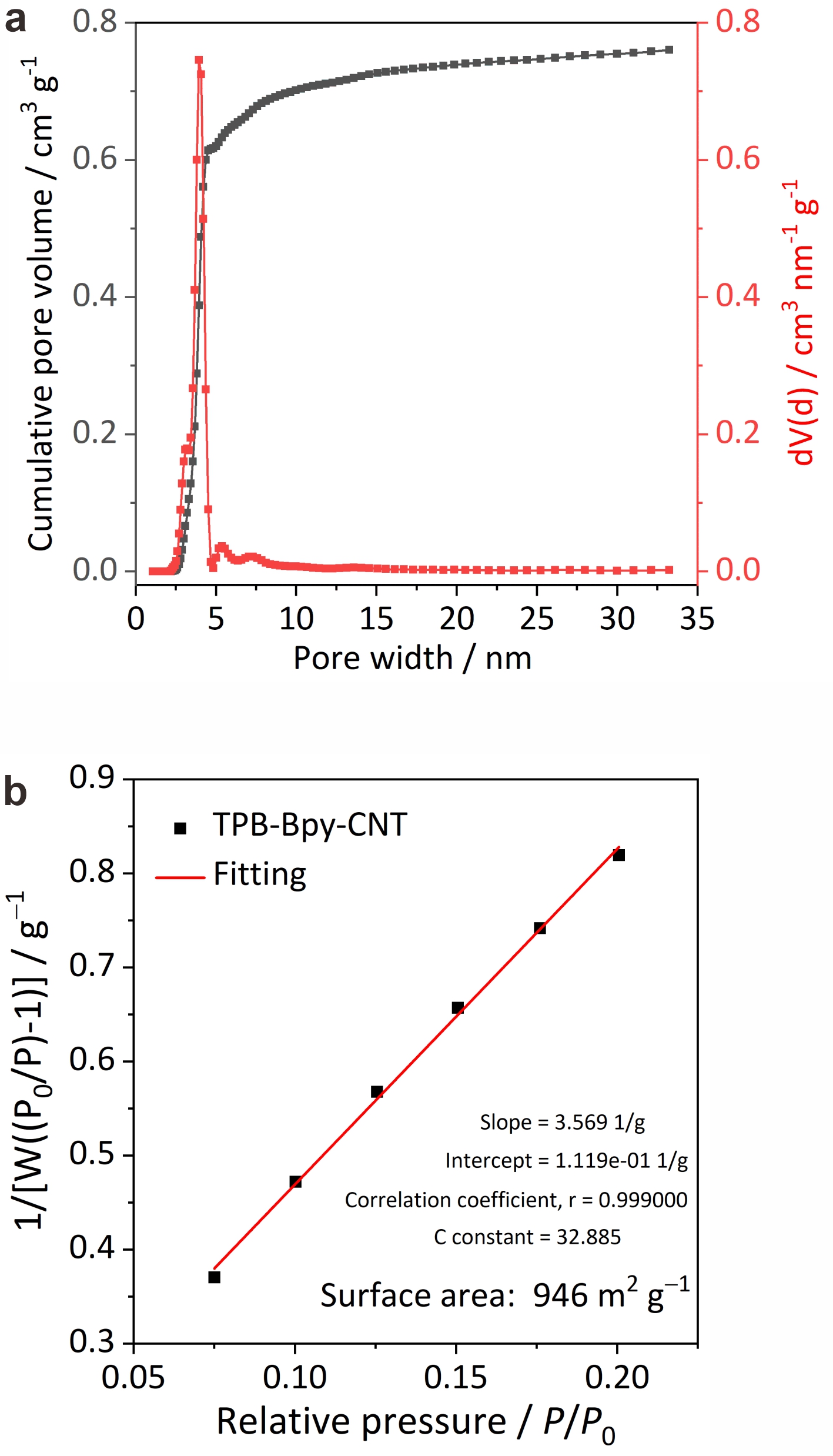


**Supplementary Figure S11.** Pore-size distribution (a) and BET plot (b) of TPB-Bpy-CNT.


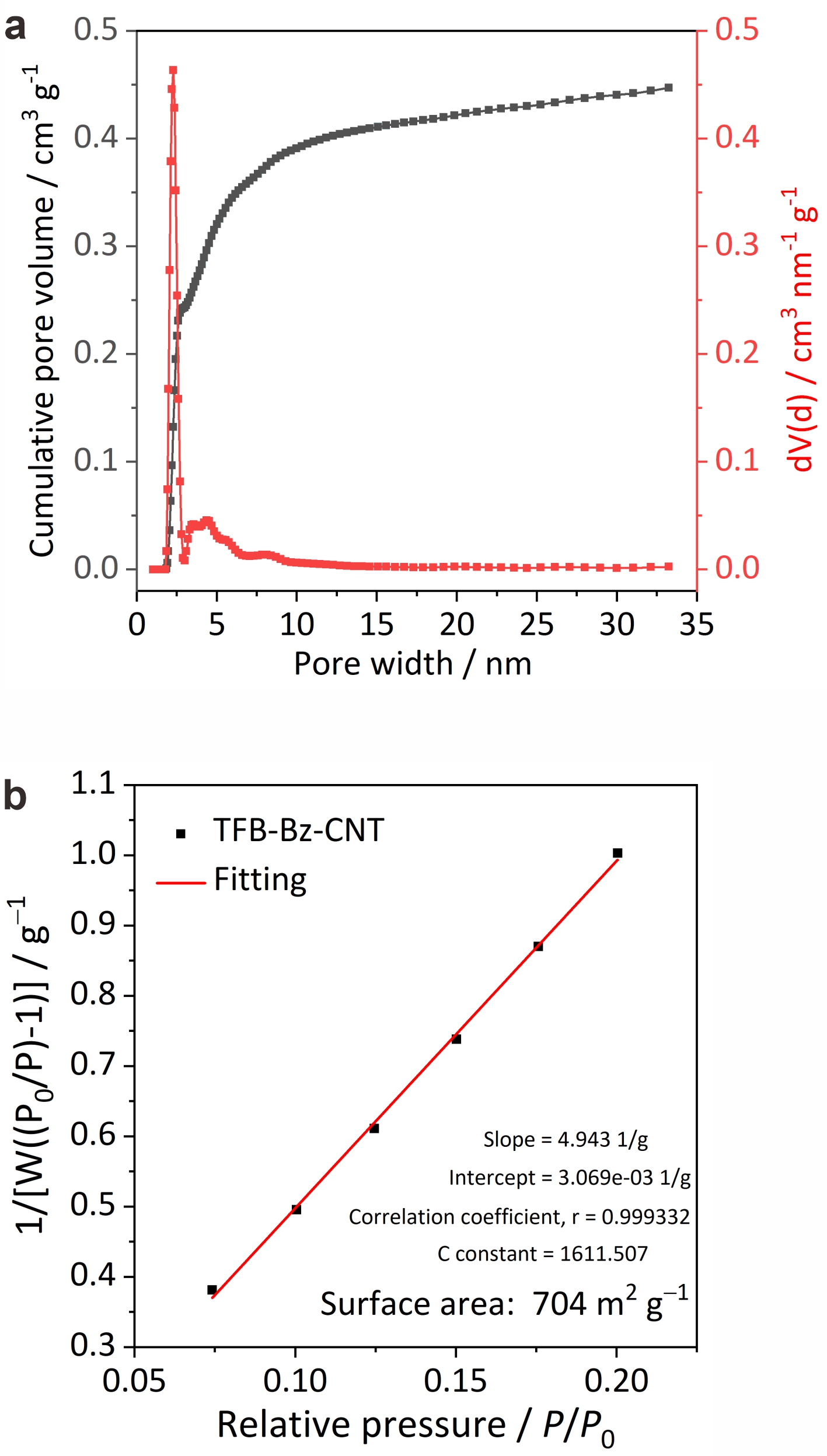


**Supplementary Figure S12.** Pore-size distribution (a) and BET plot (b) of TFB-Bz-CNT.


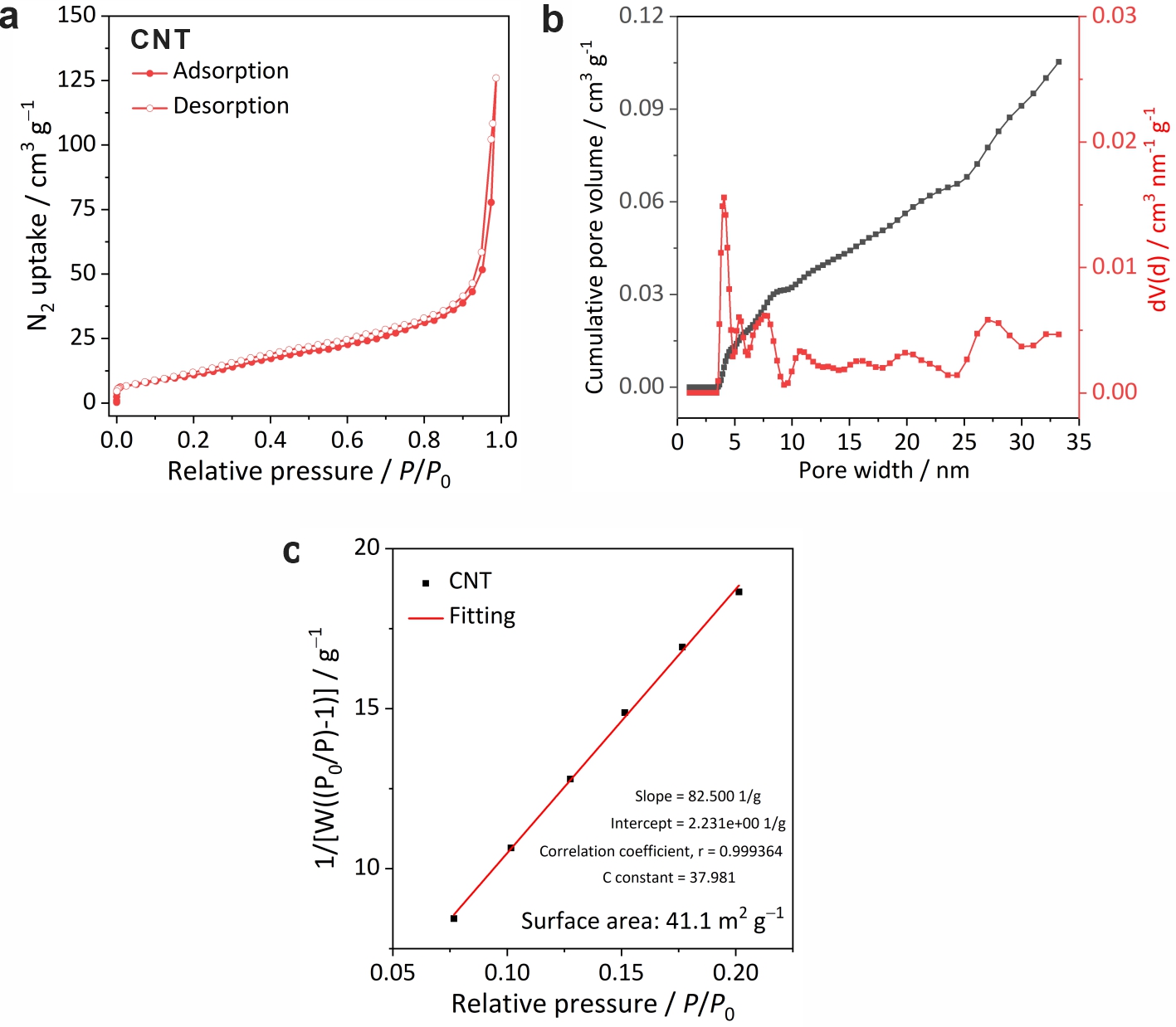


**Supplementary Figure S13.** N_2_ adsorption (filled) and desorption (empty) isotherm profiles at 77 K (a), pore-size distribution (b) and BET plot (c) of bare CNT.


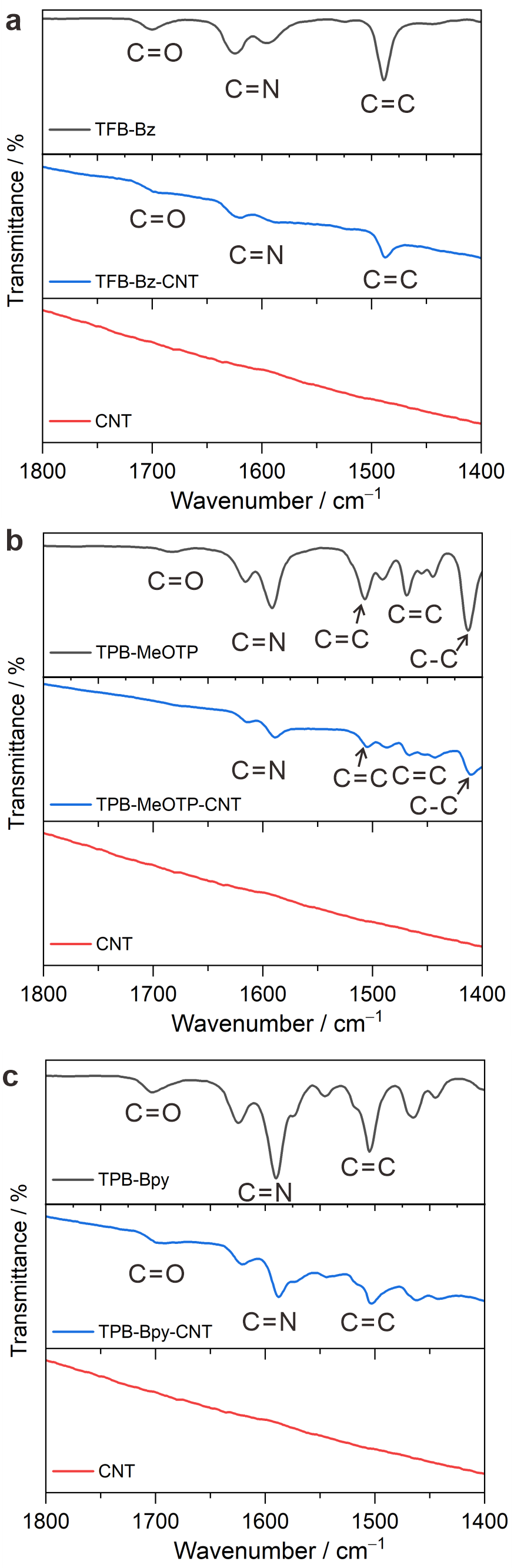


**Supplementary Figure S14.** FT-IR spectra of colloidal COFs, COF-CNT nanohybrid and CNT.


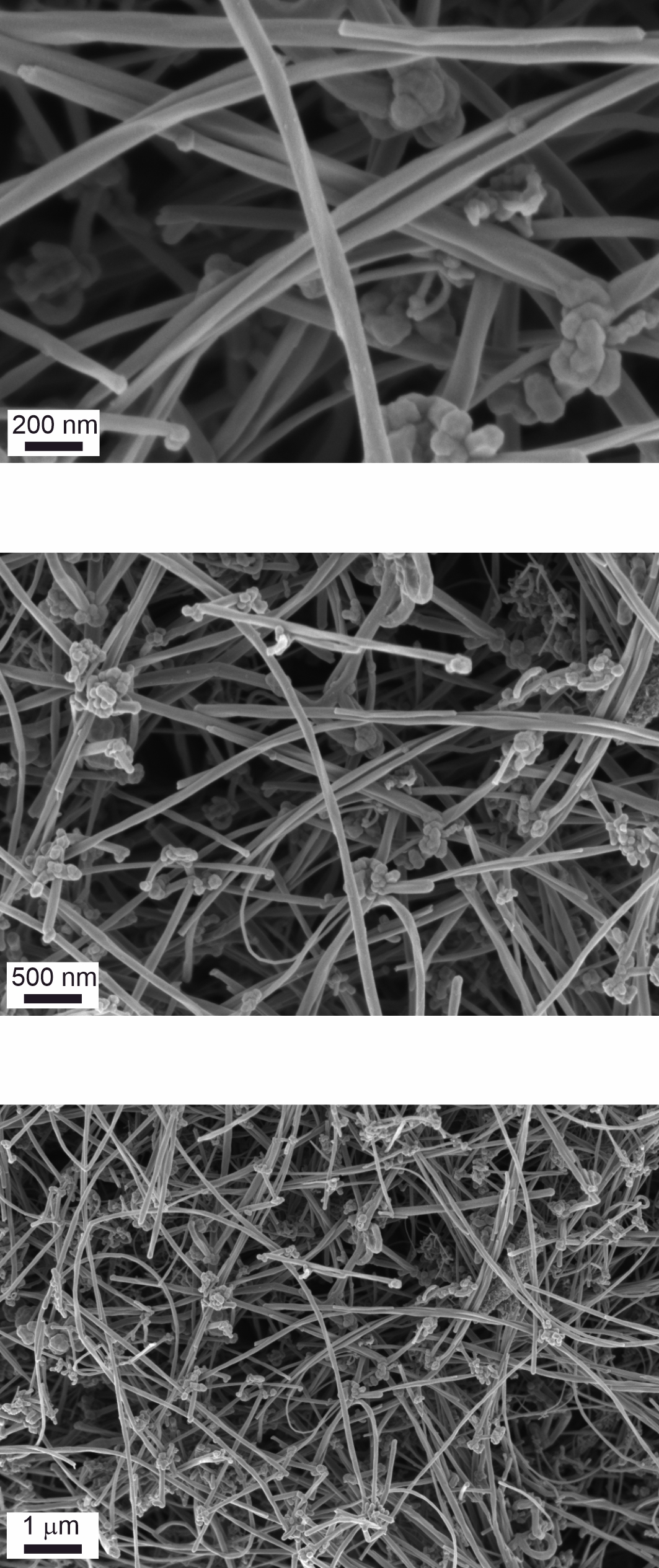


**Supplementary Figure S15.** SEM images of bare CNT.


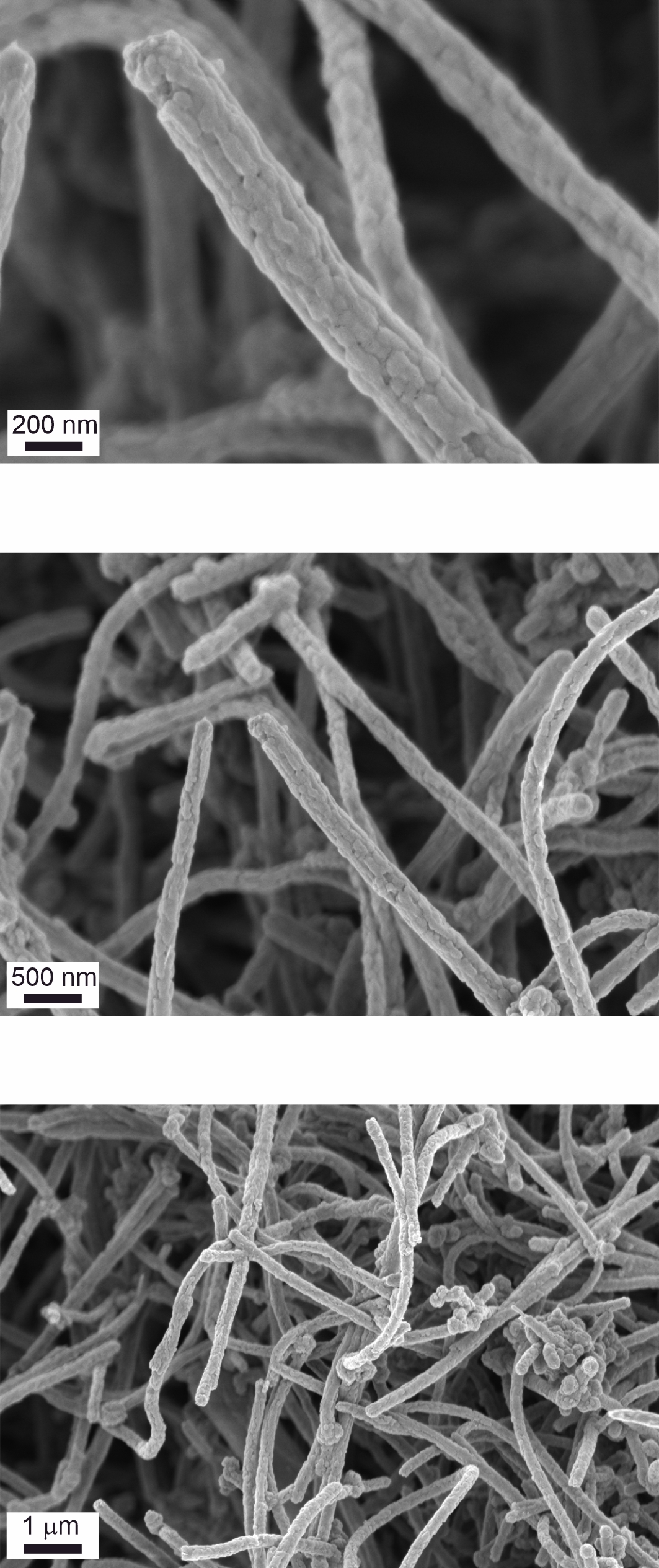


**Supplementary Figure S16.** SEM images of TPB-MeOTP-CNT.


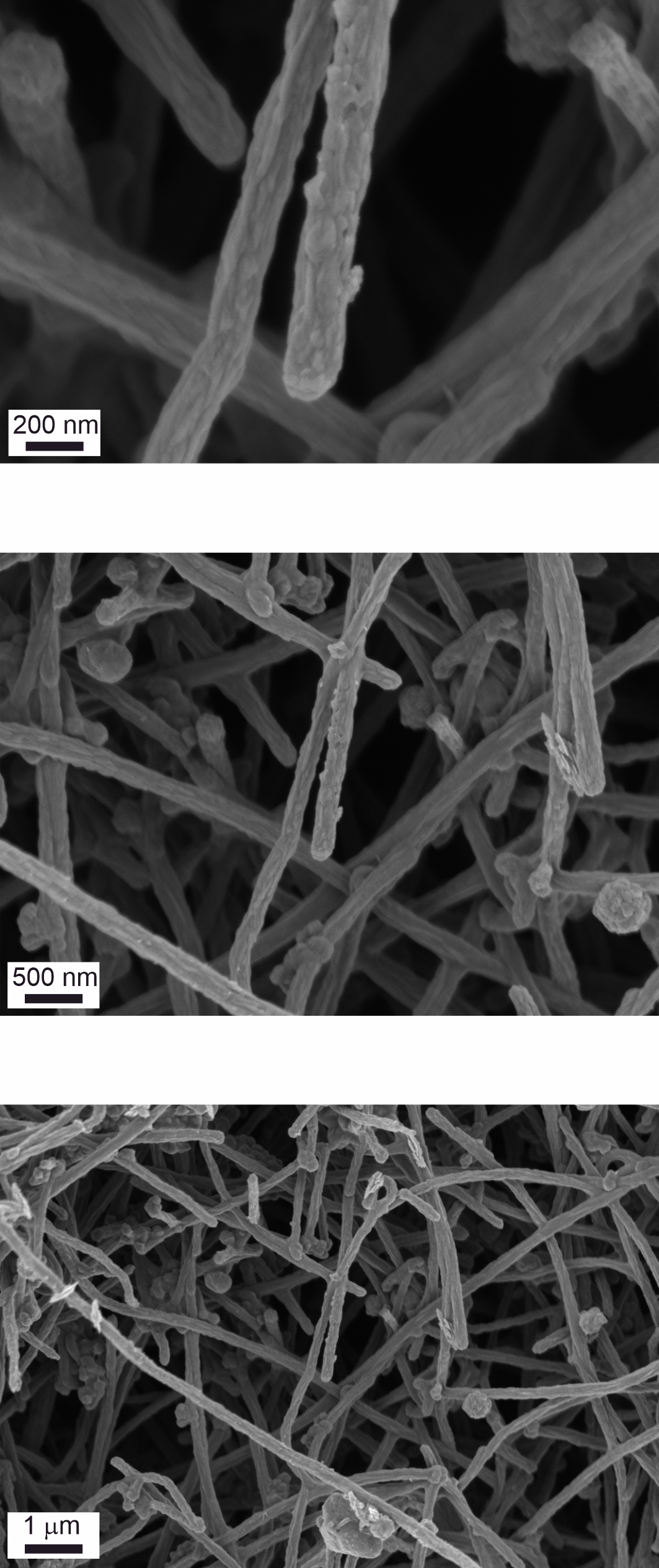


**Supplementary Figure S17.** SEM images of TPB-Bpy-CNT.


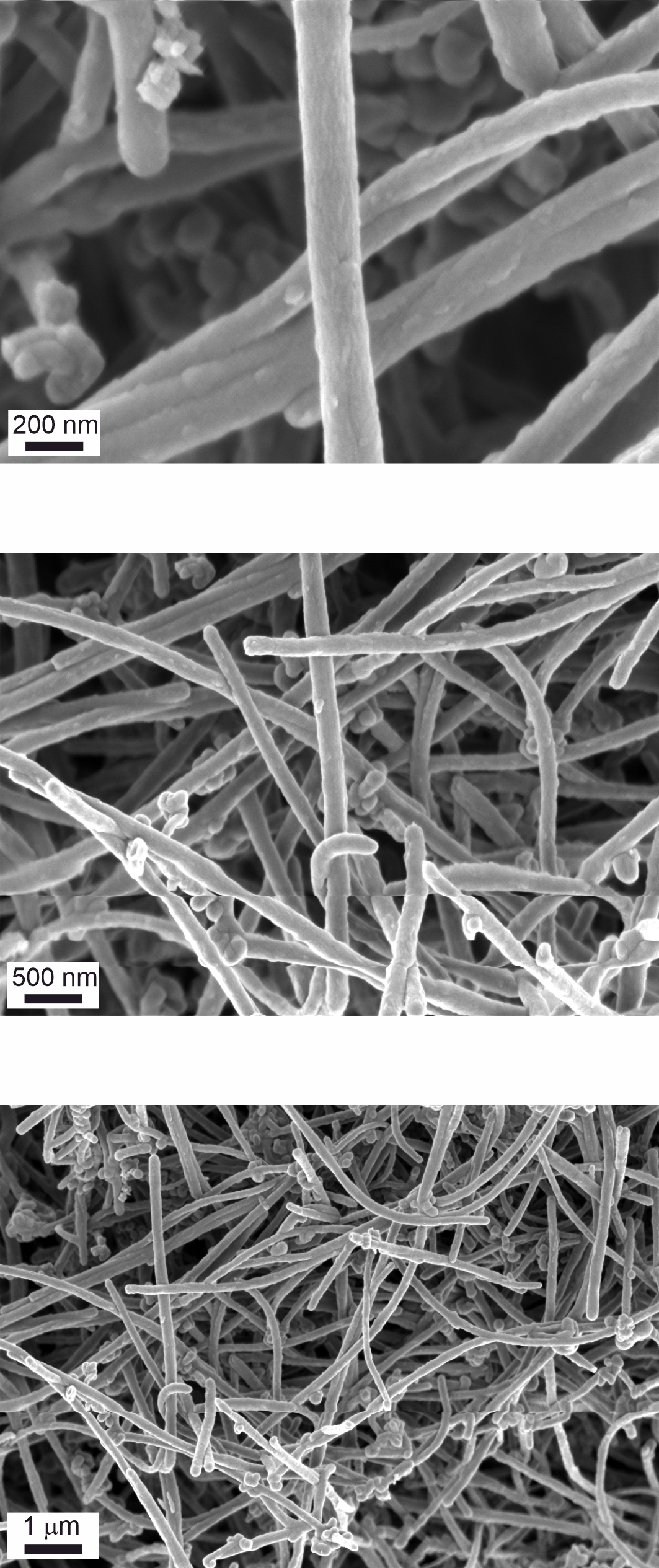


**Supplementary Figure S18.** SEM images of TFB-Bz-CNT.


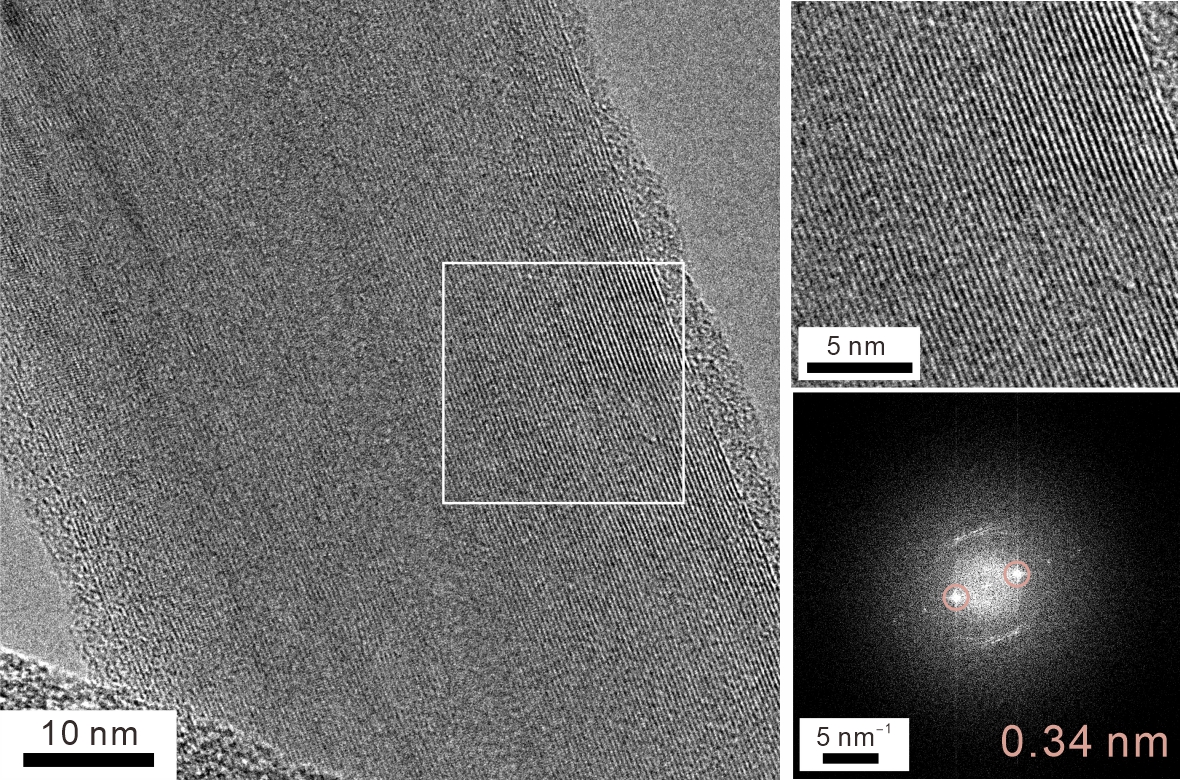


**Supplementary Figure S19.** BF-TEM image of CNT showing the 002 reflection of CNT. Zoom-in BF-TEM image and FFT pattern of the boxed region are shown on the right. Predominant fringe distance is displayed in the FFT image.

.


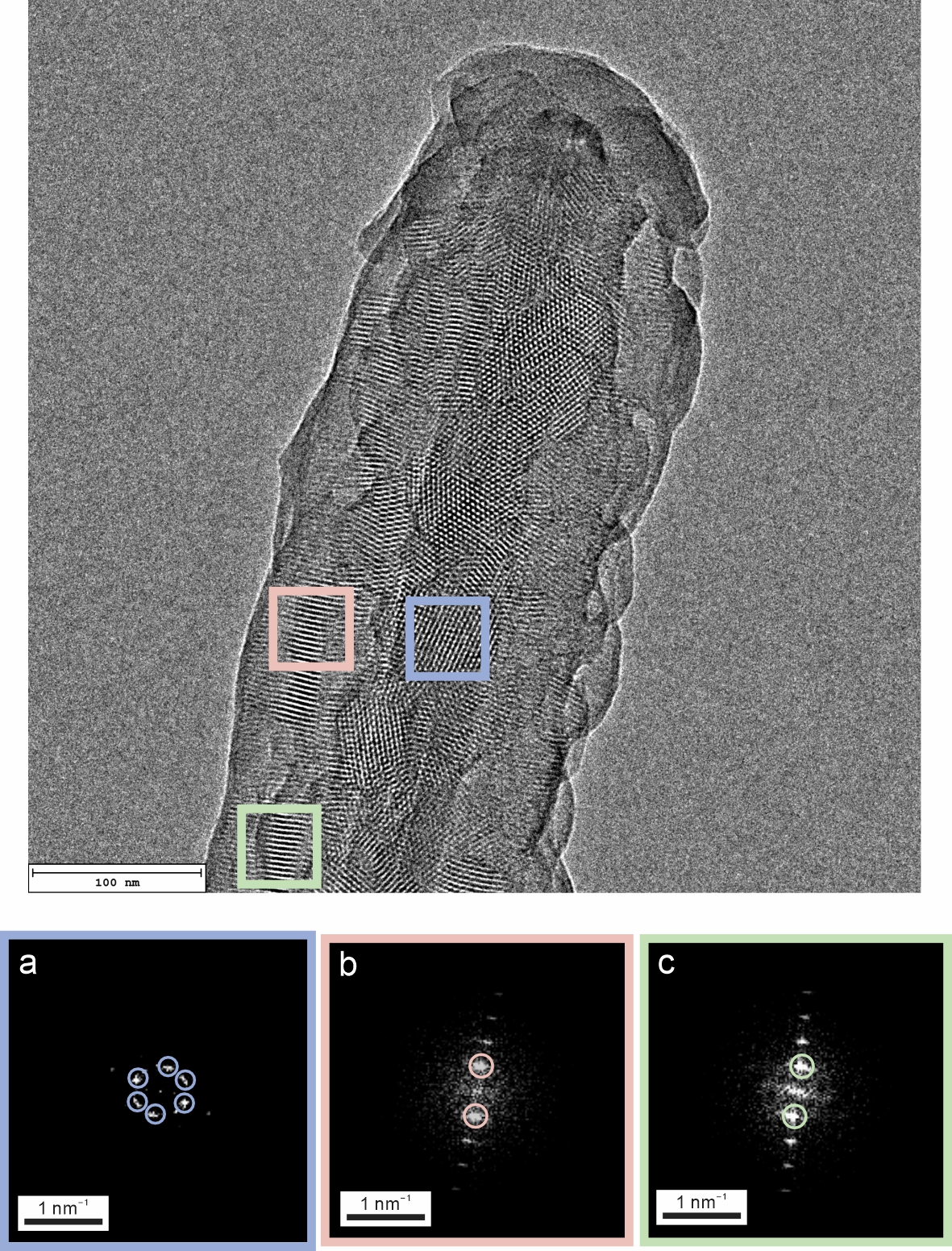


**Supplementary Figure S20.** BF-TEM image and FFT patterns of TPB-MeOTP-CNT. The BF-TEM image is identical with **Figure 3b**. The lattice fringes in the FFT patterns corresponds to the 100 reflections of TPB-MeOTP-CNT COF.


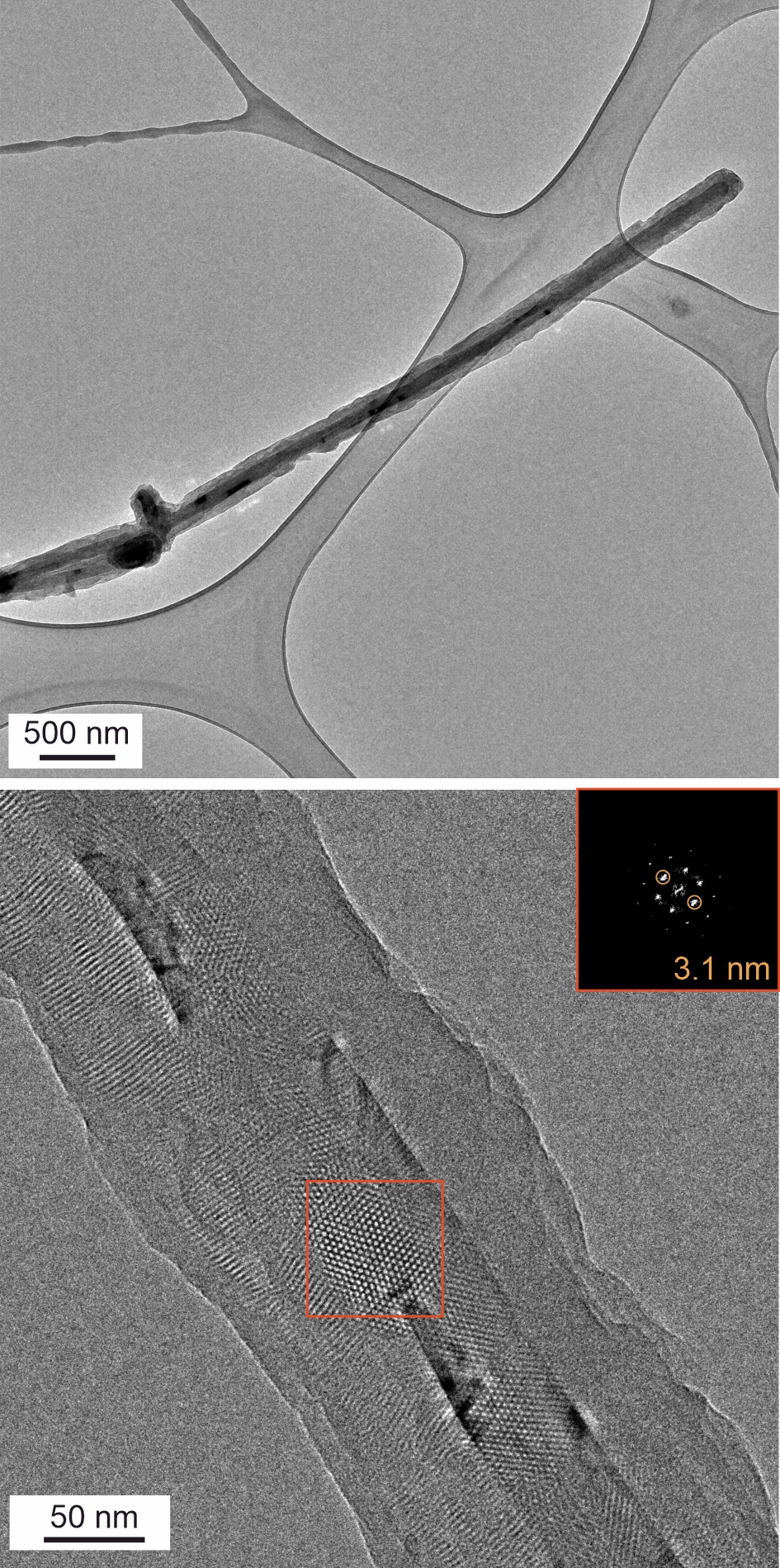


**Supplementary Figure S21.** BF-TEM image of TPB-MeOTP-CNT with lower and higher magnification. A different zoom-in region than **Figure 3b** is displayed. Inset figure shows the FFT pattern and the calculated lattice fringe corresponding to 100 reflection.


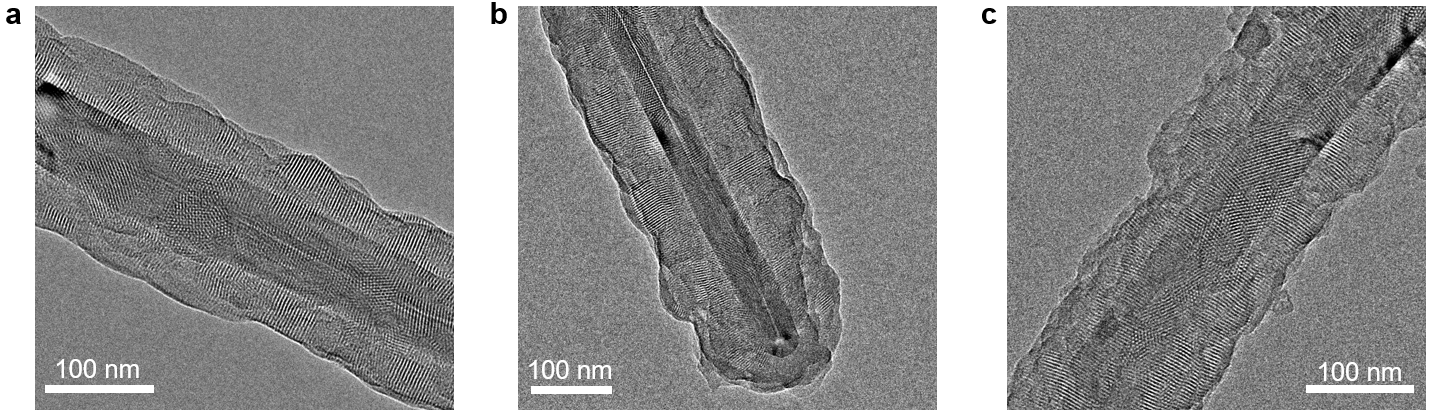


**Supplementary Figure S22.** TEM images of TPB-MeOTP-CNT prepared from three independent syntheses, demonstrating good reproducibility.


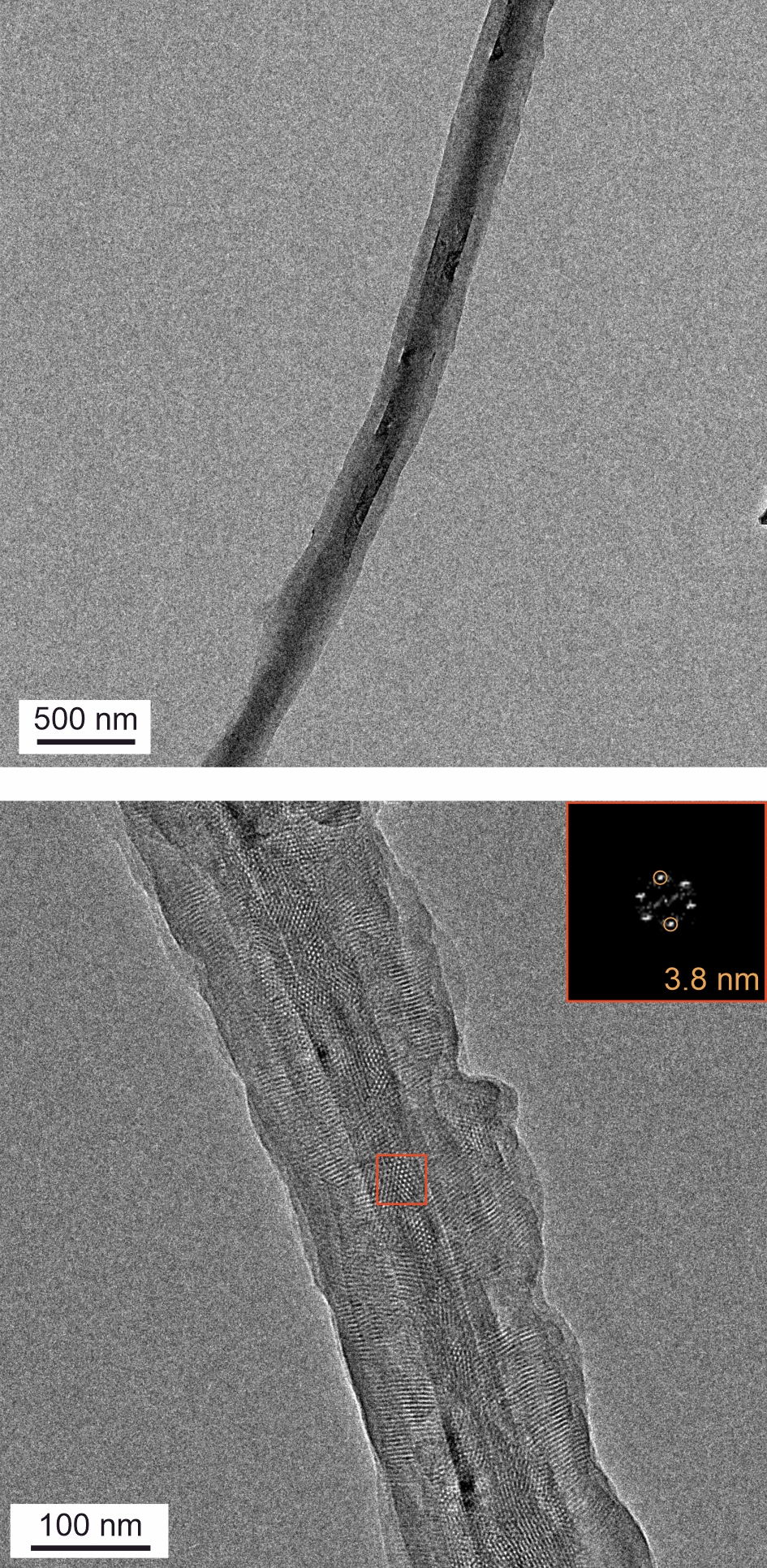


**Supplementary Figure S23.** BF-TEM images of TPB-Bpy-CNT with lower and higher magnification. Inset figure shows the FFT pattern and the calculated lattice fringe corresponding to 100 reflection.


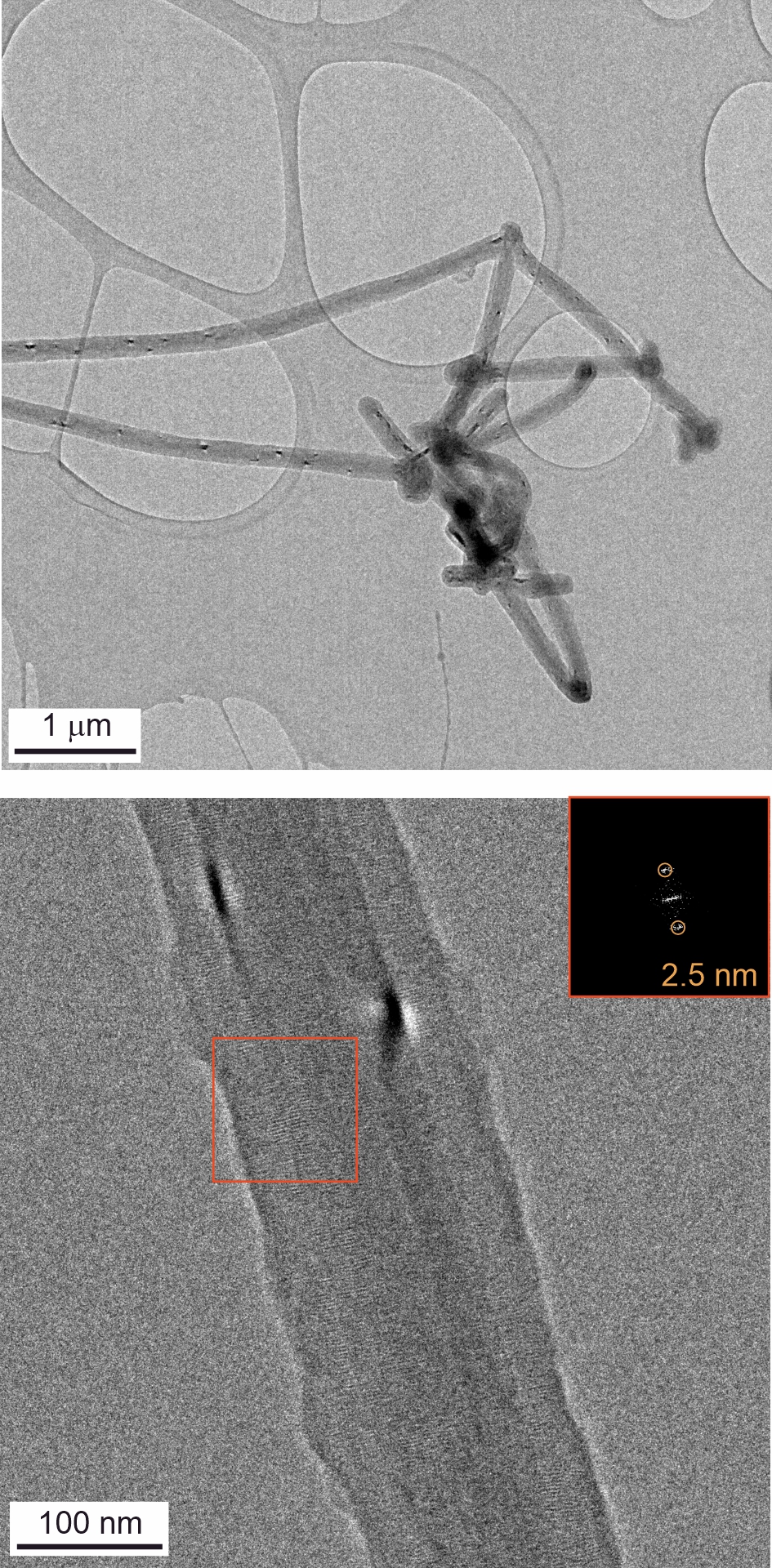


**Supplementary Figure S24.** BF-TEM images of TFB-Bz-CNT with lower and higher magnification. Inset figure shows the FFT pattern and the calculated lattice fringe.


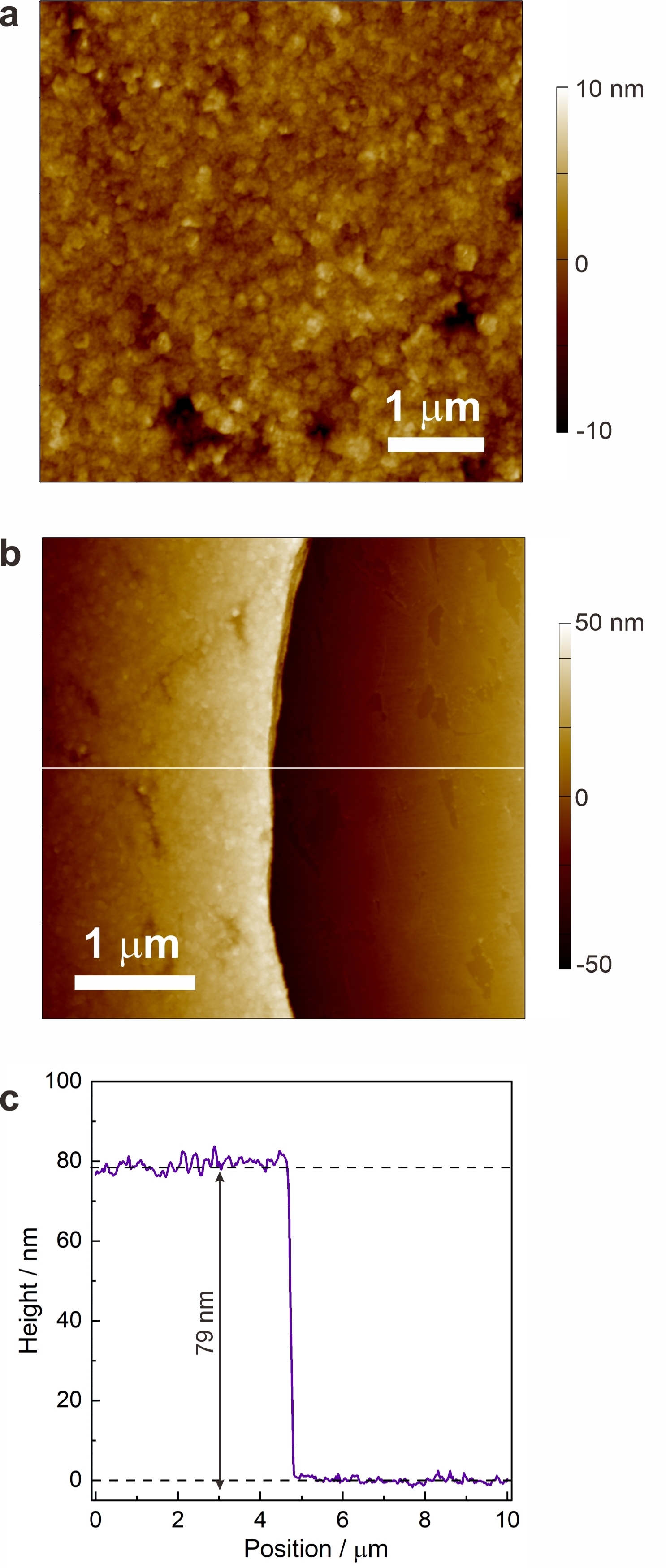


**Supplementary Figure S25.** AFM measurements for the TPB-Bpy COF film grown on MLG SiO_2_/Si wafer; (a) shows the AFM height image of the film. The film roughness is determined to be 1.8 nm. (b, c) show the AFM height image and the height plot along the white line in the height image, respectively. To measure the film thickness, part of the TPB-Bpy COF film was removed as shown at the right part of Figure S24b.


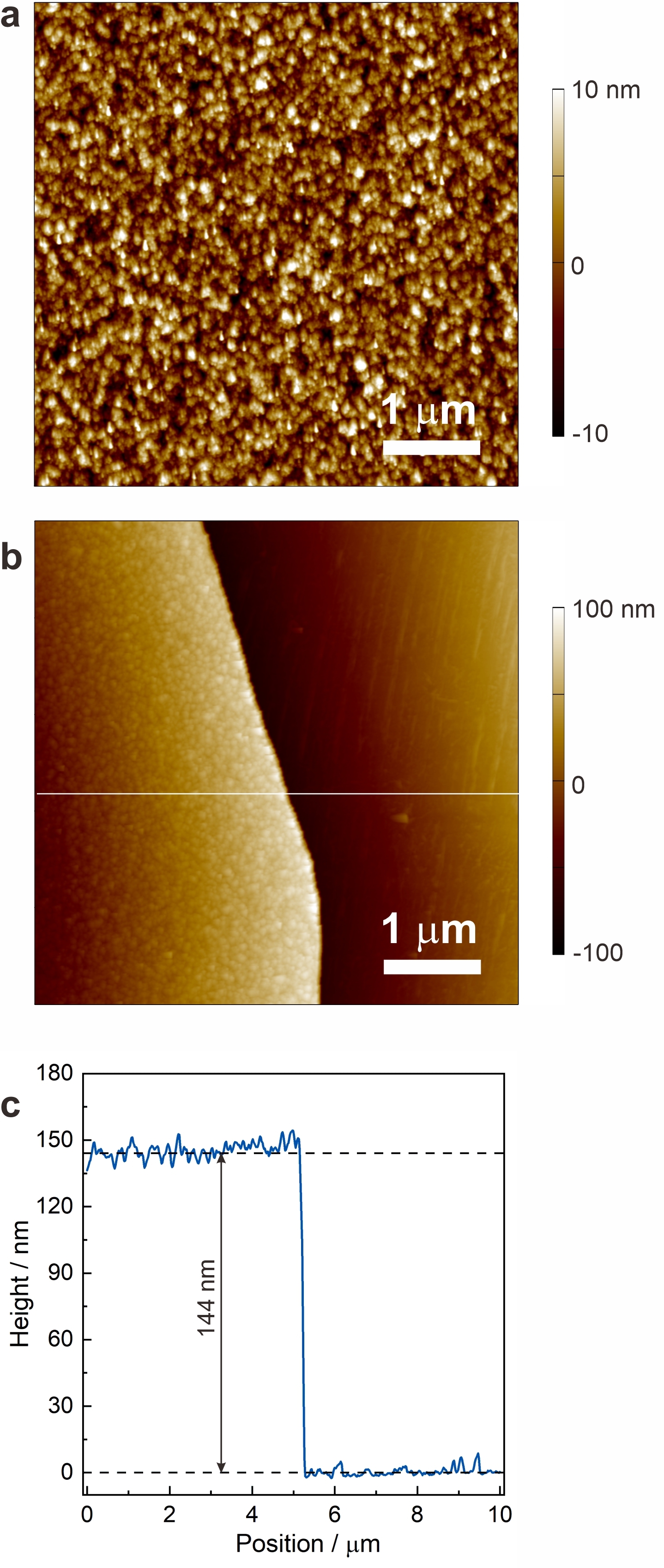


**Supplementary Figure S26.** AFM measurements for the TFB-Bz COF film grown on MLG SiO_2_/Si wafer; (a) shows the AFM height image of the film. The film roughness is determined to be 3.8 nm. (b, c) show the AFM height image and the height plot along the white line in the height image, respectively. To measure the film thickness, part of the TFB-Bz COF film was removed as shown at the right part of Figure S24b.


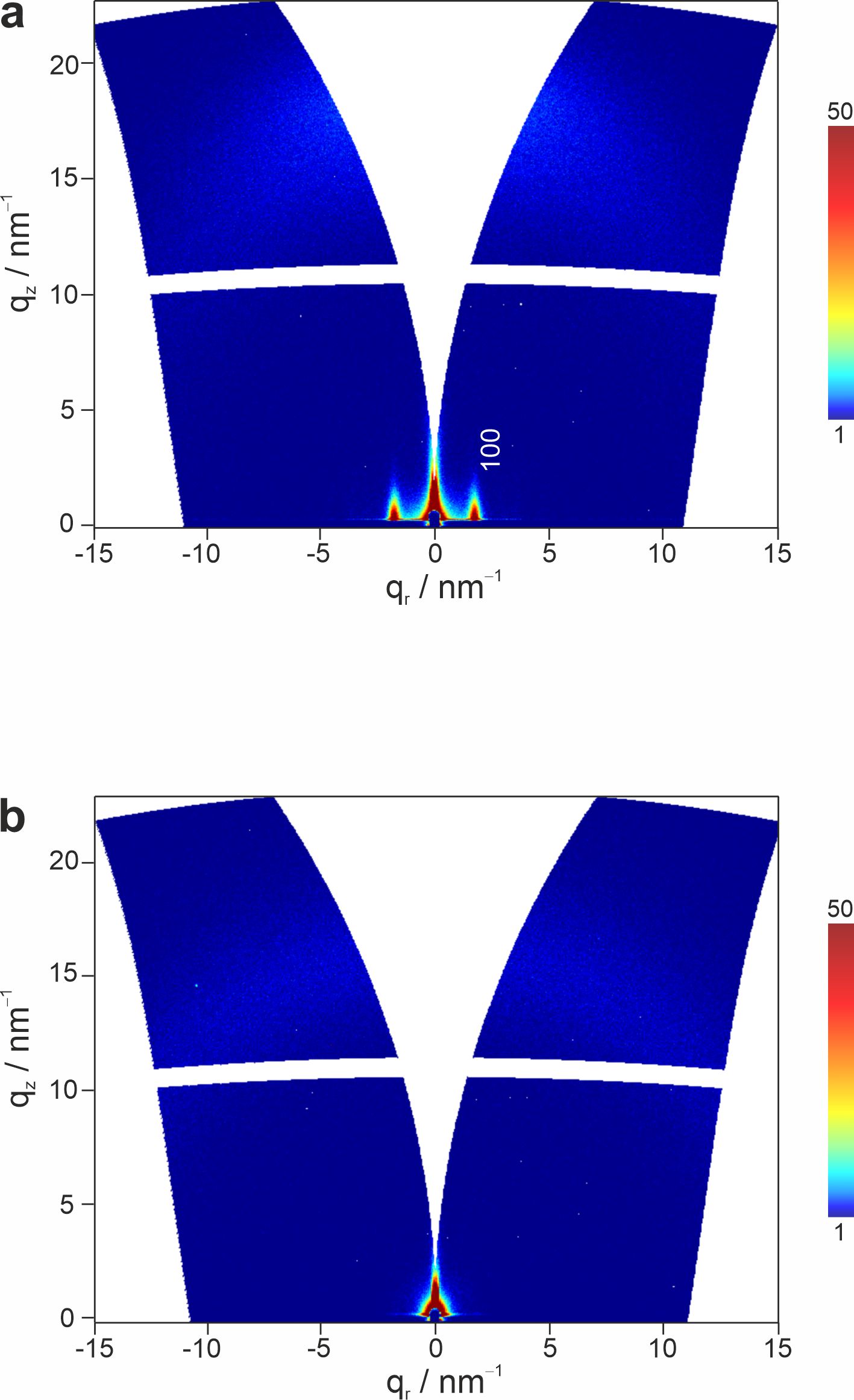


**Supplementary Figure S27.** GIWAXS 2D patterns of TPB-Bpy films grown on MLG SiO_2_/Si wafer (a) and bare SiO_2_/Si wafer (b). The film on MLG SiO_2_/Si wafer show a low arching in-plane reflection at 1.8 nm ^‒1^.


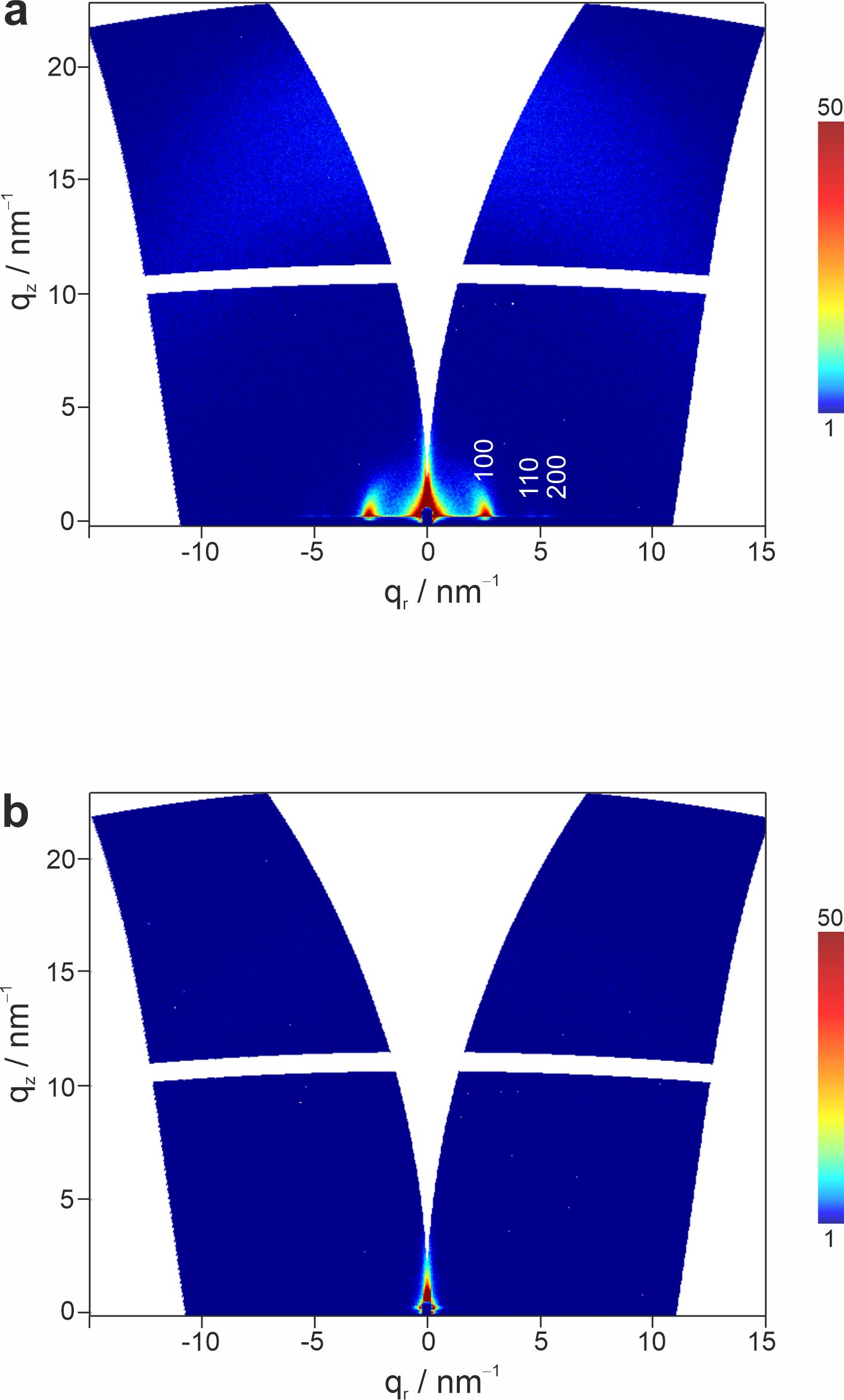


**Supplementary Figure S28.** GIWAXS 2D patterns of TFB-Bz films grown on MLG SiO_2_/Si wafer (a) and bare SiO_2_/Si wafer (b). The film on MLG SiO_2_/Si wafer show low arching in-plane reflections at 2.6, 4.6 and 5.3 nm ^‒1^.


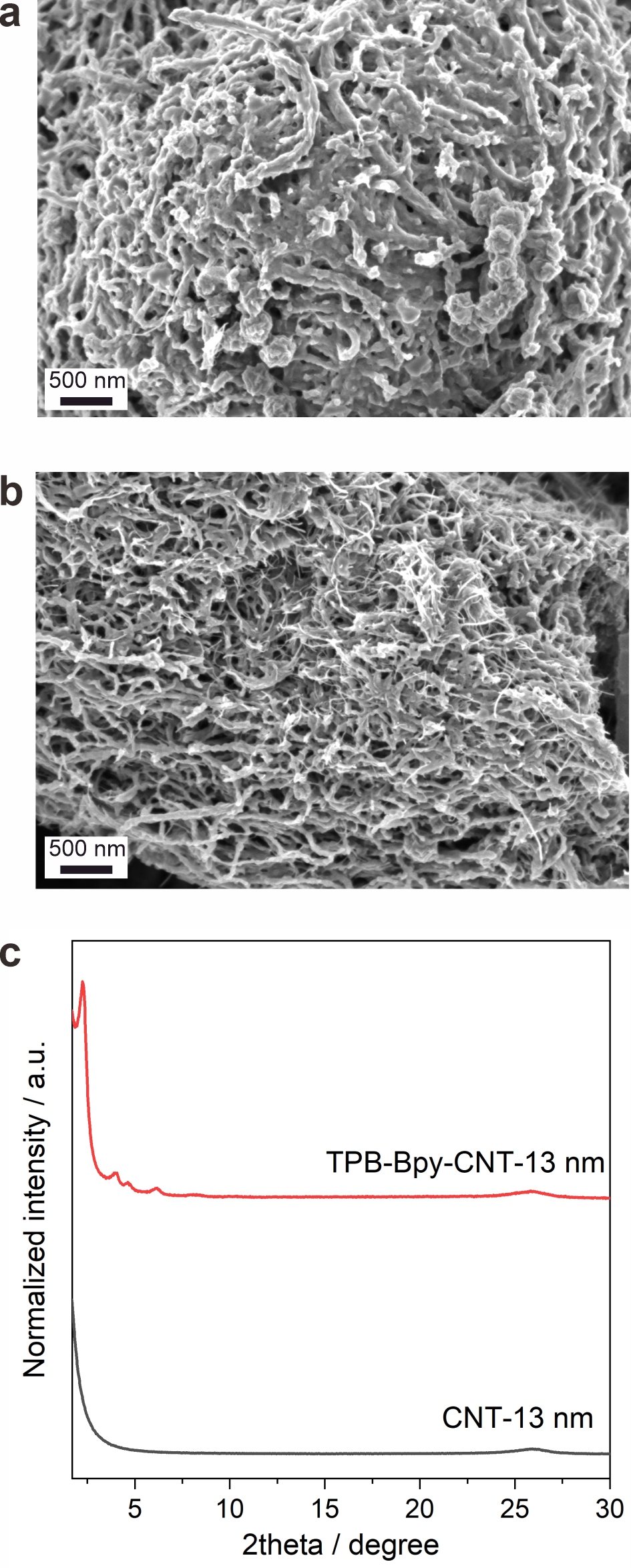


**Supplementary Figure S29.** SEM images (a, b) and PXRD patterns (Cu-K*_α_*_1_) (c) of TPB-Bpy-CNT-13 nm. SEM images (a) and (b) were acquired from the sample but different regions. The SEM images indicate that TPB-Bpy COF tends to grow across CNT-13 surface, instead of forming nanohybrid core-shell structures. PXRD pattern of TPB-Bpy-CNT-13 nm show a strong 100 reflection of TPB-Bpy COF at 2θ = 2.25°, and the presence of other higher order reflections, indicating the high crystallinity of the TPB-Bpy-CNT-13 nm product.


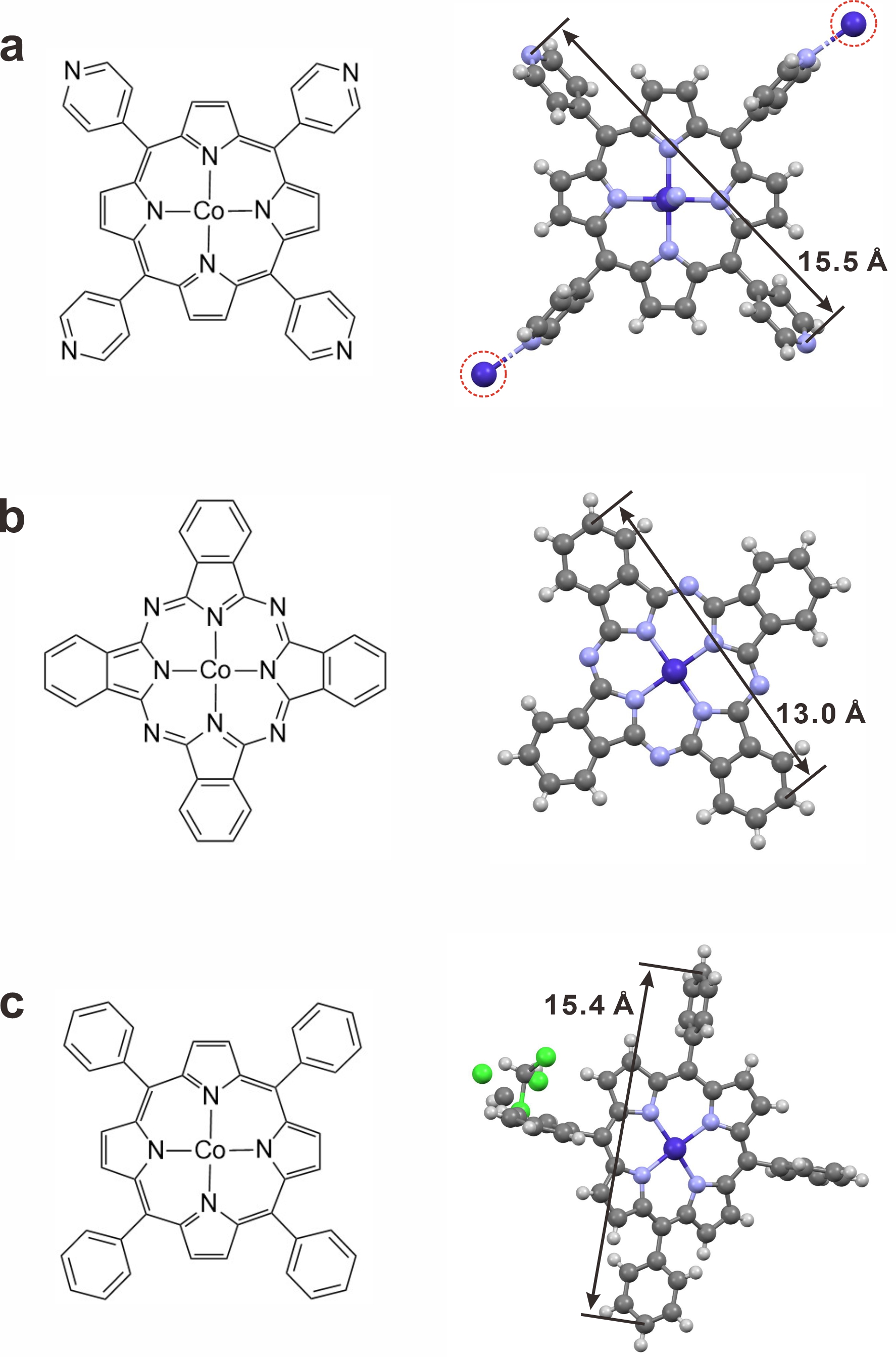


**Supplementary Figure S30.** Molecular size of CoTPyP (a), CoPc (b), and CoTPP (c) extracted from reported crystal structures deposited in the Cambridge Crystallographic Data Center (CCDC database).^3-5^ The red circled atom in the crystal structure of CoTPyP is an additional cobalt atom facilitating the coordination framework formation. The CoTPP crystal (Figure S30c) is obtained as a dichloromethane solvate, and therefore contains chlorine atoms (green color) within the structure.


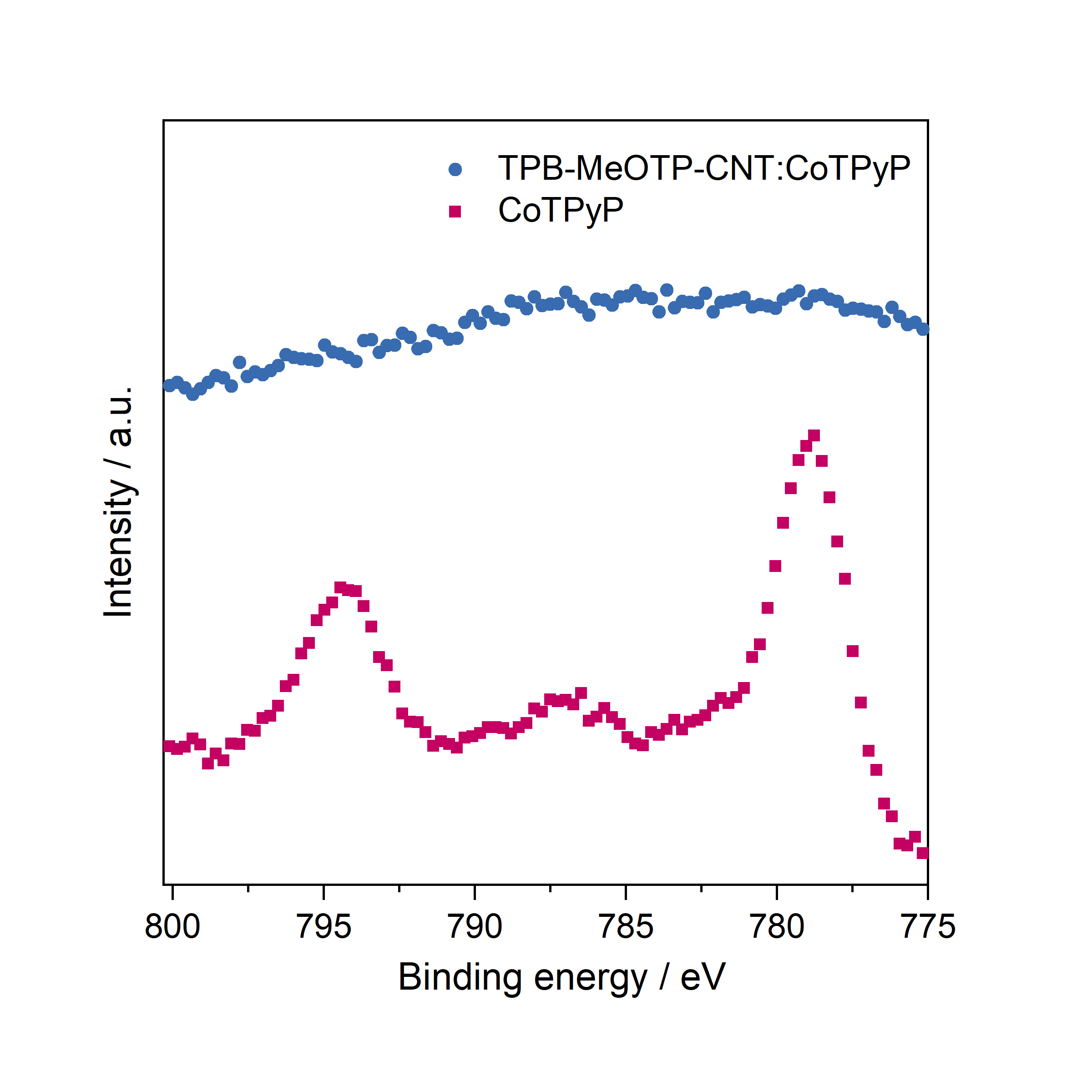


**Supplementary Figure S31.** X-ray photoelectron spectra of Co 2p of TPB-MeOTP-CNT:CoTPyP and CoTPyP. It can be seen that CoTPyP clearly shows two main peaks at 778.8 eV and 794.2 eV, corresponding to the 2*p*_3/2_ and 2*p*_1/2_ core levels of Co(II) ions, respectively. However, the characteristic Co(II) signals are not present in TPB-MeOTP-CNT:CoTPyP. This is because the low loading amount of Co(II) in TPB-MeOTP-CNT falls below the detection limits of XPS measurements.


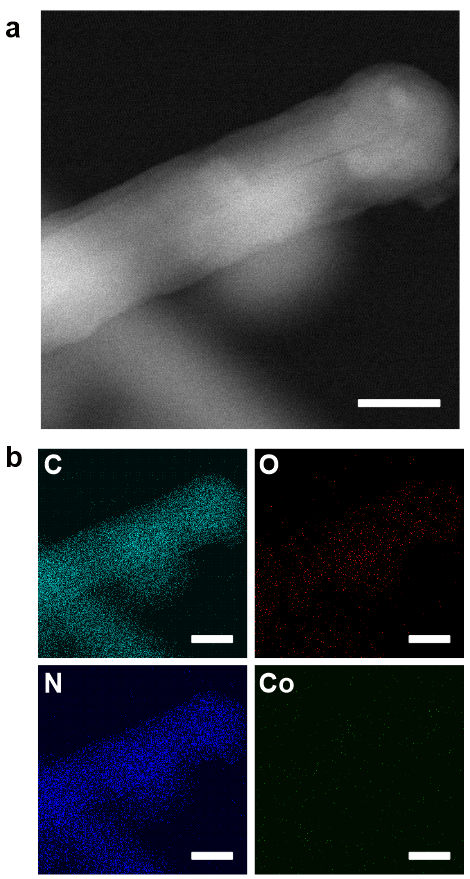


**Supplementary Figure S32.** High angle annular dark field (HAADF)-STEM images of TPB-MeOTP-CNT:CoTPyP and corresponding EDX maps of the chemical composition and distribution, including carbon, oxygen, nitrogen and cobalt (scale bar: 500 nm). The result shows that no significant CoTPyP aggregation is observed for TPB-MeOTP-CNT:CoTPyP. Further identification of the location of Co(II) is not feasible due to the low cobalt loading amount in TPB-MeOTP-CNT:CoTPyP.


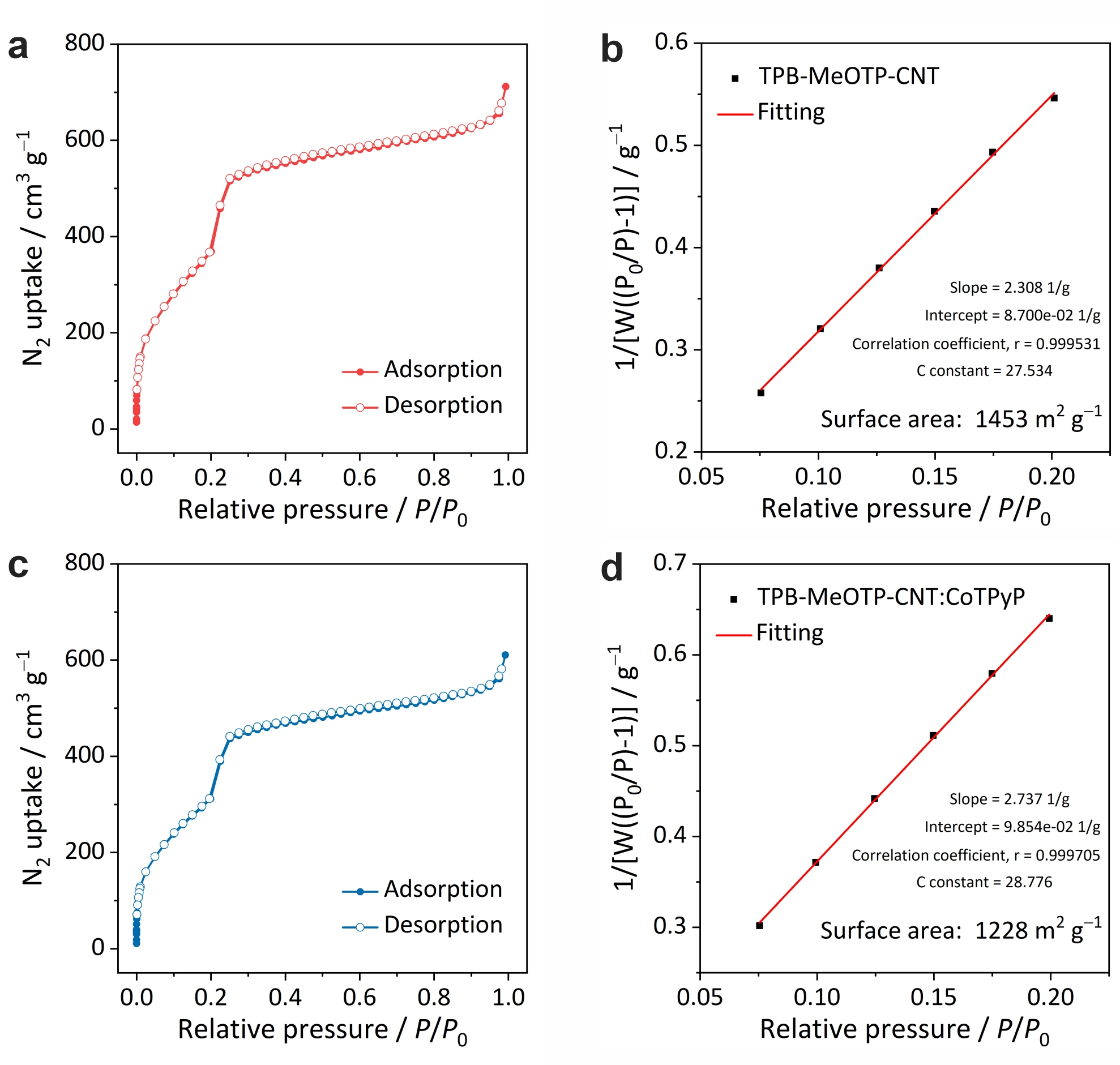


**Supplementary Figure S33.** N_2_ adsorption (filled) and desorption (empty) isotherm profiles at 77 K of TPB-MeOTP-CNT (a) and TPB-MeOTP-CNT:CoTPyP (c). BET plot of TPB-MeOTP-CNT (b) and TPB-MeOTP-CNT:CoTPyP (d). The resulting S_BET_ of TPB-MeOTP-CNT (b) and TPB-MeOTP-CNT:CoTPyP is 1453 m^2^ g^−1^ and 1228 m^2^ g^−1^, respectively, implying that the S_BET_ decrease is insignificant but uniform, i.e. there is no evidence for pore blocking.


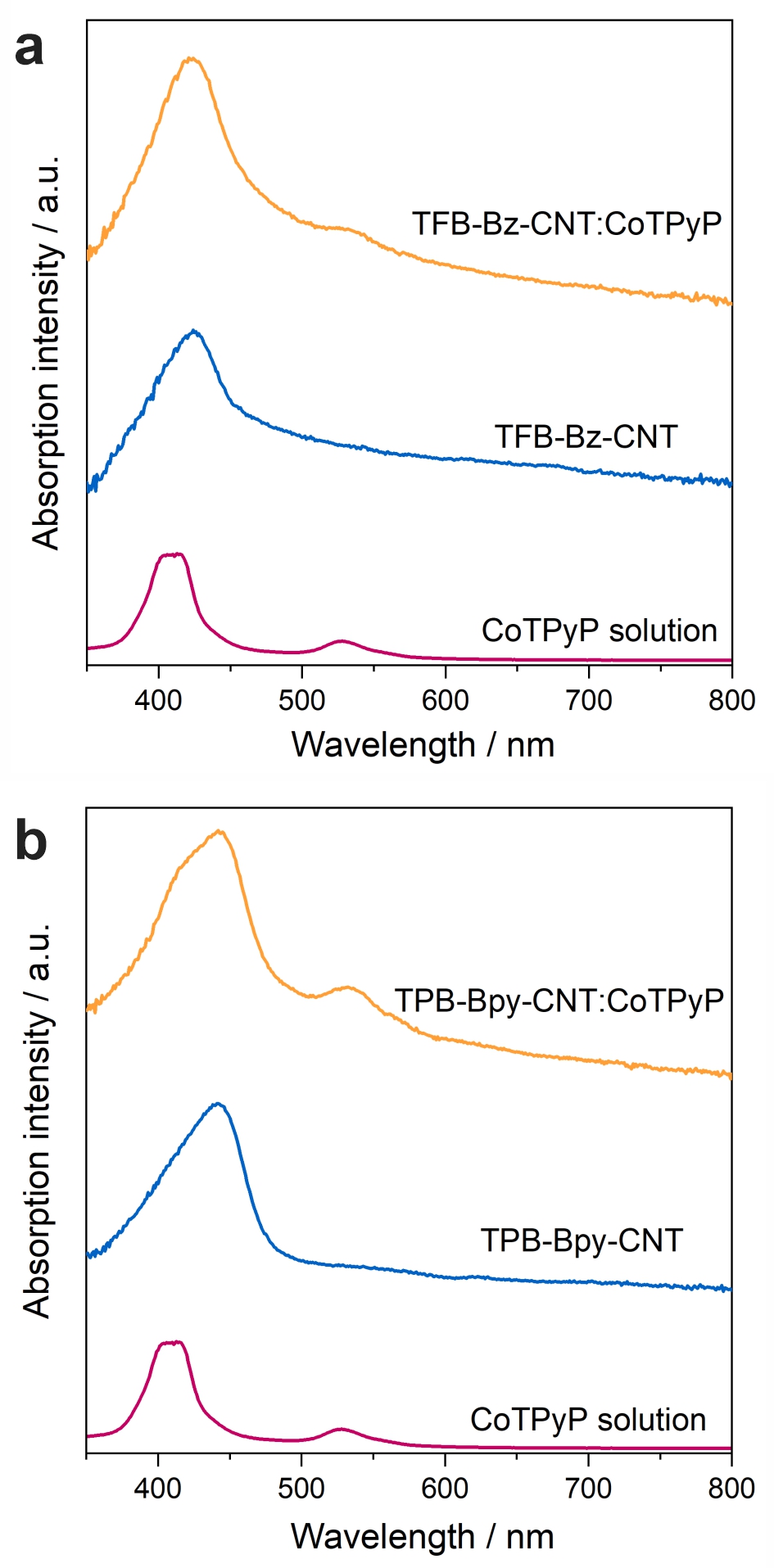


**Supplementary Figure S34.** Comparison of UV-vis spectra. (a) TFB-Bz-CNT:CoTPyP and TFB-Bz-CNT suspended in ethanol, and CoTPyP in chloroform solution. (b) TPB-Bpy-CNT:CoTPyP and TPB-Bpy-CNT suspended in ethanol, and CoTPyP in chloroform solution.


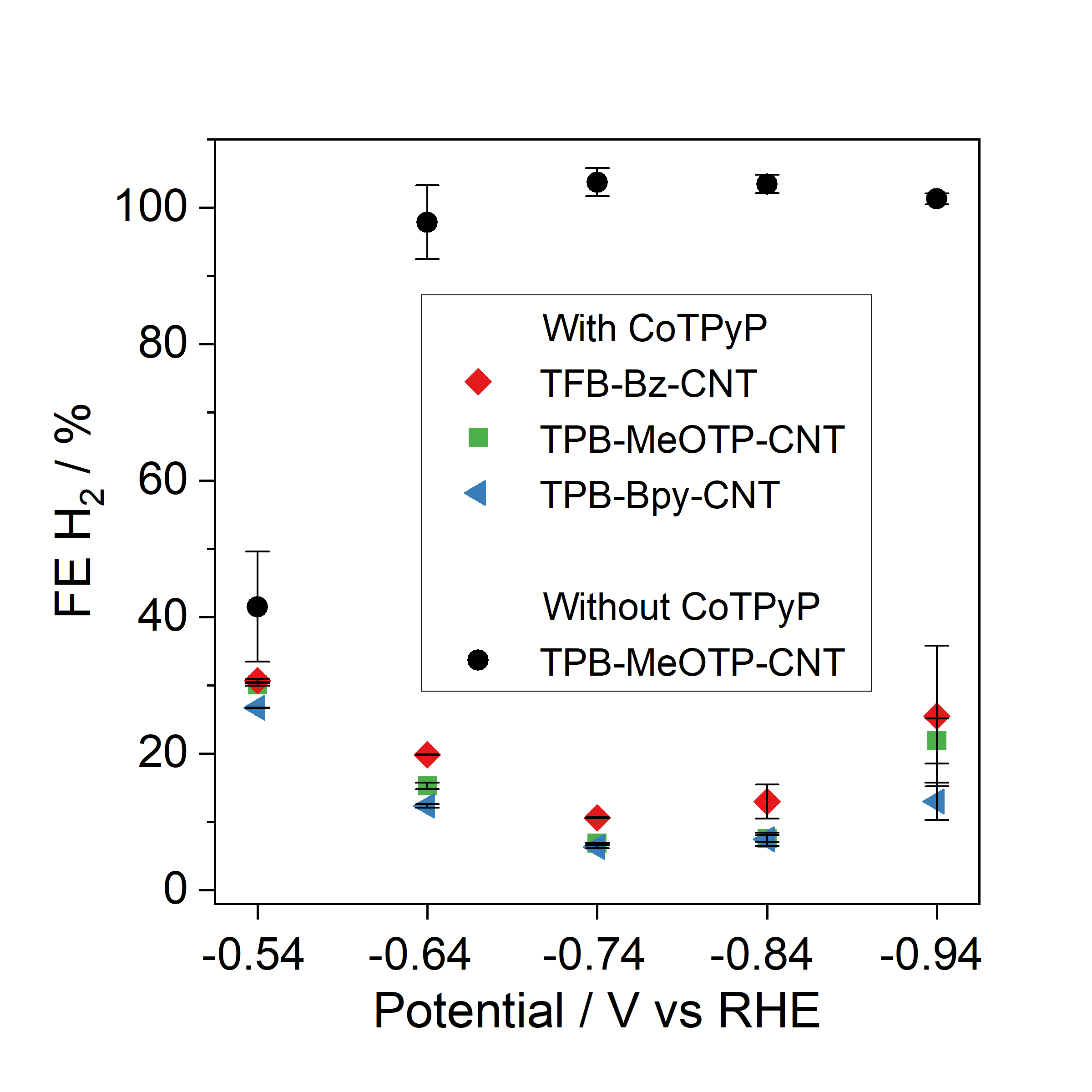


**Supplementary Figure S35.** FE(H_2_) of COF-CNTs: CoTPyP, and TPB-MeOTP-CNT without incorporating CoTPyP.


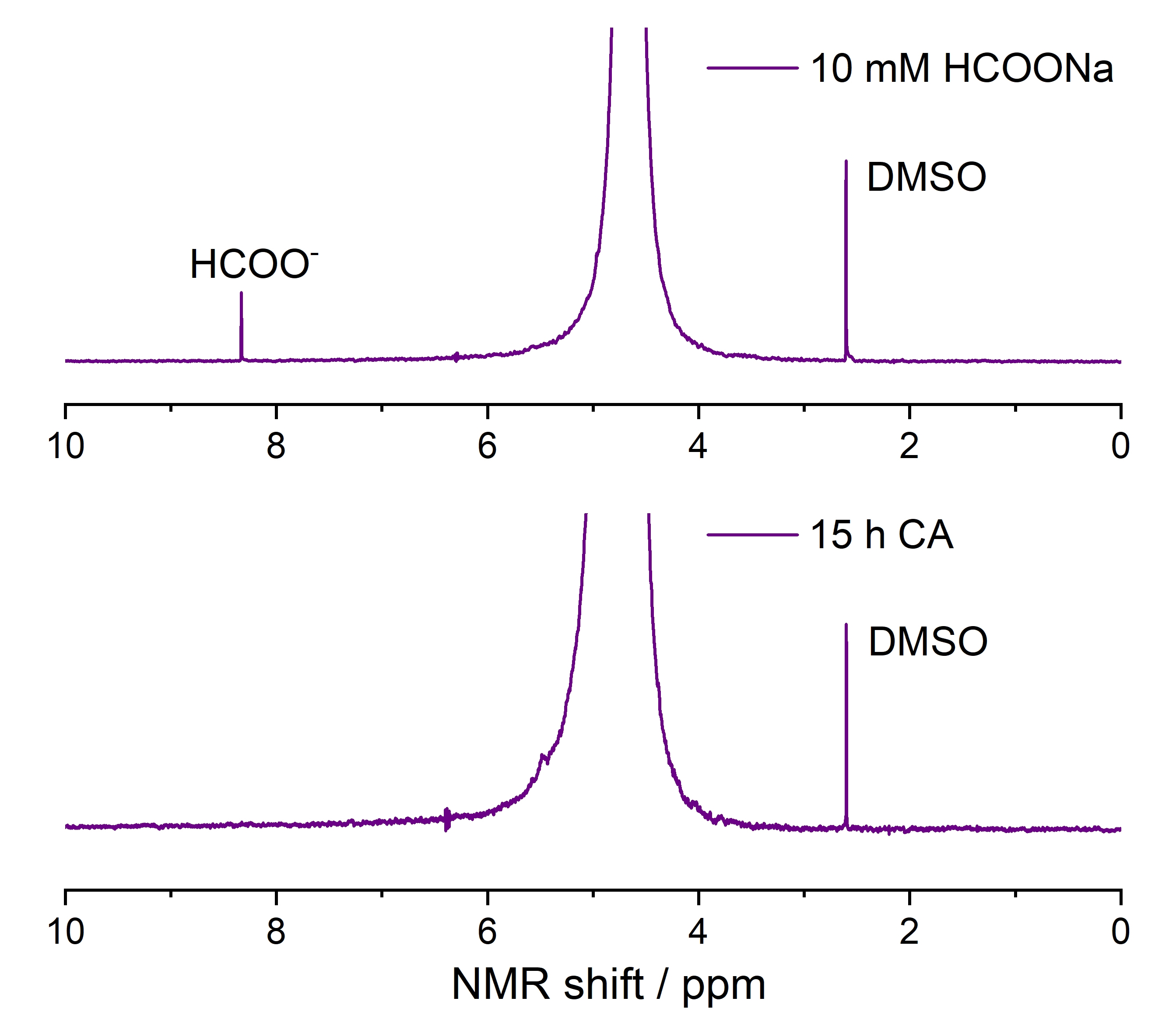


**Supplementary Figure S36.** ^1^H NMR spectrum (D_2_O, 400 MHz) of the electrolyte (bottom figure) after a chronoamperometry (CA) at −0.73 V vs RHE for 15 hours, and 10 mM HCOONa (up figure) as a reference. The result indicates that no liquid product can be found in the electrolyte, even after long-term electrolysis.


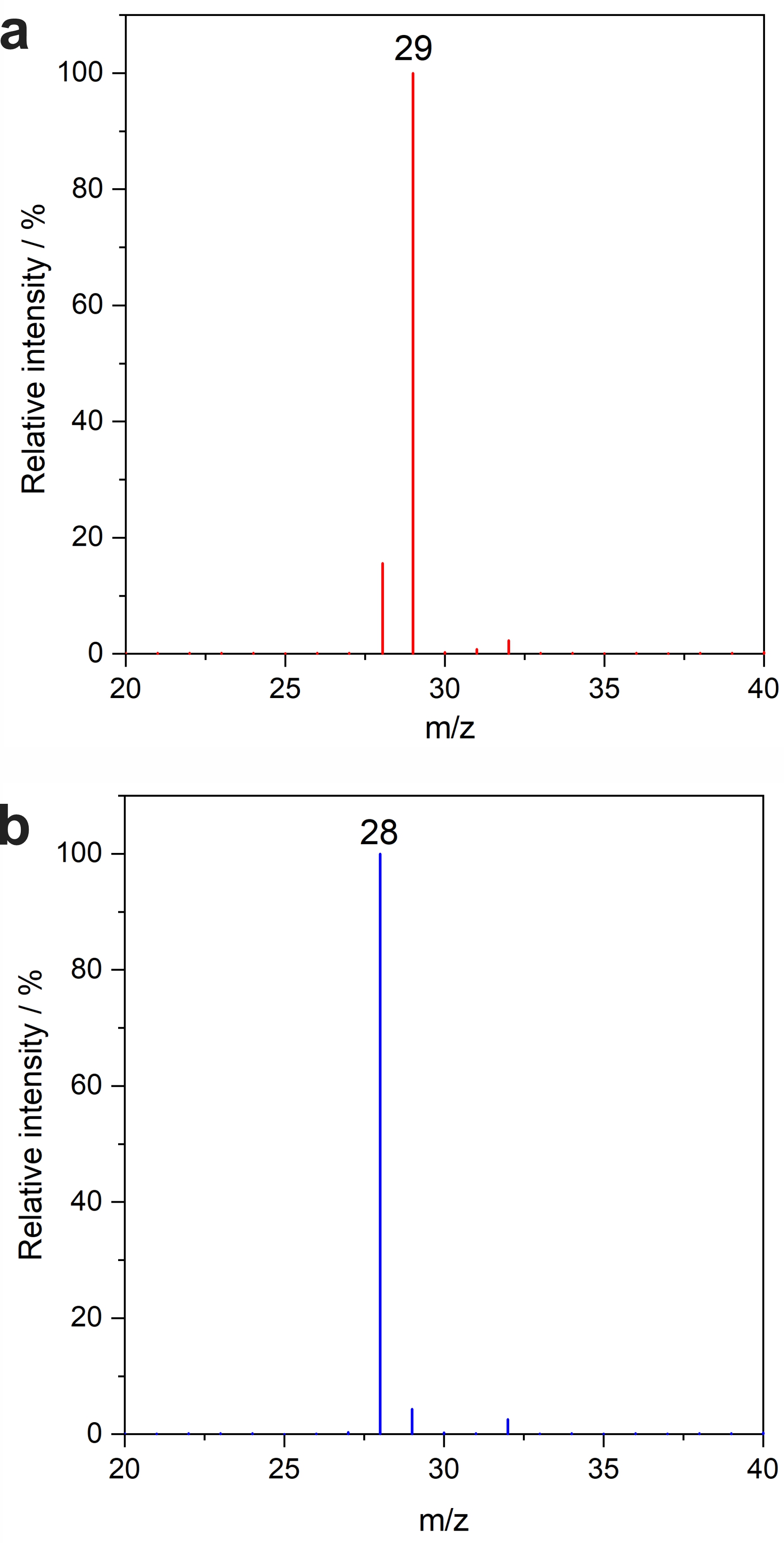


**Supplementary Figure S37.** Mass spectra of the gas product with ^13^CO_2_ (99.0% ^13^C) (a) and ^12^CO_2_ (b) as the reactant, respectively, measured at −0.73 V vs RHE. It can be seen that when ^13^CO_2_ is used, ^13^CO is the main product, suggesting that the CO product is converted from the inlet CO_2_ gas.


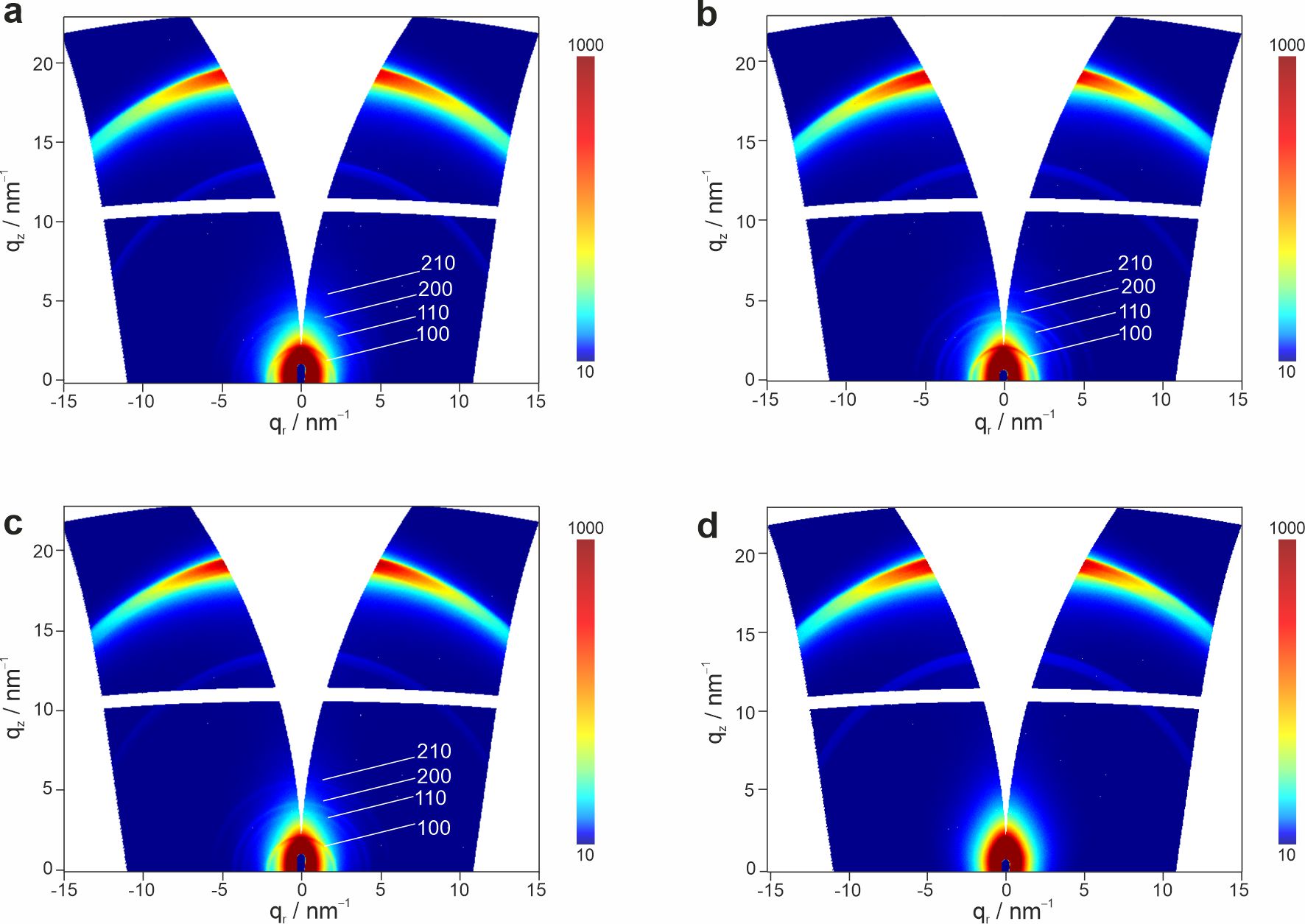


**Supplementary Figure S38.** GIWAXS 2D patterns of the electrodes for electrochemical CO_2_ reduction measurements. (a) is a pristine electrode without performing any electrochemical measurement. (b) and (c) are the electrodes after measured at −0.63 V vs RHE and −0.83 V vs RHE for 55 minutes, respectively. To avoid COF pore collapse due to water evaporation, the electrodes of b and c were dried by supercritical CO_2_. (d) is a control electrode that only contains carbon black. The result indicates that TPB-MeOTP COF reflections are maintained after the electrochemical measurements. The control sample only shows the typical reflections of carbon black, while does not exhibit the reflections of COFs.

**
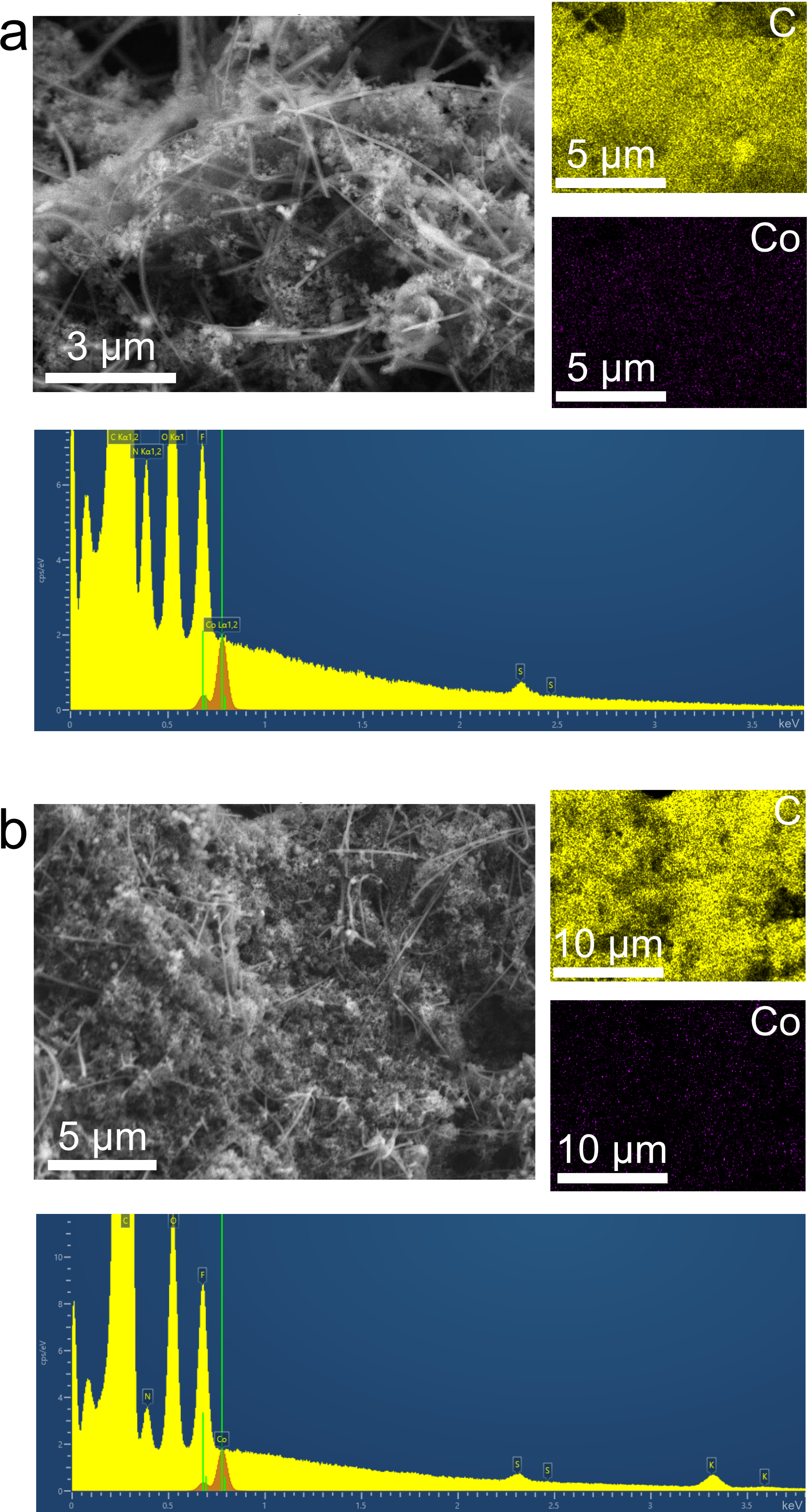
**

**Supplementary Figure S39.** SEM images and EDX mapping (carbon and cobalt) of the electrodes for electrochemical CO_2_ reduction measurements. (a) is taken from a pristine electrode without performing any electrochemical measurement (scale bar: 2.5 μm); (b) is taken from an electrode after controlled potential electrolysis for 55 minutes at -0.93 V vs RHE (scale bar: 5 μm). The images show that TPB-MeOTP-CNT:CoTPyP is highly mixed with carbon black on the electrode. No significant cobalt aggregates can be seen on the electrode even when operated at very negative potential (-0.93 V vs RHE), suggesting that no cobalt nanoparticles are transformed from the CoTPyP molecular catalyst during electrolysis.

**
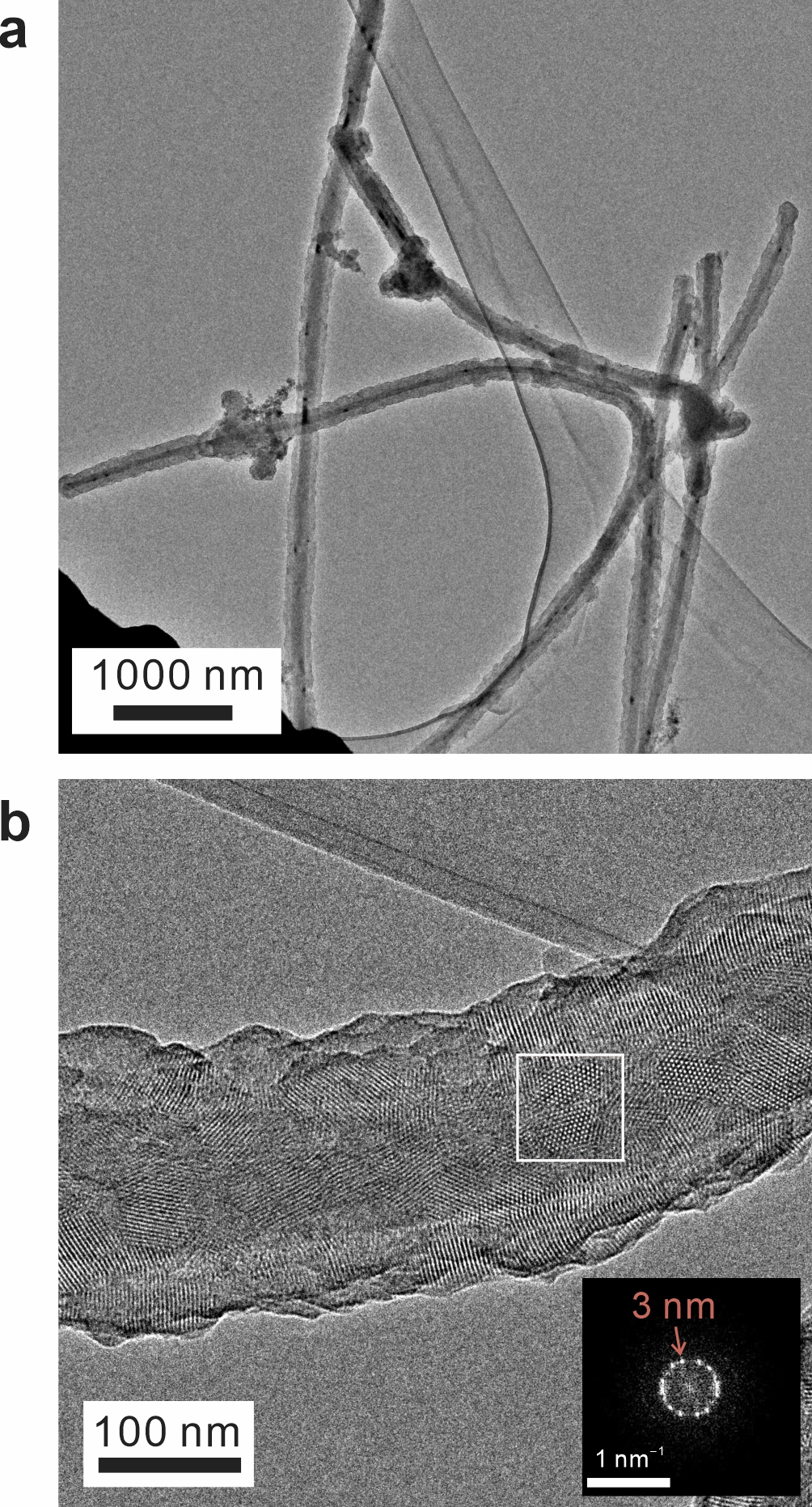
**

**Supplementary Figure S40.** BF-TEM images of the electrode after measured at −0.63 V vs RHE for 55 minutes with lower magnification (a) and higher magnification (b). The images show that both the core-shell nanohybrid structures and the reflection of TPB-MeOTP COF are retained after the electrolysis, suggesting the excellent stability of the samples.


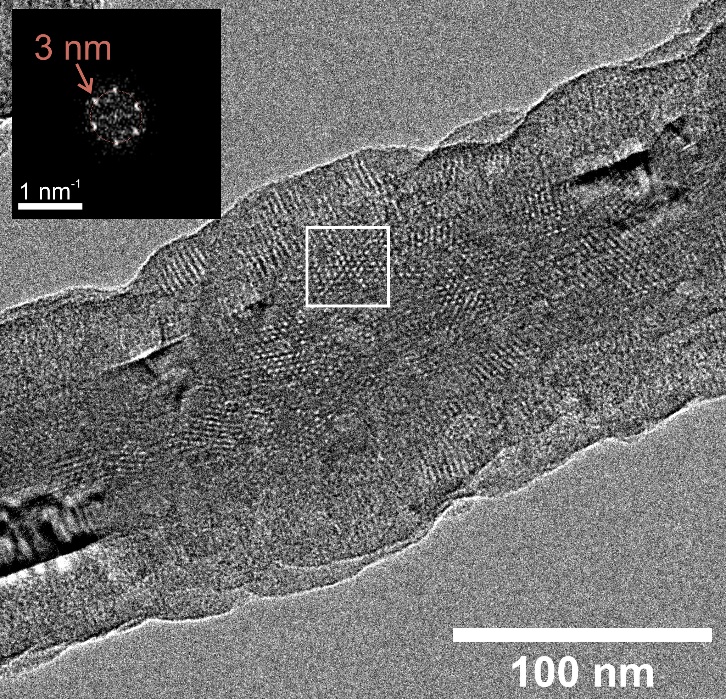


**Supplementary Figure S41.** TEM image of TPB-MeOTP-CNT:CoTPyP electrode after 15 h continuous electrochemical CO_2_ reduction operation.


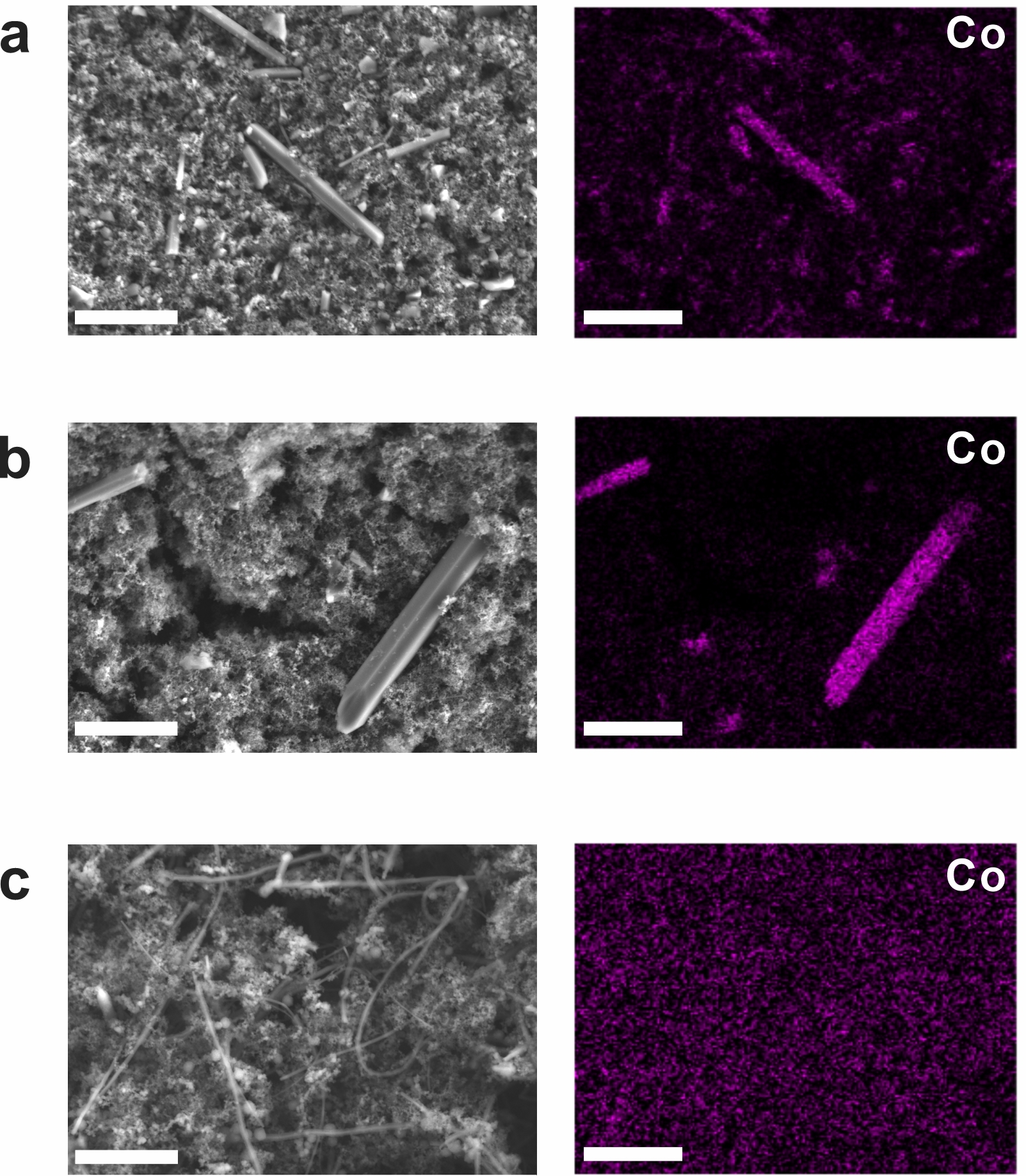


**Supplementary Figure S42.** SEM images and EDX maps of electrodes for electrochemical CO_2_ reduction, with (a) the CoTPyP : CB mass ratio of 1 : 1 (scale bar: 5 μm), (b) the CoTPyP : CB mass ratio of 0.1 : 1 (scale bar: 5 μm), and (c) TPB-MeOTP-CNT:CoTPyP : CB with the mass ratio of 1 : 1 (scale bar: 2.5 μm). CB: carbon black. The images show that CoTPyP forms aggregates when directly mixed with CB. In these cases, the CoTPyP molecules inside the aggregates are inaccessible for CO_2_ and electrolyte, thus leading to reduced utilization of the catalytic centers. In comparison, no visible cobalt aggregates are observed for TPB-MeOTP-CNT:CoTPyP, facilitating an increase in the catalytic center utilization.


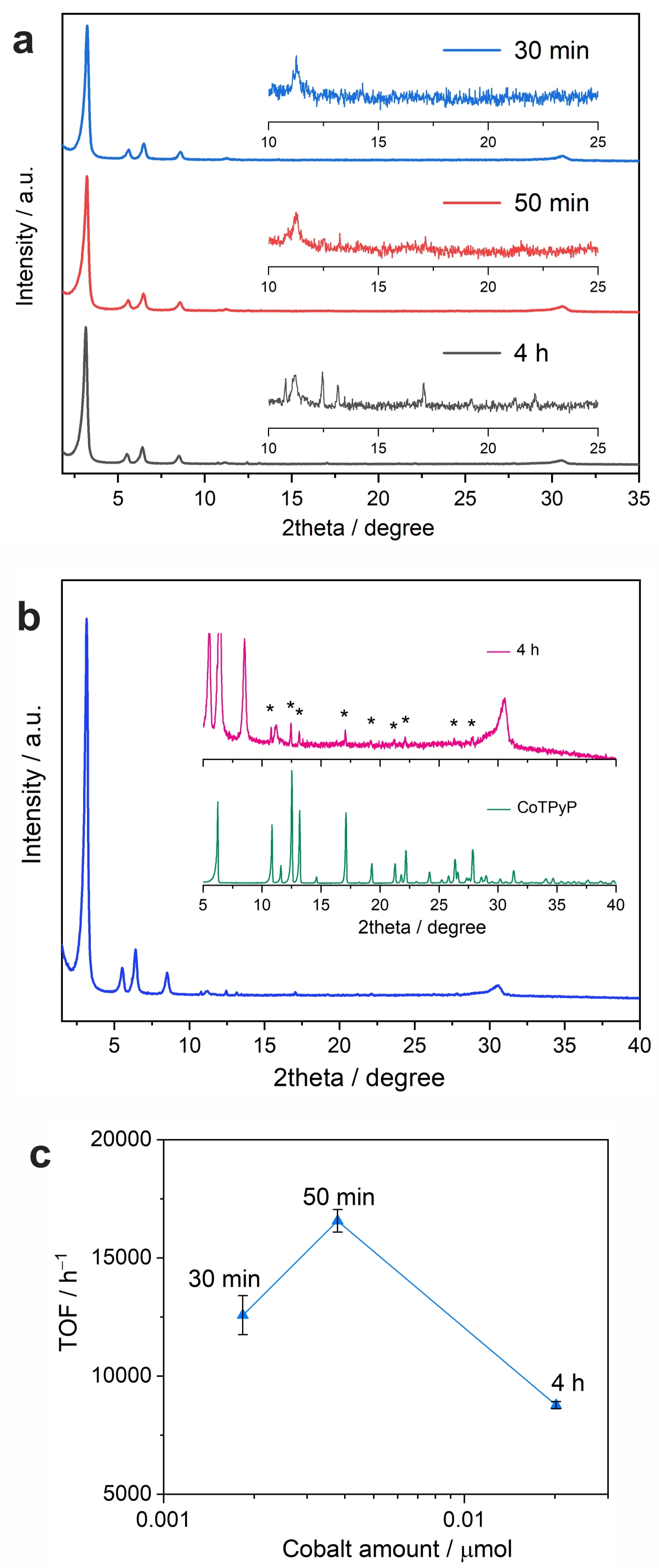


**Supplementary Figure S43.** The influence of CoTPyP loading time on the formation of CoTPyP aggregates and the electrochemical CO_2_ reduction performance. (a) PXRD patterns of TPB-MeOTP-CNT:CoTPyP obtained with different loading times: 30 min, 50 min and 4 hours. (b) PXRD patterns of TPB-MeOTP-CNT:CoTPyP prepared by 4 h loading and CoTPyP powder as a reference. (a) and (b) indicate that 4 h loading offers the formation of CoTPyP aggregates, while with reducing the loading time to 50 min and 30 min, the CoTPyP aggregate peaks are not present in the PXRD patterns. The cobalt amount in TPB-MeOTP-CNT:CoTPyP obtained with the loading time of 30 min, 50 min and 4 hours is 0.04 wt%, 0.09 wt% and 0.5 wt%, respectively, determined by ICP-OES. (c) The influence of CoTPyP loading time on the CO production turnover frequencies (TOFs) at ‒0.83 V vs RHE. It can be seen that 4 h loading time that contains CoTPyP aggregates affords the lowest CO TOF, implying CoTPyP aggregates are not the primary active species for TPB-MeOTP-CNT:CoTPyP.


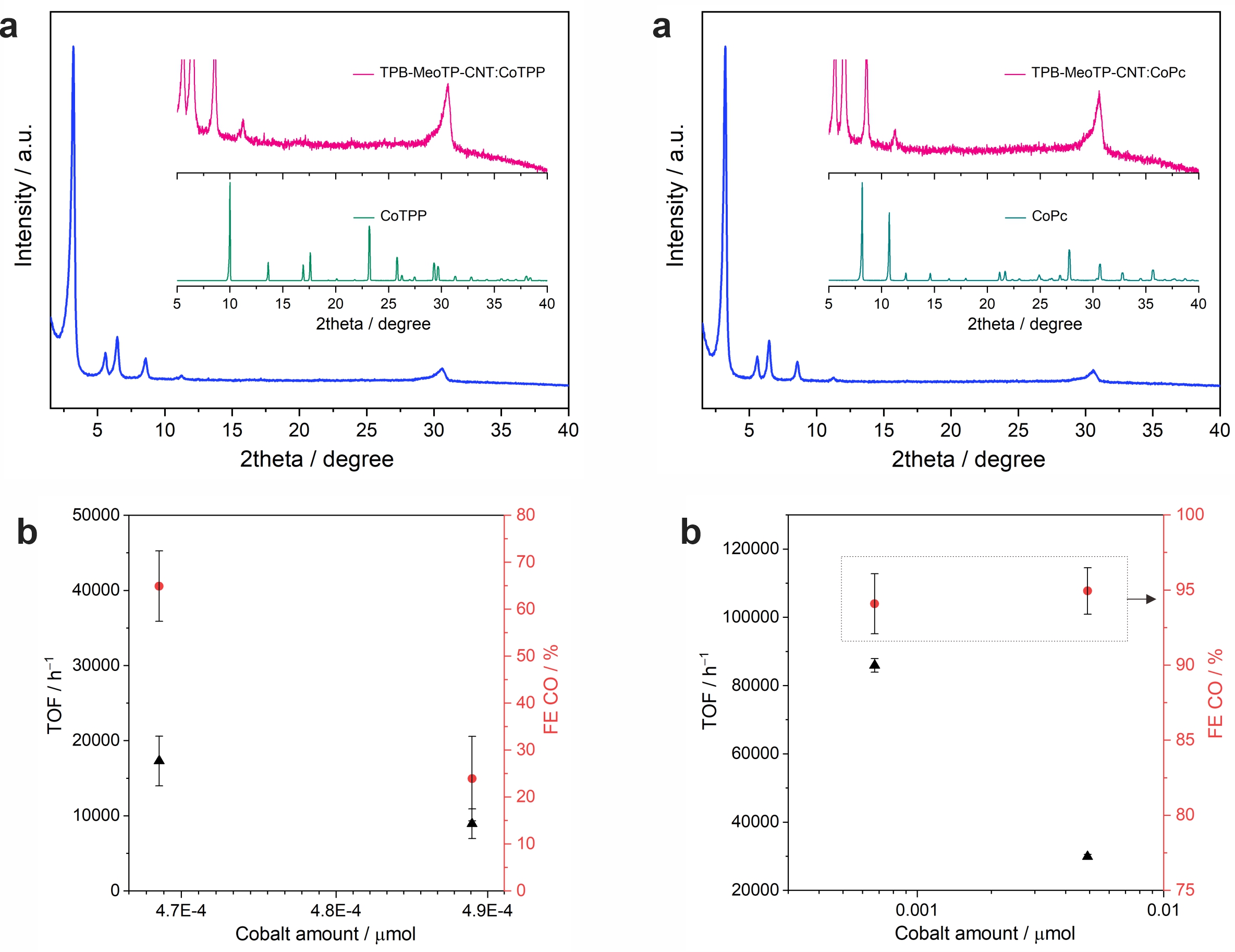


**Supplementary Figure S44.** PXRD pattern (a) of TPB-MeOTP-CNT:CoPc, and TOFs and FE(CO) (b) of TPB-MeOTP-CNT:CoPc for electrochemical CO_2_ reduction at −0.83 V vs RHE. The absence of CoPc aggregate peaks in the PXRD pattern of TPB-MeOTP-CNT:CoPc indicates that there is no CoPc aggregates in TPB-MeOTP-CNT:CoPc. The cobalt amount is determined to be 0.02 wt% by ICP-OES. The highest TOF obtained by TPB-MeOTP-CNT:CoPc is 85918 h^−1^ with the FE(CO) of 94%.


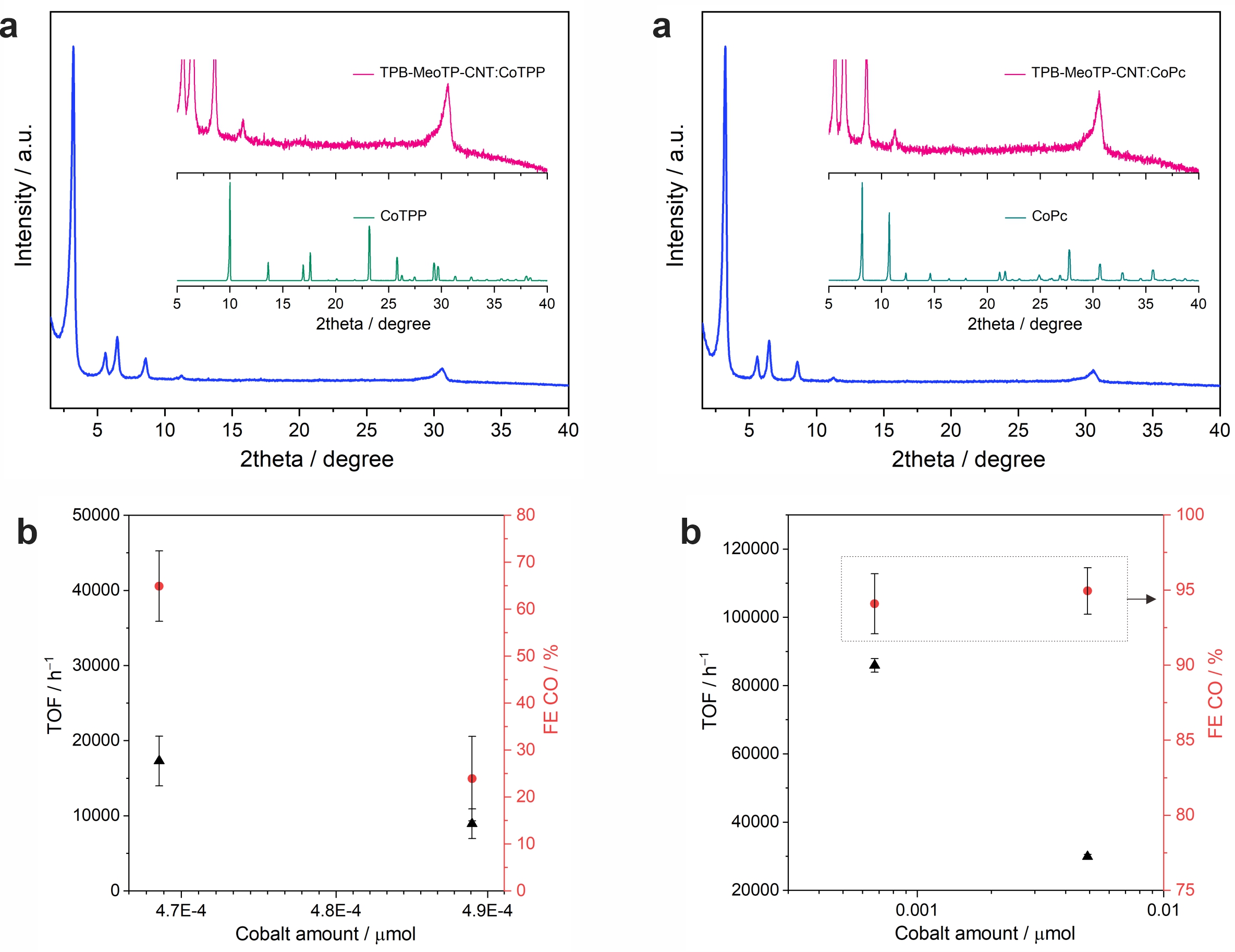


**Supplementary Figure S45.** PXRD pattern (a) of TPB-MeOTP-CNT:CoTPP, and TOFs and FE(CO) (b) of TPB-MeOTP-CNT:CoTPP for electrochemical CO_2_ reduction at −0.83 V vs RHE. Similar to TPB-MeOTP-CNT:CoPc, the CoTPP aggregate peaks are not present in the PXRD pattern of TPB-MeOTP-CNT:CoPc. The cobalt amount is determined to be 0.01 wt% by ICP-OES. The highest TOF obtained by TPB-MeOTP-CNT:CoPc is 17294 h^−1^ with the FE(CO) of 65%.


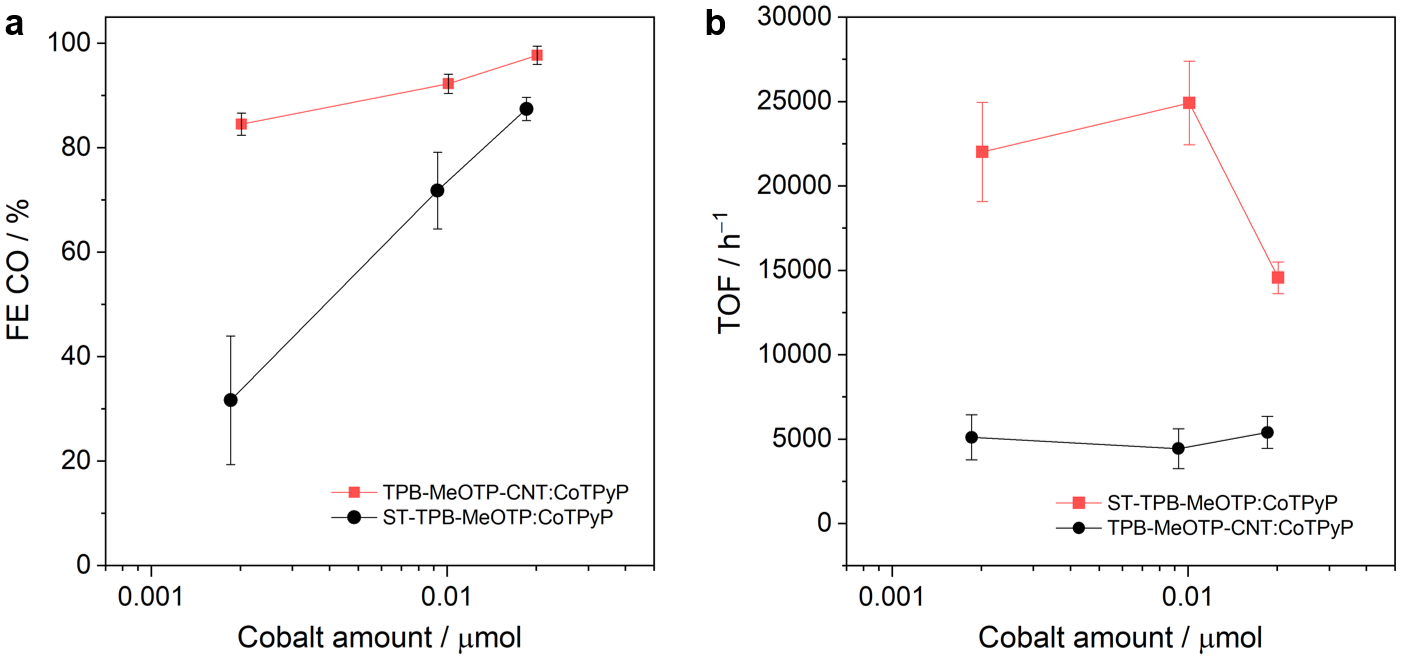


**Supplementary Figure S46.** Comparison of electrocatalytic CO_2_ reduction performance. CoTPyP physisorbed TPB-MeOTP-CNT (red) and TPB-MeOTP COF prepared in a solvothermal reaction (black) were compared. CO production Faradaic efficiencies (FE CO) and CO production turnover frequencies (TOF) at ‒0.83 V vs RHE, respectively. Standard deviations were obtained from four measurements. The average cobalt amount of solvothermal TPB-MeOTP COF containing physisorbed CoTPyP is 0.46 wt%.

**Supplementary Table 1.** Catalytic performance of reported heterogeneous molecular catalysts for CO_2_-to-CO conversion in an H-type cell.

| Catalyst | Category | FE(CO) | E  (V vs RHE) | TOF  (h^−1^) | Electrolyte | Ref. |
| --- | --- | --- | --- | --- | --- | --- |
| TPB-MeOTP-CNT:CoTPyP | COFs | 92% | ‒0.83 | 24914 | 0.5 M KHCO_3_ | **This work** |
| TPB-MeOTP-CNT:CoPc | COFs | 94% | ‒0.83 | 85918 | 0.5 M KHCO_3_ | **This work** |
| TPB-MeOTP-CNT:CoTPP | COFs | 65% | ‒0.83 | 17294 | 0.5 M KHCO_3_ | **This work** |
| COF-366-Co | COFs | 90% | −0.67 | 98 | 0.5 M KHCO_3_ | Science **2015**, *349*, 1208 |
| COF-367-Co | COFs | 91% | −0.67 | 165 | 0.5 M KHCO_3_ | Science **2015**, *349*, 1208 |
| COF-367-Co (1%) | COFs | 40% | −0.67 | 756 | 0.5 M KHCO_3_ | Science **2015**, *349*, 1208 |
| CoPc-PDQ-COF | COFs | 96% | −0.66 | 11412 | 0.5 M KHCO_3_ | Angew. Chem. Int. Ed. **2020**, *59*, 16587 |
| CoPc-PI-COF-3 | COFs | 95% | −0.80 | 2160 | 0.5 M KHCO_3_ | Angew. Chem. Int. Ed. **2021**, *61*, e202114244 |
| CuPcF_8_-CoNPc-COF | COFs | 97% | −0.62 | 10332 | 0.5 M CsHCO_3_ | J. Am.Chem. Soc. **2021**, *143*, 18052 |
| COF_bpyMn_\|NT | COFs | 72% | −0.55 | 1100 | 0.5 M NaHCO_3_ | ACS Catal. **2021**, *11*, 7210 |
| 0.5NiPc-COF | COFs | 73% | −1.0 | 4713 | 0.5 M KHCO_3_ | ACS Appl. Mater. Interfaces **2023**, *15*, 37, 44384 |
| COF@CoPor | COFs | 94.3% | −0.6 | 4578 | 0.5 M KHCO_3_ | Small **2022**, *18*, e2200736 |
| Catalyst | Category | FE(CO) | E  (V vs RHE) | TOF  (h^−1^) | Electrolyte | Ref. |
| TFPc-PBBA-COF | COFs | 95% | −0.9 | 1695 | 0.5 M KHCO_3_ | Angew. Chem. Int. Ed.**2023**, *63*, e202317785 |
| N^+^-NH-COF | COFs | 82.8% | −1.0 | 9922 | 0.5 M KHCO_3_ | Nat. Commun. **2023**, *14*, 3800 |
| PA-Co-COF | COFs | 86.8% | −1.0 | 2416.2 | 0.5 M KHCO_3_ | ACS Catal. **2024**, *14*, 11076 |
| CoPc-PI-COF-1 | COFs | 93% | −0.7 | 277.2 | 0.5 M KHCO_3_ | J. Am. Chem. Soc. **2021**, 143, 7104 |
| CoPc-PI-COF-2 | COFs | 93% | −0.7 | 208.8 | 0.5 M KHCO_3_ | J. Am. Chem. Soc. **2021**, 143, 7104 |
| NiPc-COF | COFs | 96% | −1.1 | 3780 | 0.5 M KHCO_3_ | Small, **2020**, 16, 2005254 |
| COF-A | COFs | 91% | −0.78 | ~35 | 0.5 M KHCO_3_ | Adv. Mater. **2024**, *36*, 2313197 |
| COF-T | COFs | 58% | −0.78 | ~850 | 0.5 M KHCO_3_ | Adv. Mater. **2024**, *36*, 2313197 |
| MOF-1992 | MOFs | 80% | −0.60 | 1044 | 0.5 M KHCO_3_ | J. Am. Chem. Soc. **2019**, *141*, 17081 |
| NiPc-NiO_4_ | MOFs | 98.4% | −1.2 | 2603 | 0.5 M KHCO_3_ | Angew. Chem. Int. Ed.**2021**, *60*, 17108 |
| CoPc–Cu–NH | MOFs | 72% | −0.74 | 4140 | 0.2 M KHCO_3_ | J. Am. Chem. Soc. **2020**, *142*, 21656 |
| Catalyst | Category | FE(CO) | E  (V vs RHE) | TOF  (h^−1^) | Electrolyte | Ref. |
| CoPc-Cu-O | MOFs | 85% | −0.74 | 2268 | 0.2 M KHCO_3_ | J. Am. Chem. Soc. **2020**, *142*, 21656 |
| monoMOF-Co@GO | MOFs | 93% | −0.8 | 10600 | 0.5 M KHCO_3_ | Angew. Chem. Int. Ed. **2025**, *64*, e202505399 |
| CoTPP | Molecular catalysts | ~73% | −0.66 | 9900 | 0.5 M KHCO_3_ | Angew. Chem. Int. Ed. **2017**, 56, 6468 |
| CoPc-CN | Molecular catalysts | 96% | −0.63 | 14760 | 0.1 M KHCO_3_ | Nat. Commun. **2017**, *8*, 14675 |
| CoPc | Molecular catalysts | 92% | −0.63 | 9720 | 0.1 M KHCO_3_ | Nat. Commun. **2017**, *8*, 14675 |
| Coqpy | Molecular catalysts | 99% | −0.58 | 43200 | 0.5 M NaHCO_3_ | Angew. Chem. Int. Ed. **2018**, 57, 7769 |
| CoPc1 | Molecular catalysts | 92% | −0.68 | 14688 | 0.5 M NaHCO_3_ | *Nat. Commun.* **2019**, 10, 3602 |
| CoPc2 | Molecular catalysts | 93% | −0.68 | 24516 | 0.5 M NaHCO_3_ | *Nat. Commun.* **2019**, 10, 3602 |
| CoCPY | Molecular catalysts | 96% | −0.70 | 34524 | 0.1 M KHCO_3_ | Angew. Chem. Int. Ed. **2020**, 59, 17104 |
| NiPc-OMe-MDE | Molecular catalysts | 100% | −0.64 | 10440 | 0.5 M KHCO_3_ | *Nat. Energy*. **2020**, 5, 684 |

**3. Reference**

1. Smith, B. J.; Parent, L. R.; Overholts, A. C.; Beaucage, P. A.; Bisbey, R. P.; Chavez, A. D.; Hwang, N.; Park, C.; Evans, A. M.; Gianneschi, N. C.; Dichtel, W. R., Colloidal Covalent Organic Frameworks. *ACS Central Science* **2017,** *3* (1), 58-65.

2. Han, J.; An, P.; Liu, S.; Zhang, X.; Wang, D.; Yuan, Y.; Guo, J.; Qiu, X.; Hou, K.; Shi, L.; Zhang, Y.; Zhao, S.; Long, C.; Tang, Z., Reordering d Orbital Energies of Single‐Site Catalysts for CO2 Electroreduction. *Angewandte Chemie International Edition* **2019,** *58* (36), 12711-12716.

3. Lin, K.-J., SMTP-1: The First Functionalized Metalloporphyrin Molecular Sieves with Large Channels. *Angewandte Chemie International Edition* **1999,** *38* (18), 2730-2732.

4. Ballirano, P.; Caminiti, R.; Ercolani, C.; Maras, A.; Orrù, M. A., X-ray Powder Diffraction Structure Reinvestigation of the α and β Forms of Cobalt Phthalocyanine and Kinetics of the α → β Phase Transition. *Journal of the American Chemical Society* **1998,** *120* (49), 12798-12807.

5. de Melo, C. C.; Moreira, W. d. C.; Martins, T. J.; Cordeiro, M. R.; Ellena, J.; Guimarães, F. F.; Martins, F. T., Saddle-shaped macrocycle distortion and symmetry decrease in cobalt (II) meso-tetraphenylporphyrin: Structure of a dichloromethane solvate and DFT calculations. *Journal of Molecular Structure* **2014,** *1076*, 468-474.
